# Synthetic Par polarity induces cytoskeleton asymmetry in unpolarized mammalian cells

**DOI:** 10.1016/j.cell.2023.08.034

**Published:** 2023-09-28

**Authors:** Joseph L. Watson, Lara K. Krüger, Ariel J. Ben-Sasson, Alice Bittleston, Marta N. Shahbazi, Vicente Jose Planelles-Herrero, Joseph E. Chambers, James D. Manton, David Baker, Emmanuel Derivery

**Affiliations:** 1 MRC Laboratory of Molecular Biology, Francis Crick Avenue, Cambridge, UK; 2 Department of Biochemistry, University of Washington, Seattle, WA 98195, USA; 3 Institute for Protein Design, University of Washington, Seattle, WA 98195, USA; 4 Howard Hughes Medical Institute, University of Washington, Seattle, WA 98195, USA; 5 Cambridge Institute for Medical Research, Department of Medicine, University of Cambridge, Hills Rd, Cambridge, UK

## Abstract

Polarized cells rely on a polarized cytoskeleton to function. Yet, how cortical polarity cues induce cytoskeleton polarization remains elusive. Here, we capitalized on recently established designed 2D protein arrays to ectopically engineer cortical polarity of virtually any protein of interest during mitosis in various cell types. This enables direct manipulation of polarity signaling and the identification of the cortical cues sufficient for cytoskeleton polarization. Using this assay, we dissected the logic of the Par complex pathway, a key regulator of cytoskeleton polarity during asymmetric cell division. We show that cortical clustering of any Par complex subunit is sufficient to trigger complex assembly and that the primary kinetic barrier to complex assembly is the relief of Par6 autoinhibition. Further, we found that inducing cortical Par complex polarity induces two hallmarks of asymmetric cell division in unpolarized mammalian cells: spindle orientation, occurring via Par3, and central spindle asymmetry, depending on aPKC activity.

## Graphical abstract

**Graphical abstract:**
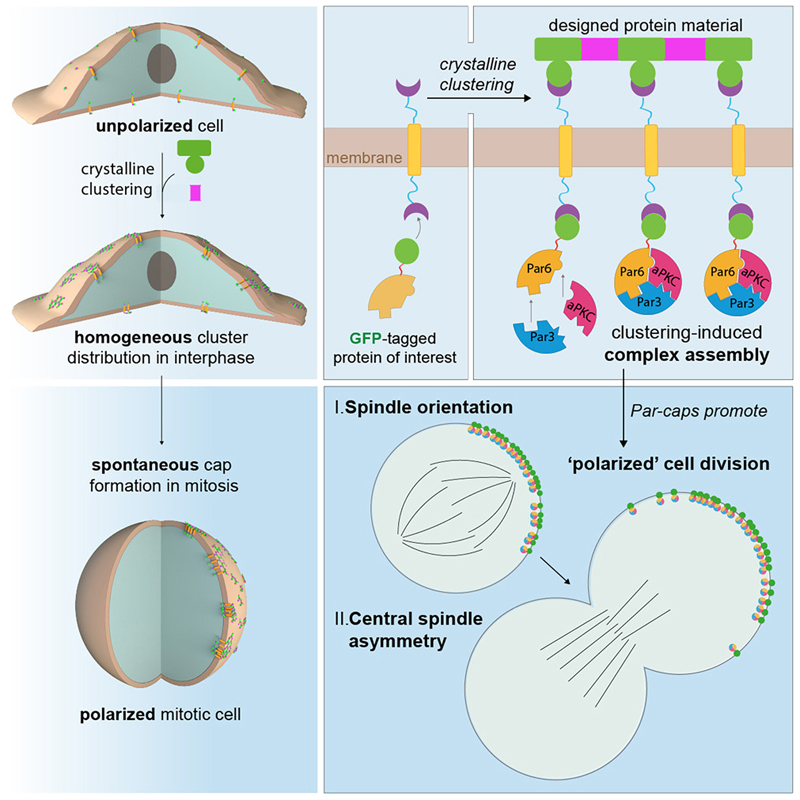

## Introduction

Intrinsic asymmetric cell division gives rise to two daughter cells that inherit different fate determinants, thereby acquiring different fates. The cytoskeleton of asymmetrically dividing cells is profoundly polarized, with this polarity being controlled by asymmetric signaling at the cell cortex. For instance, the mitotic spindle orients along cortical polarity cues to break the cell’s rotational symmetry.^1^ This ensures asymmetric segregation of cortical fate determinants to only one daughter cell, as well as their biased access to the stem cell niche. Another conserved feature of asymmetric cell division is the polarized trafficking of signaling organelles to enhance the robustness of asymmetric cell fate determination.^2–9^ This also relies on a polarized cyto-skeleton. In flies, the anaphase spindle midzone, also known as the central spindle, has been shown to be asymmetric, with a higher microtubule density on one side than the other, which in turn biases the polarized trafficking of endosomes containing cell fate determinants toward one daughter cell.^5,10^ In addition to the polarization of the microtubule network, polarity cues have been proposed to bias the motor content at the surface of endosomes, therefore inducing their polarized trafficking independently of central spindle asymmetry.^9^ Understanding the mechanisms underlying asymmetric partitioning of cell fate determinants driven by polarity cues, in particular understanding the molecular link between cortical polarity cues and cytoskeleton symmetry breaking, is a long-standing question in the field.

*In vitro* reconstitution with purified components has consistently led to notable leaps in our molecular understanding of cellular processes, as it allows one to delineate what is *sufficient* for a given phenomenon to occur rather than simply what is *required*. Similarly, the *in vivo* reconstitution of intracellular polarity in otherwise unpolarized cells has been a long-standing goal of the field to increase our molecular understanding of cell polarity and asymmetric cell division, in particular, to determine how cortical signaling induces cytoskeletal symmetry breaking.^11–17^ Indeed, engineering cells where a specific signaling cue can be selectively polarized would enable one to bypass feedback loops with other pathways and thus to specifically interrogate which aspects of cytoskeletal symmetry breaking that specific cue is sufficient to induce.

Over the years, several methods have been pioneered to reconstitute cortical polarity in unpolarized cells. Conceptually, they can be sorted into four categories: (1) beads covered with signaling ligands inducing an endogenous polarity cascade,^11^ (2) hijacking cell-cell junctions to create an artificial polarity,^12,13^ (3) light-induced or constitutive subcellular oligomerization to locally assemble a cortical polarity cue,^14–16,18^ and (4) adhesive micropatterns that impose a polarized shape to the cell.^17^ Collectively, these methods have provided remarkable insights into some molecular aspects of the various spindle orientation pathways, both in flies and mammals. However, so far, none of these methods have been adapted to reconstitute the assembly, activity, and polarization of the Par complex, one of the main polarity cues during asymmetric cell division, thus precluding our molecular understanding of how it drives cytoskeleton symmetry breaking.

The Par complex comprises three well-conserved subunits, namely Par3, Par6, and the atypical protein kinase C (aPKC).^19^ At mitosis onset, the assembled complex coalesces into a cap at the apical cell cortex and restricts other fate determinants to the basal cortex, thus establishing the apical-basal polarity axis in naturally polarized cells.^1^ Subsequently, the Par cap is required for several processes allowing the cell to eventually divide asymmetrically, for instance, for alignment of the mitotic spindle with the cell’s polarity axis.^1^ Yet, it is still unclear what triggers Par complex assembly and which aspects of cytoskeleton polarization the Par complex is sufficient to induce in the absence of other polarity cues.

We envisioned that the reason why the link between the Par complex and cytoskeleton symmetry breaking could not be unraveled by previous polarity reconstitution methods was because none of them simultaneously combined the requirements needed to quantitatively investigate both the assembly of cortical polarity cues (i.e., inducible and fast) and the direct effects of engineered polarity during mitosis (i.e., works at the single cell level, control of the molecular composition of the cap, high-throughput; see also Table 1 for further comparison of each method).

Here, we capitalize on protein design to establish a general method to reconstitute cortical polarity of virtually any protein during division in various cell types. We then applied this assay to quantitatively interrogate the assembly and outputs of the Par complex pathway in mammalian cells. We show that Par complex assembly is triggered by clustering of any of its subunits and that the main kinetic barrier to Par complex assembly is the relief of Par6 autoinhibition. We then show that Par complex polarity is sufficient to induce three key hallmarks of asymmetric cell division in unpolarized mammalian cells, namely spindle orientation, asymmetric segregation of cortical components, and central spindle asymmetry. These events can be molecularly untangled. Spindle orientation is determined by Par3 and does not require the kinase activity of aPKC. By contrast, we uncover that central spindle asymmetry depends on the asymmetric kinase activity of aPKC. Furthermore, we demonstrate that the downstream molecular mechanism of central spindle symmetry breaking is conserved from flies to mammals, with the microtubule regulators Camsap3 and Kif2A/Kif2B/MCAK having inherited the ancestral function of Patronin and Klp10A, respectively.

## Results

### Induction of cell polarity from the outside using designed protein arrays

**Figure 1:**
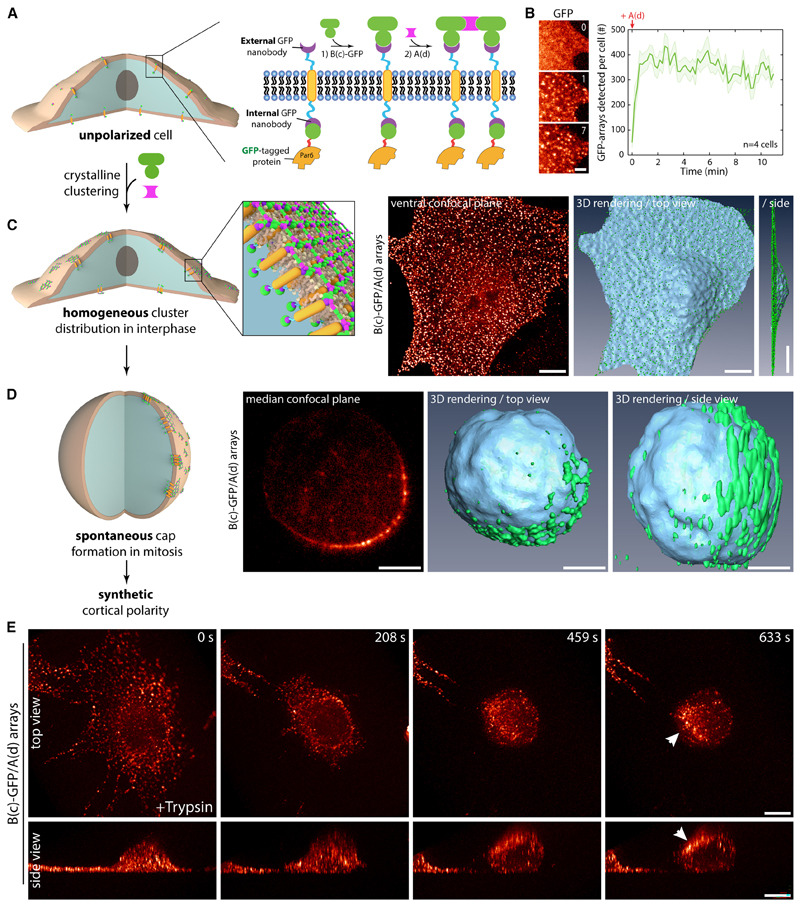
Artificial symmetry breaking of the cortex using protein design (A) Principle of the experiment: 3T3 cells stably co-expressing GBP-TM-GBP and a GFP-fused target were incubated with B(c)GFP, then A(d), to induce rapid clustering. (B) Left: cells processed as in (A) imaged by SDCM. Elapsed time: min. Right: mean ± SEM number of clusters per cell over time in cells. (C) Left: protein clusters are homogenously distributed at the cell cortex in interphase. Right: cells as in (B) were imaged 10 min after clustering by SDCM (middle: single confocal plane; right: three-dimensional [3D] rendering). (D) Array assembly was triggered at the surface of 3T3 cells stably expressing GFP-LGN and GBP-TM-GBP, then cells were stalled in mitosis for >12 h with nocodazole before SDCM imaging (left: single confocal plane; right: 3D rendering). (E) Arrays were assembled at the surface of 3T3 cells stably expressing GFP-aPKC and GBP-TM-GBP, and cells were imaged by oblique plane light sheet microscopy, while cell rounding was induced by trypsin. The PopRed lookup table was applied to all single-channel images in this figure. Scale bars; 10 μm in (C) and (E); 5 μm in (D); and 2 μm in (B). See also Figures S1 and S2.

To shed light on the molecular mechanisms underlying assembly and function of cortical polarity cues in mammals, we sought to capitalize on designed protein arrays^20^ to reconstitute inducible cortical polarity in otherwise unpolarized NIH/3T3 fibroblasts. Our method relies on the stable expression of a synthetic transmembrane construct that is rapidly clustered *from the outside* through assembly of a designed two-dimensional (2D) protein material (Figure 1). This material consists of two components, A(d) and B(c)-GFP, where B(c)-GFP binds to the transmembrane segment (TM) via an external anti-GFP nanobody (GBP for “GFP-binding peptide”; Kirchhofer et al.^21^; Figure 1A), and A(d) clusters the B(c) component into a hexagonal array^20^ (Figure 1A). Clustering with this method is fast (~20 s; Figure 1B), efficient (~80 targets per diffraction-limited spot^20^), and occurs simultaneously and homogeneously over the cell surface in interphase (Figures 1C and S1A–S1C; see Video S1 for demonstration that arrays assemble homogenously at the cortex, in particular, at the ventral surface). The resulting arrays are stable at the cell surface, as the material has been engineered to evade endocytosis.^20^ Although we previously studied array assembly over short timescales,^20^ we now find that over longer incubation periods, when cells round up during mitosis, these clusters coalesce and form cortical caps (Figure 1D; Video S2).

The spontaneous cap formation is not due to the reagent used to stall cells in mitosis, as partitioning is evident in non-stalled cells entering mitosis under physiological conditions (Figure S2A). Similarly, this phenomenon is not restricted to 3T3 fibroblasts and also occurred in epithelial U2OS cells (Figure S2B; Video S3). Further, cap formation seems to correlate with a spherical geometry rather than mitosis: artificially rounding up cells using trypsin leads to cap formation in interphase (Figures 1E and S2C). This phenomenon occurs within minutes (Figure 1E; Video S4). The correlation between cap formation and spherical shape suggested that our polymer should induce caps at the surface of any round cell, irrespective of its cell cycle stage. We validated this prediction using mouse embryonic stem (ES) cells, which are round and unpolarized in interphase, and robustly formed caps (Figure S2D). Similarly, caps formed on spherical *Drosophila* S2 cells adapted to liquid culture (Figure S2E). Furthermore, cap formation is not specific to our transmembrane construct, as it occurs with native receptors, such as Notch, when functionalizing the clustering material with its ligand, delta like canonical Notch ligand 4 (DLL4) (Figure S2F). Finally, cap formation efficiency does not markedly depend on the protein targeted to the transmembrane construct (Figure S2G).

Caps are not constituted of a reticulated network of arrays but are instead quite fluid. Indeed, cap formation is reversible, as caps dissolved when cells respread after mitosis (Figure S2H). Along the same lines, arrays had confined motility within the cap and changed neighbors within it (Figure S2I). This is expected as arrays are assembled in the presence of an excess of A(d), which prevents their fusion, as arrays saturated with A(d) on all edges cannot interact with each other. To investigate the mechanism by which these caps form, we used a membrane tension/lipid packing probe, Flipper-TR,^22^ and found that the Flipper-TR fluorescence lifetime was significantly increased in the membrane underlying the formed cap compared with the surrounding membrane (Figures S2J–S2L). This suggests that clusters exert local changes in membrane tension/lipid packing or, alternatively, that the clusters have affinity for existing zones of high cortical tension/lipid packing. In both cases, this would result in a tendency of the arrays to stay together once they meet, particularly since bulk endocytosis, to which arrays are mostly immune, is a major driver of membrane remodeling.^23^

Although the exact physics of cap formation is beyond the scope of this study, the remarkable property of the arrays to coalesce into caps when cells round up allows the reconstitution of cortical polarity of virtually any protein of interest within an entire population of dividing cells.

### Clustering of any Par complex subunit induces slow complex assembly

We first took advantage of the protein arrays to investigate the Par complex assembly pathway. The core Par complex is composed of three conserved subunits, Par3, Par6, and the kinase aPKC.^19^ Although each Par complex subunit can bind to the two others, the triple complex is not always constitutively assembled,^24,25^ and it is unclear how all subunits come together. NIH/3T3 cells are a good model system to study this phenomenon because their endogenous core Par complex is unassembled with very little co-localization between Par3 and aPKC, both at the cortex and in the cytosol, except at inter-cell boundaries where the Par complex sometimes appears as punctate structures when cells are plated at very high confluency (Figures S3A–S3C).

To probe the possible Par complex assembly pathways and which molecular interactions are critical in each case, we systematically clustered each subunit (or mutants thereof) and quantitatively followed whether the two other subunits are recruited to these clusters at the endogenous level (see construct list in Table 2). We performed this analysis in interphase, when clusters are sparse, rather than in mitosis, when caps are formed, to temporally separate Par complex assembly from its downstream effects in mitosis. This capitalizes on the robustness of our clustering method, which allows us to make quantitative measurements on thousands of mostly identical crystalline arrays (see STAR Methods).

To cluster Par complex subunits, we used a bicistronic expression system where stably expressed, GFP-tagged Par proteins are relocalized to an independently expressed transmembrane construct via an internal anti-GFP nanobody (Figure 1A). This indirect arrangement ensured comparable expression of the transmembrane construct between stable cell lines and therefore comparable cluster size,^20^ as opposed to direct fusion of Par complex proteins to the transmembrane construct (Figures S1D-S1F). Note that these clusters are not three-dimensional aggregates or condensates, as both the spacing and orientation of each protein of interest are defined by the crystal lattice of the material^20^ and are hence invariant to the internal target protein (inter-protein distance ~8 nm).

**Figure 2:**
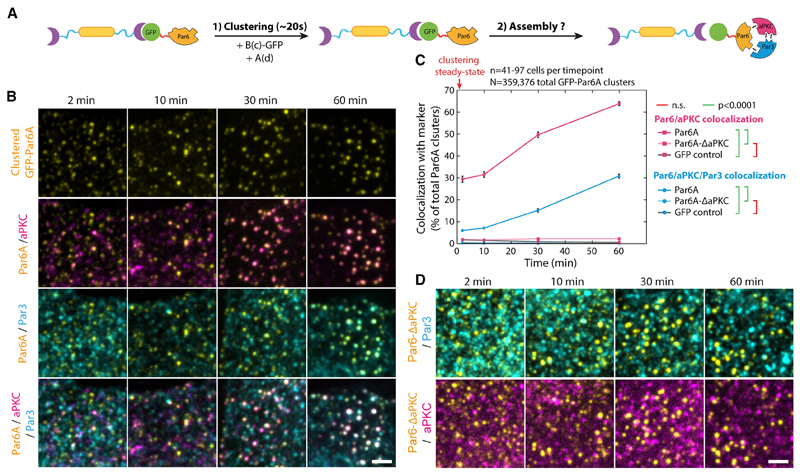
Sequential assembly of the core Par complex induced by clustering (A) Principle of the experiment: GFP-fused Par6A, or mutants thereof, were clustered in 3T3 cells, and Par complex assembly was subsequently monitored. (B) Recruitment of endogenous aPKC and Par3 upon Par6A clustering monitored by immunofluorescence. (C) Mean ± SEM percentage of co-localization between clusters of indicated GFP-Par6A construct and endogenous aPKC over time, as well as triple colocalization between GFP-Par6A, endogenous aPKC, and endogenous Par3. Statistics: ANOVA2 using construct and timepoint as variables, followed by Tukey test (p value indicated). Clustering of GFP is provided as a negative control. (D) Recruitment of endogenous aPKC and Par3 to GFP-Par6A^ΔaPKC^ clusters monitored as above. Scale bars, 2 μm. See also Figures S3, S4, S5 and S6 and Table 2.

Although triple colocalization between Par6A, aPKC, and Par3 was low in baseline conditions (2 min post-clustering), artificially clustering Par6A was sufficient to induce Par complex assembly over 60 min (Figures 2A–2C, blue curve, S3D–S3H for controls, and S4B for split images). As the kinetics of array-induced clustering is fast compared with the kinetics of Par complex formation (~20 s versus ~1 h), this shows that Par complex assembly is a slow process, at least in our system. This was confirmed when we measured the intensity of aPKC per Par6A clusters (rather than the binary colocalization), which we found to also increase over time (Figure S4C). Significantly, Par complex assembly could also be triggered upon clustering of aPKC (Figure S5), or Par3 (Figure S6), with similar kinetics. Interestingly, different Par6 isoforms displayed slight differences in their ability to induce Par complex assembly upon clustering, with Par6A being the most and Par6B the least efficient (Figures S4D–S4F), which correlated with their ability to associate with Par complexes formed by aPKC clustering (Figures S4G–S4I). Thus, we focused on Par6A in the rest of this study and will refer to Par6A as Par6 for simplicity. Altogether, this establishes that clustering, a commonly reported feature of Par biology, per se drives assembly of the core Par complex and that clustering of any core subunit induces Par complex assembly with similar kinetics.

As each protein of the Par complex can bind to the two others, we sought to untangle the inter-subunit interactions required for assembly. Clustered Par6^ΔaPKC^, an established Par6 point mutant incapable of binding to aPKC,^26^ was unable to recruit either aPKC or Par3 (Figures 2C, 2D, S4B, and S4C; Table 2). Similarly, clustered aPKC^ΔPar6^, an established mutation abolishing Par6 binding,^26^ was also unable to recruit Par3 (Figures S5D and S5J; Table 2). These results demonstrate that the Par6-aPKC interaction is required for Par3 recruitment and, thus, assembly of the full Par complex (see section “rationale of Par complex assembly pathway” in STAR Methods for further details).

### Relieving Par6 autoinhibition is the rate-limiting step in Par complex assembly

One hypothesis to explain why Par complex assembly is slow in our system is that it involves post-translational modifications since aPKC-mediated phosphorylation of Par3 has been proposed to regulate Par complex assembly.^27–30^ However, established kinase hyperactive and dead mutants of aPKC, noted aPKC^active^ and aPKC^dead^, respectively, recruited Par3 to the same extent as the wild type (Figures S5C, S5F, S5G, and S5J), and clustering of Par3 phosphomimetic mutants for the phospho-regulated aPKC-binding site,^30^ only marginally affected assembly (Figure S6G; note that a second, phosphorylation-independent interaction between aPKC and Par3 has been reported in flies^31^). Altogether, this suggests that the kinase activity of aPKC is dispensable for mammalian Par complex assembly and thus cannot explain the observed delay in this process.

**Figure 3:**
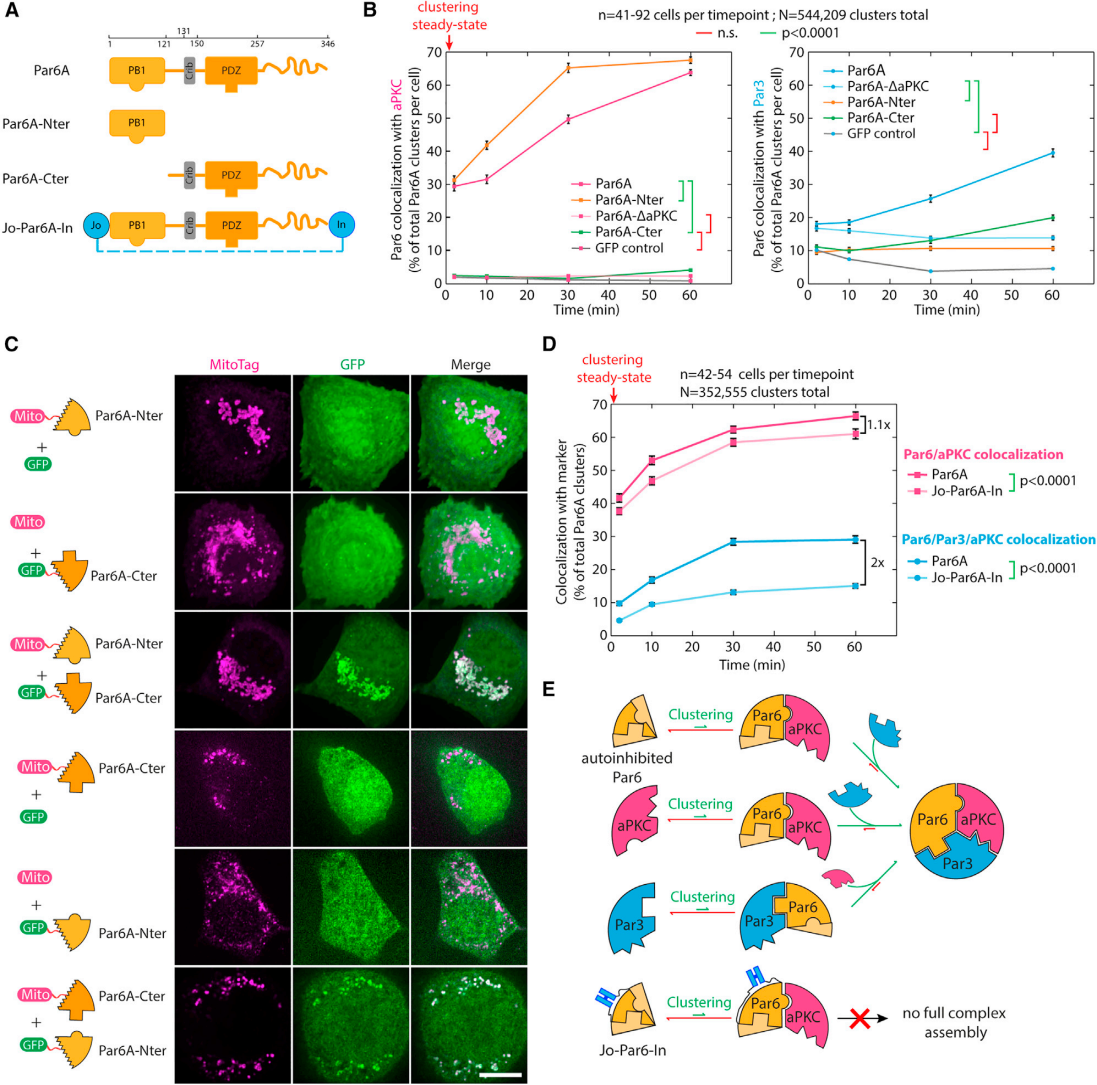
Relieving Par6 autoinhibition is the rate-limiting step in Par complex assembly (A) Par6A fragments used in this study. The N-terminal PB1 domain of Par6A binds to aPKC, while the C-terminal PDZ domain binds to Par3. The Jo domain, fused to the N terminus, covalently binds to the In domain, fused to the C terminus.^32^ (B) Mean (±SEM) percentage of colocalization between GFP-Par6A or Par6A mutant clusters and aPKC (left) or Par3 (right). Statistics: right: ANOVA to test interaction with time: Par6A: p < 0.0001; Par6A^Cter^: p < 0.0001; Par6A^Nter^: n.s.; Par6A^ΔaPKC^: p < 0.01. left: ANOVA2 using construct and time point as variables (p value of construct effect indicated). Par6A/GFP curves are the same as in Figure 2, reproduced here for convenience. See Figure S7B for representative images. (C) Par6A^Nter^ binds Par6A^Cter^. 3T3 cells expressing cytosolic GFP-Par6A^Cter^, and Par6A^Nter^ tethered to the mitochondria, or vice versa, were imaged by SDCM. Maximum intensity z-projection (MIP) is shown. Par6A^Nter^ recruits Par6A^Cter^ to mitochondria, and vice versa, suggesting that Par6A folds on itself (see also Figure S7C for further controls). (D) GFP-fused Par6A, or Jo-Par6-In, was clustered in 3T3 cells, and the recruitment of endogenous aPKC and Par3 was measured over time (mean ± SEM percentage of dual colocalization between GFP-Par6A clusters and aPKC [magenta] and triple colocalization between Par6A, aPKC, and Par3 [blue]). See Figure S7F for representative images. Statistics: ANOVA2 using construct and time point as variables (p value of construct effect indicated). Note that the Jo-Par6A-In profoundly inhibits assembly of the tripartite Par complex. (E) Preferred route for assembly of the core Par complex as a function of the clustered subunit (see also STAR Methods). Scale bars: 10 μm in (C). See also Figure S7 and Table 2.

We propose an alternative hypothesis where the slow assembly rate is due to Par6 autoinhibition, with aPKC-binding “opening” Par6 and revealing its Par3-binding domain. In line with this hypothesis, Par6A^Nter^ (AA1–121, harboring the aPKC-binding site) was able to recruit aPKC but not Par3, whereas clustering Par6A^Cter^ (AA121–346, harboring the Par3 binding site) was able to recruit Par3 but not aPKC (Figures 3A and 3B; see also Figure S7B for representative images). Consistent with this, aPKC recruitment by Par6^Nter^ was faster than Par6 full-length (Figure 3B), as expected if the slow aPKC recruitment was the result of the need to relieve Par6 autoinhibition. Lastly, Par6^Cter^ could recruit Par3, while the full-length Par6D^∆aPKC^ mutant could not (Figure 3B), suggesting that full-length Par6 indeed exists in an autoinhibited state, which can be relieved by aPKC binding.

The interaction between the two halves of Par6 was confirmed by relocalization experiments where mitochondria-targeted Par6^Nter^ robustly recruited cytosolic GFP-Par6^Cter^ to mitochondria and vice versa (Figure 3C; see also Figure S7C for narrowing down of the interaction region between the Phox and Bem1 domain (PB1) and PDZ domains of Par6, which is also supported by AlphaFold2^33^ prediction, Figure S7A). This interaction is likely direct, as when the two halves of Par6 were separately expressed in *Escherichia coli*, where homologs able to recapitulate indirect binding are unlikely to exist, GST-Par6^Nter^ could specifically pull-down PC-Par6^Cter^ (Figures S7D and S7E). Lastly, we reasoned that if Par6 autoinhibition is the underlying reason for the slow assembly kinetics, then a Par6 mutant biased toward autoinhibition should further slow down Par complex assembly. We thus flanked Par6 with the “Jo” and “In” domains, which are known to form a covalent bond with each other, although this reaction is generally not complete.^32^ As predicted, Par complex assembly took twice as long with Jo-Par6-In compared with Par6 (Figure 3D, blue curves; see also Figure S7F). Interestingly, Jo-Par6-In recruited aPKC with relatively normal kinetics compared with the wild type, again confirming that the direct interaction between aPKC and Par3 is not sufficient to recruit Par3 onto Par6 (Figure 3D, magenta curves).

In conclusion, we propose the following paradigm for Par complex assembly (Figure 3E): (1) clustering of any of the core sub-units can trigger assembly of the complex, (2) the rate-limiting step of assembly is the opening of an autoinhibited Par6, (3) the kinase activity of aPKC does not affect initial Par complex assembly, and (4) the direct binding between aPKC and Par3 is not required nor sufficient for Par complex assembly in the presence of Par6. This model rationalizes the slow assembly observed in Figure 2C by increasing the local concentration of Par6 through clustering, aPKC is able to bind and open Par6, hence reducing C-terminal binding to the N terminus and thereby allowing Par6 to bind to Par3 (see section “rationale of Par complex assembly pathway” in STAR Methods for further details).

### An asymmetric cortex of the Par complex is sufficient to induce spindle orientation

**Figure 4:**
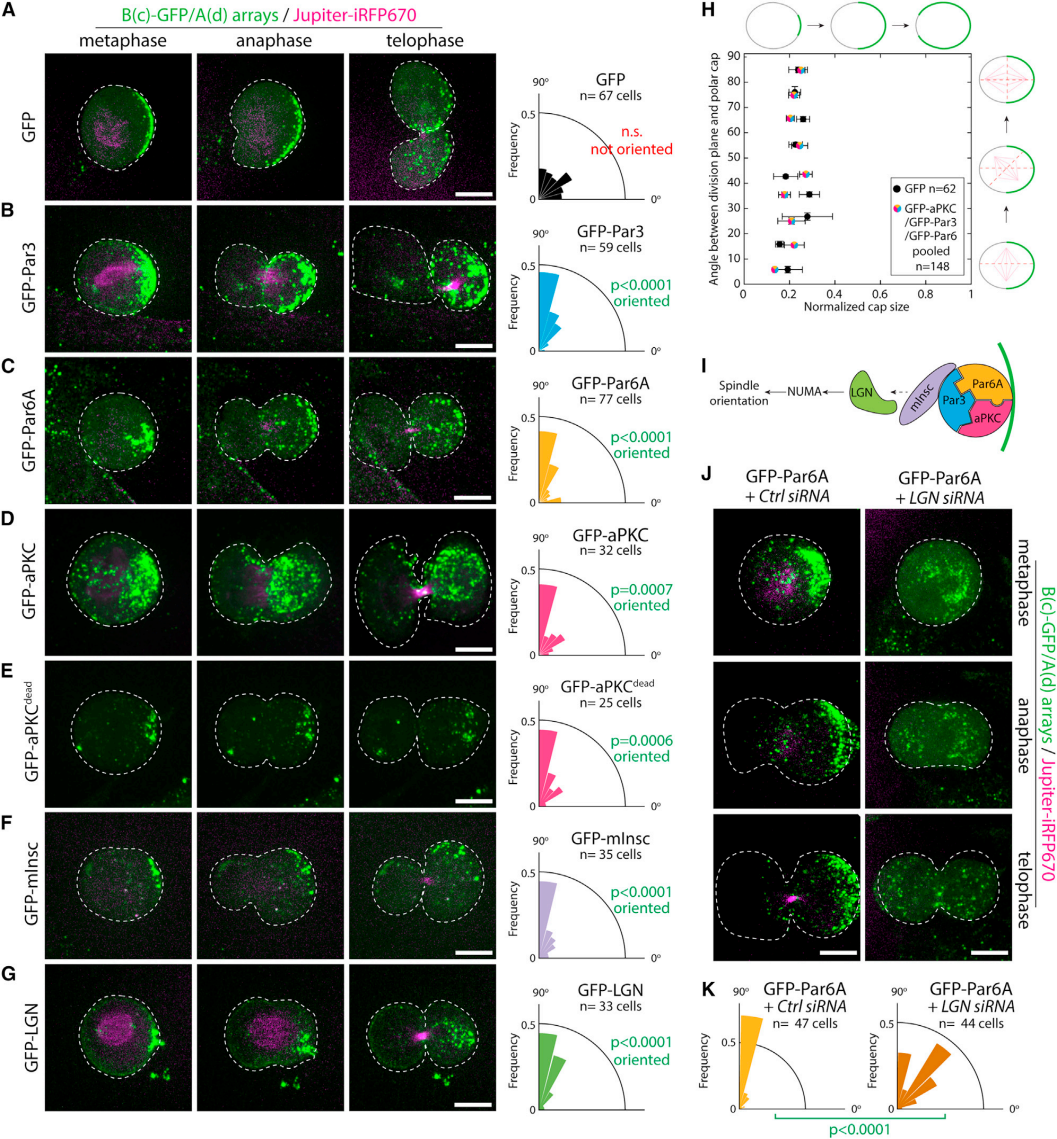
An asymmetric Par complex cortex is sufficient to orient the mitotic spindle (A–G) Left: arrays were assembled on 3T3 cells stably co-expressing Jupiter-iRFP670, GBP-TM-GBP, and indicated GFP-fusions. Cells were stalled in mitosis for >12 h then imaged by SDCM upon release of the nocodazole block. Right: angle between the division plane and the cap in indicated conditions (90°: perfect alignment between cap and spindle). Statistics: unpaired Wilcoxon signed-rank test considering a 45° angle. (H) Angle between the division plane and the cap as a function of the cap size. aPKC, Par6A, and Par3 datasets were pooled (see Figure S8 for individual plots). (I) Simplified spindle orientation pathway.^1^ (J) Dividing cells with Par6A caps depleted for LGN (or treated with control siRNA) were processed and imaged as above to determine the angle between the cap and the division plane. (K) Orientation of the division for the cells treated as in (J) (statistics: Mann Whitney U test). All panels correspond to MIPs. Scale bars, 5 μm. See also Figure S8.

We next investigated whether Par complex caps were sufficient to drive key processes of asymmetric cell division in mammals. In this paper, we consider asymmetric cell division as a division displaying physical asymmetries, for instance, with respect to the spindle and cortical caps, rather than asymmetry in cell fate. At first, we focused on the orientation of cell division (Figure 4). In unpolarized cells like NIH/3T3, the Par complex does not form caps during mitosis, and therefore the division is symmetric.

Importantly, asymmetric caps of GFP did not affect the orientation of division (Figure 4A; Video S6), with the division angle being uncorrelated with either the position or the size of the caps (Figure 4H; Figures S8A and S8B for individual data points). This demonstrates that our synthetic caps do not per se affect the division orientation. In stark contrast, targeting any Par complex subunit to the cap robustly oriented division (Figures 4B–4D; Videos S7, S8, and S9; see also Figure S8C for verification that the Par complexes assembled upon clustering remain assembled when array form caps in dividing cells). aPKC^dead^ caps were as potent as their wild-type counterparts at inducing spindle orientation, indicating that the kinase activity of aPKC is dispensable for spindle orientation (Figures 4D and 4E; Video S8). Due to the preferential orientation of the spindle perpendicular to the caps, Par complex caps were also sufficient to induce another defining feature of asymmetric cell division, namely the preferential inheritance of polar caps by only one daughter cell (Figures 4B–4E; Videos S7, S8, S9, and S10). These results establish that an asymmetric cortex of the Par complex is sufficient to induce spindle orientation in unpolarized mammalian cells.

We subsequently wondered if asymmetric caps of other proteins known to be *required* for spindle orientation were also *sufficient* to do so in mammalian cells. In particular, Inscutable (mInsc) and LGN (also known as Gpsm2) are well established to act downstream of Par3 to orient the mitotic spindle by pulling on astral microtubules via dynein^1^ (Figure 4I). We found that caps of mInsc and LGN robustly oriented the spindle and thereby led to asymmetric inheritance of the polar cap (Figures 4F and 4G; Videos S11 and S12). Our findings that LGN caps are sufficient to reconstitute spindle orientation in mammalian cells confirm the findings from di Pietro and colleagues using cell-cell junctions to reconstitute polarity.^13^ Importantly, Par complex-induced spindle orientation in our assay indeed occurs via LGN, as LGN depletion significantly impaired the ability of Par6 caps to induce spindle orientation (Figures 4J and 4K).

Interestingly, we found no obvious correlation between cap size and the robustness of spindle orientation upon targeting Par complex components or mInsc/LGN to the cap (Figures 4H, S8A, S8D, and S8E for individual data points). This suggests that the molecular composition of the cap, rather than its size, is the determining factor for spindle orientation.

### An asymmetric cortex of the aPKC and Par3 is sufficient to induce central spindle asymmetry

**Figure 5:**
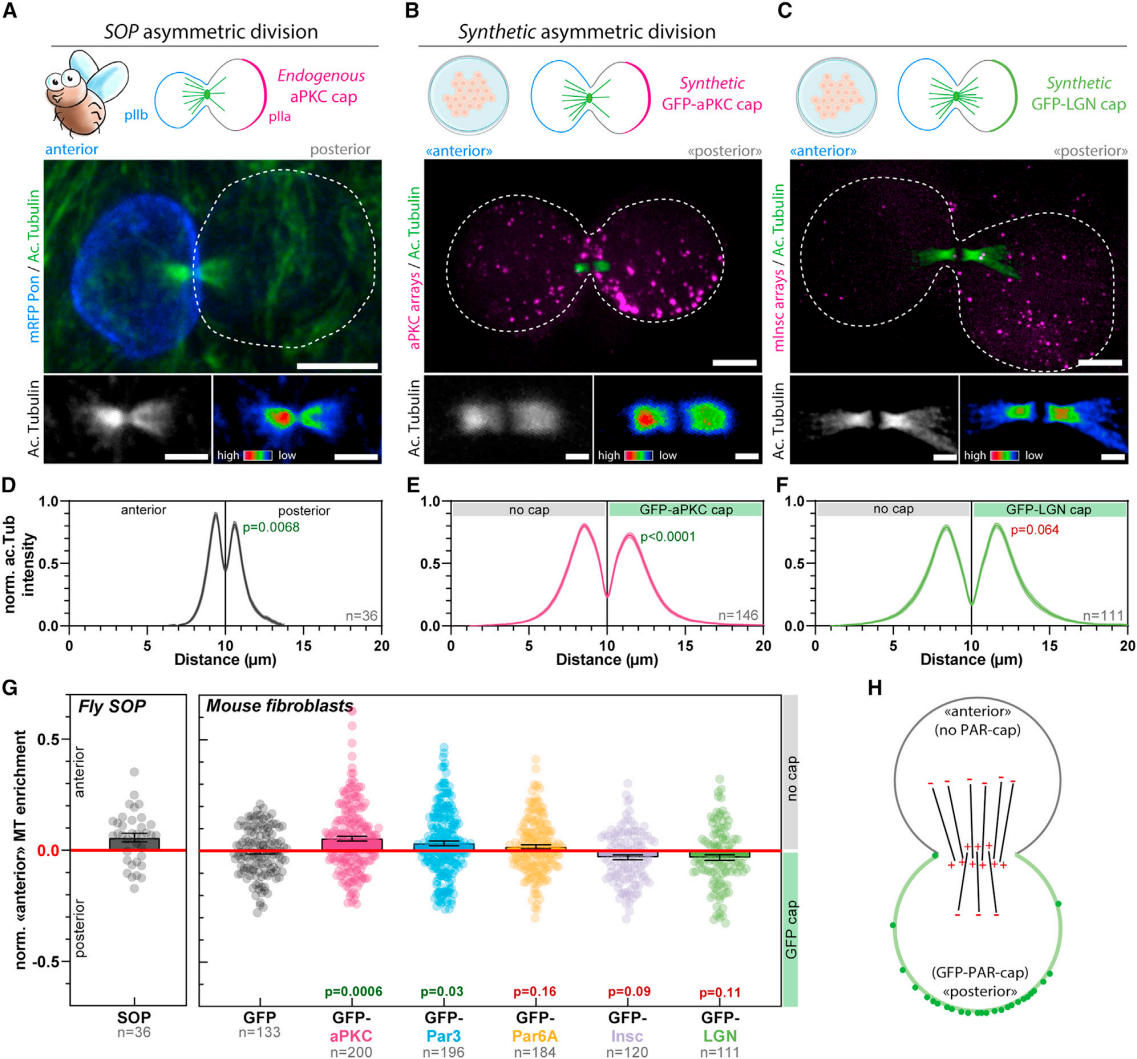
Asymmetric cortical aPKC controls central spindle symmetry breaking (A) SOP cell expressing mRFP-Pon as a marker of the anterior pIIb cell (the cell not inheriting the core Par complex cap) were fixed and immunostained for acetylated tubulin. Bottom: acetyl-tubulin channel with a grayscale (left) or rainbow (right) lookup table (LUT). (B and C) Arrays were assembled on 3T3 cells stably expressing GBP-TM-GBP and indicated construct 12 h before mitotic shake-off, then stained for acetyl-tubulin. We define the side without the cap as the “anterior” and the side with the GFP-cap as the “posterior,” in respect to the situation in fly SOP cells, where the aPKC cap is present on the posterior side. (D–F) Acetyl-tubulin intensity pseudo-linescan through the central spindle (mean ± SEM see STAR Methods) in indicated samples. Statistics: paired t test between the respective peak values in each cell. (G) Normalized enrichment of microtubule density on the anterior side (no cap) of the central spindle as a function of the protein targeted to the cap. Positive values correspond to a higher microtubule density on the anterior side (no cap), while negative values indicate an increased density on the posterior side (GFP-cap). Statistics: Mann-Whitney U test compared with GFP. (H) Summary: synthetic Par-complex caps are sufficient to promote central spindle symmetry breaking, with lower microtubule densities on the side of the Parcap and higher microtubule densities on the side opposite to the cap. All panels correspond to MIPs. Scale bars: 5 μm (A–C, top) and 1 μm (A–C, bottom). See also Figure S9.

Given the ability of Par complex caps to induce mitotic spindle orientation, we next investigated if such caps were sufficient to reconstitute other aspects of asymmetric cell division. During the asymmetric division of *Drosophila* sensory organ precursors (SOPs), the anaphase-specific spindle, known as the central spindle, also undergoes symmetry breaking, whereby the anterior side of the central spindle contains more microtubules than the posterior^5^ (Figures 5A and S9A). This asymmetric central spindle, in turn, orchestrates the polarized trafficking of signaling endosomes containing Notch and its ligand Delta toward only one daughter cell, thereby contributing to asymmetric cell fate determination.^5^ Central spindle asymmetry in SOPs is abolished upon overexpression of lgl3A,^5^ a non-phosphorylatable version of Lethal (2) giant larvae (lgl), a key target of aPKC involved in polarity establishment.^19^ This indicates that lgl phosphorylation and/or Par complex polarity are required for central spindle asymmetry since aPKC polarity is partially affected by lgl3A overexpression.^29^ Here, we extended this to conclusively demonstrate that Par complex polarity is required, in flies, for central spindle asymmetry. Central spindle asymmetry was abolished upon fly Par3 (Baz) depletion in *Drosophila* SOPs (Figures S9B and S9C). We thus wondered if this Par-controlled phenomenon is conserved in mammals and whether we could reconstitute it in our division assay.

Remarkably, upon reconstitution of an asymmetric cap of aPKC in unpolarized 3T3 cells, the central spindle became asymmetric, like in SOPs (Figures 5B and 5E for pseudo-linescan through the spindle; see STAR Methods). Importantly, the orientation was also conserved between species: the side that inherits the Par complex cap containing aPKC is the one that displays a lower microtubule density (Figures 5A, 5B, 5D, 5E, 5G, and 5H). Strikingly, although central spindle asymmetry also occurred upon polar targeting of the other core Par complex component Par3, albeit to a lesser extent as compared to targeting of aPKC, it did not occur with Par6 (Figures 5G, S9D–S9F, and S9H–S9J). We observed a slight bias toward a microtubule enrichment on the side without the GFP-Par6 cap, but this was not significant compared with the GFP control (Figure 5G). Importantly, similar asymmetry levels were observed upon selecting mitotic cells either using mitotic shake-off (Figure 5G) or by stalling them in mitosis using nocodazole (Figure S9L), suggesting that the time spent in metaphase is not relevant for the central spindle symmetry breaking process. Altogether, these data establish that reconstituting an asymmetric cap of aPKC and Par3 is sufficient to induce central spindle symmetry breaking in unpolarized mammalian cells.

Interestingly, although polar targeting of mInsc and LGN robustly oriented the spindle along the established polarity axis (Figures 4F and 4G), this did not promote the formation of an asymmetric central spindle (Figures 5C, 5F, 5G, S9G, S9K, and S9L). To confirm that these downstream effectors of the spindle orientation pathway are not involved in central spindle symmetry breaking, we depleted LGN in 3T3 cells with a GFP-aPKC cap. Importantly, LGN depletion did not affect central spindle asymmetry in cells that had, by chance, a properly oriented spindle (Figure S9M). Hence, although LGN promotes spindle orientation, it is neither sufficient nor required for central spindle symmetry breaking. Altogether, these results establish that central spindle asymmetry and spindle orientation are two discrete outputs of Par complex activity that can be untangled.

The fact that mInsc caps do not induce central spindle asymmetry is surprising since mInsc binds to both Par3 and LGN,^34^ at least when clustered. One would therefore expect that mInsc clustering would not only induce spindle orientation (via LGN clustering) but also central spindle asymmetry via Par complex assembly (through Par3 clustering). This provided a unique opportunity to probe the molecular details of the central spindle asymmetry pathway. Strikingly, although mInsc clusters recruited endogenous Par3, these mInsc-Par3 clusters did not recruit aPKC (Figures S9N and S9O). Given our framework for Par complex assembly (Figure 3E), this suggests that the indirect clustering of Par3 via mInsc is not efficient at “opening” Par6 to quantitatively recruit aPKC. This finding pointed toward aPKC being the key effector for central spindle asymmetry, which was in line with aPKC caps being the most potent at inducing central spindle symmetry breaking (Figure 5G).

### Asymmetric cortical aPKC activity induces central spindle asymmetry

**Figure 6:**
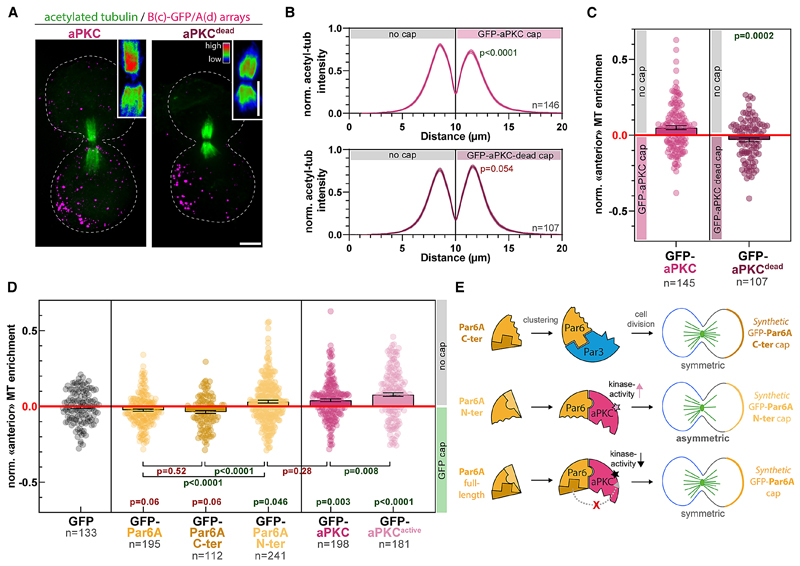
Asymmetric aPKC kinase activity promotes central spindle symmetry breaking (A) 3T3 cells stably expressing GBP-TM-GBP and GFP-aPKC, or its kinase dead variant were processed for array formation 12 h before mitotic shake-off, then stained for acetyl-tubulin. Insert: acetyl-tubulin channel with a rainbow LUT. (B) Acetyl-tubulin intensity pseudo-linescan through the central spindle (mean ± SEM) of cells treated as in (A). Statistics: paired t test between the respective peak values in each cell. GFP-aPKC pseudo-linescan is reproduced from Figure 5B for convenience. (C) Normalized enrichment of microtubule density on the side opposite to the GFP-aPKC cap in cells treated as in (A). (D) 3T3 cells stably expressing GBP-TM-GBP and indicated GFP fusion were processed as in (A), then the normalized enrichment of microtubule density on the side opposite to the cap was measured. Statistics: Mann-Whitney U test, compared with the GFP control or as indicated by lines. p values for Par6 mutants calculated by Kruskal-Wallis test: GFP-Par6A/GFP-Par6A^Cter^: p > 0.99; GFP-Par6A/GFP-Par6A^Nter^: p < 0.0001; GFP-Par6A^Cter^/GFP-Par6A^Nter^: p = 00001 (E) Summary: Par6A^Cter^ caps, which do recruit Par3 but not aPKC, do not lead to the formation of an asymmetric central spindle, given the absence of aPKC. Conversely, Par6A^Nter^ caps induce central spindle asymmetry, unlike Par6A full-length, in line with the proposed ability of Par6^Cter^ to inhibit aPKC’s kinase activity (STAR). All panels correspond to MIPs. Scale bars, 5 μm. See also Table 2 for construct description.

Given that aPKC is markedly more potent at inducing central spindle symmetry breaking up to SOP levels (Figure 5G), we hypothesized that aPKC was the Par complex subunit initiating the process. In line with this prediction, while aPKC^dead^ was competent to induce Par complex assembly (Figure S5) and spindle orientation (Figure 4F), it failed to induce central spindle symmetry breaking (Figures 6A–6C). This establishes that central spindle asymmetry is controlled by an asymmetric cortex of the kinase activity of aPKC.

The fact that central spindle asymmetry depends on aPKC and not the other core Par complex subunits likely explains the different asymmetry levels observed upon clustering of these proteins (Figure 5G). Specifically, although Par3 clustering promotes aPKC recruitment, the level of colocalization amounts to approximately 30% (Figure S6D), implying that a majority of Par3 clusters are lacking aPKC. Thus, cortical aPKC levels are expected to be markedly higher by directly targeting aPKC to the cap compared with indirectly via Par3 (Figure S8C), therefore leading to lower central spindle asymmetry levels, which we observed experimentally (Figure 5G). On the other hand, Par6 caps did not induce central spindle asymmetry (Figure 5G), even though Par6 clustering recruits aPKC more efficiently than Par3 clustering (Figure 2C versus Figure S6C). But importantly, Par6 has been proposed to inhibit the kinase activity of aPKC in the absence of Cdc42.^19^ Thus, although Par6 clustering efficiently clusters aPKC, it would also inhibit its kinase activity, leading to the formation of predominantly symmetric central spindles, as observed. Since this inhibitory effect is thought to be mediated by the C-terminal domain of Par6,^35^ we could test this hypothesis by measuring central spindle asymmetry upon clustering Par6 truncations. Firstly, Par6^Cter^ clusters, which recruit Par3 but not aPKC (Figure 3B), did not promote the formation of asymmetric central spindles, similar to full-length Par6, which does efficiently recruit aPKC (Figures 6D and 6E). Conversely, Par6^Nter^ clustering, which recruits aPKC but not Par3 (Figure 3B), and lacks the aPKC-inhibitory C-terminal domain, induced asymmetric central spindle formation, similar to aPKC clustering (Figures 6D and 6E). Furthermore, if full-length Par6 does indeed inhibit aPKC and this is the reason for the formation of predominantly symmetric central spindles upon Par6 clustering, then central spindle symmetry breaking should be rather sensitive to the levels of kinase activity of aPKC. As predicted, clustering a kinase-hyperactive mutant of aPKC, which does not affect Par complex assembly (Figure S5), resulted in significantly higher levels of central spindle asymmetry as compared with wild-type aPKC (Figure 6D). Together, these results demonstrate that central spindle asymmetry depends on the kinase activity of aPKC, which likely can be inhibited by the C-terminal domain of Par6 (Figure 6E). Thus, the fact that we observe asymmetric central spindles upon clustering of aPKC, even though endogenous Par6 is present, most likely comes from the fact that not all clusters efficiently recruit Par6 (Figure S4I).

### Central spindle symmetry breaking is controlled by CAMSAP3, Kif2A, Kif2B, and Kif2C

We then set out to determine the molecular mechanism of central spindle asymmetry downstream of aPKC. In flies, the microtubule stabilizer Patronin and the microtubule depolymerizing kinesin-13 Klp10A are involved in central spindle symmetry breaking.^5^ In mammals, there are three Patronin homologs, Camsap1-3,^36^ and three Klp10A homologs, Kif2A, Kif2B, and Kif2C/MCAK.^37^

**Figure 7:**
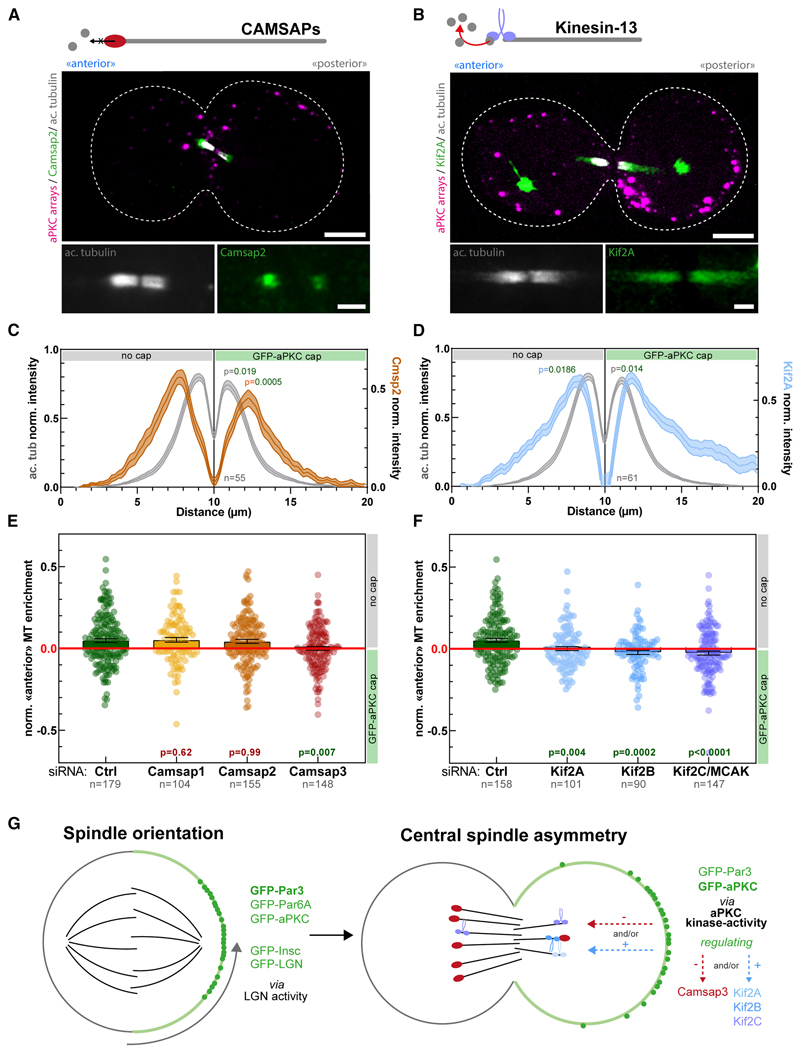
Camsap3, Kif2A, Kif2B, and Kif2C promote central spindle symmetry breaking (A and B) Dividing 3T3 cells with a synthetic GFP-aPKC cap immunostained for acetyl-tubulin and Camsap2 (A) or Kif2A (B). (C and D) Pseudo-linescan through the central spindle in cells treated as in (A) and (B) (mean ± SEM). Left y axis corresponds to acetyl-tubulin and right y axis corresponds to Camsap2 (C) or Kif2A (D) signals. Statistics: paired t test between the respective peak values in each cell. (E and F) 3T3 cells stably expressing GBP-TM-GBP and GFP-aPKC were treated with the indicated siRNA before array formation 12 h before mitotic shake-off and stained for acetyl-tubulin. Then the normalized enrichment of microtubule density on the side opposite to the aPKC-cap was measured. Statistics: Mann-Whitney U test compared with respective control siRNA. (G) Summary of the results presented in Figures 4, 5, and 6, as well as this figure. Left: caps of GFP-aPKC, GFP-Par6A, GFP-Par3, GFP-Insc, and GFP-LGN are sufficient to orient the spindle along the *de novo* established polarity axis. Spindle orientation requires LGN. Right: When cells with aPKC and Par3 caps proceed through cell division, they form an asymmetric central spindle, which depends on aPKC’s kinase activity. Central spindle asymmetry requires Camsap3, Kif2A, Kif2B, and Kif2C, which may thus be aPKC targets. All panels correspond to MIPs. Scale bars: 5 μm (A and B, top) and 2 μm (A and B, bottom). See also Figure S10.

We first investigated the subcellular localization of Camsap2 and Kif2A on asymmetric central spindles induced by aPKC-caps (Figures 7A and 7B). Interestingly, Camsap2 displayed an asymmetric distribution that matched that of microtubule densities, with a higher density on the side without the cap, and a lower density on the side with the aPKC-cap (Figures 7A and 7C). On the other hand, Kif2A also displayed an asymmetric localization, but in this case opposing the asymmetry of microtubule densities (Figures 7B and 7D), with a lower density on the side without the cap and a higher density on the other side. Note that Camsap2 and Kif2A signals are shifted toward the edges with respect to acetylated microtubules, which is in line with previous findings in flies^5^ and attributed to the well-established affinity of Camsap/Patronin for microtubule minus ends^36,38^ that are enriched on the edges of the central spindle.^39^

To probe an eventual role of Camsap and Kinesins-13s in central spindle symmetry breaking, we treated cells forming aPKC-caps with small interfering RNA (siRNA) targeting these proteins. Importantly, although Camsap1 and Camsap2 depletion did not significantly affect central spindle asymmetry, Camsap3 depletion resulted in the formation of symmetric central spindles (Figure 7E; see also Figures S10A, S10C, and S10E for pseudo-line-scans, Camsap2 depletion control, and individual Camsap3 siRNAs). Hence, out of all Patronin homologs, only Camsap3 appears to have inherited the ancestral function of promoting central spindle asymmetry. By contrast, depletion of all kinesin-13 homologs Kif2A, Kif2B, or Kif2C/MCAK abolished central spindle asymmetry (Figure 7F; see also Figure S10B for pseudo-line-scans; Figure S10D for Kif2A depletion controls; and Figures S10F–S10H for individual Kif2A/B/C siRNAs). Altogether, these data suggest that the molecular mechanism of Par complex induced central spindle symmetry breaking is conserved from flies to mammals, with Camsap3 and Kif2A/Kif2B/MCAK having inherited the ancestral function of Patronin and Klp10A, respectively.

## Discussion

### Synthetic dissection of the input/output logic of polarity pathways

Here, we engineered a population of mammalian cells that spontaneously polarize a given protein of interest at their cortex just prior to division. Importantly, since cap formation with our method is an intrinsic property of our *de novo* designed polymer, it is possible to adapt our method to other cell types/species, as we did for U2OS cells, mouse ES cells, and *Drosophila* S2 cells (Figure S2). It must be emphasized that the reconstitution of cortical polarity in cultured cells *de facto* enables live cell imaging at high spatiotemporal resolution. This is a key advantage over tissues and organoids, which have until now been required to study Par complex polarity in mammals but where quantitative imaging is more challenging because of scattering. We envision that this key advantage of our method will be harnessed in the future to provide unprecedented details of the dynamics of symmetry breaking using subcellular light sheet microscopy^40,41^ or of the structure of polarized cytoskeleton networks using cryo-electron tomography,^42^ both methods being readily applicable to cultured cells. Finally, given the generality of our method and its applicability to stem cells, we anticipate that it will prove useful to decipher the input/output logic of other polarity pathways beyond the Par complex.

Our work provides an orthogonal way to reconstitute cortical polarity compared with previous work.^11–15,17^ Specifically, our method combines five characteristics that were not simultaneously found in the same method previously, namely it is (1) inducible, (2) fast, (3) high-throughput, (4) it works at the single cell level during division, and (5) the molecular composition of the cap can be controlled. This key combination allows one to dissect the downstream effects of polarity proteins once assembled into a cap (i.e., the pathway outputs), but it also allows the study of the assembly of polarity signaling networks at the cortex (i.e., its inputs). We could capitalize on this new method to systematically and quantitatively dissect the molecular assembly routes of the Par complex (Figures 2 and 3) and consequentially study the effects of a *preassembled* Par complex cap on cyto-skeleton symmetry breaking and identify the molecular mechanism involved (Figures 4, 5, 6, and 7).

### A unified framework for Par complex assembly

Integrating data from millions of arrays, we could shed new light on the assembly sequence of the Par complex pathway and propose a unified paradigm for this process (Figure 3G). Importantly, our results are highly consistent with previous work. In particular, we have confirmed that: (1) Par6 and aPKC can interact, with aPKC^E85/R91^ and Par6^K19^ vital for this interaction,^26^ (2) Par6 and Par3 can interact, with Par3^G600,602^ and Par6^121-257^ vital for this interaction,^43^ (3) clustering/oligomerization of Par3 is required for Par complex assembly,^30^ (4) the Par complex can assemble into clusters at cell-cell junctions,^44^ and (5) Par complex assembly can occur despite aPKC phosphorylation of Par3.^31^

Our work extends these previous findings and provides novel insights into Par complex biology. In particular, we found that: (1) clustering of any core Par complex component can induce assembly of the tripartite complex (Figures 2, S5, and S6), (2) Par complex assembly takes time, likely due to Par6 autoinhibition (Figures 3 and S7), (3) aPKC kinase activity is dispensable for Par complex assembly (Figure S5), and (4) even if aPKC and Par3 can interact directly, Par6 is crucial for Par3 to effectively associate with aPKC (Figures 2, S5, and S6). This last finding is somewhat at odds with the fact that a V606A mutant of aPKC, abolishing Par3 binding, was found unable to be recruited to endogenous Par3 caps in flies, ^31^ but this could be explained with Par6 being limiting in these cells in light of our results (see section “rationale of Par complex assembly pathway” in STAR Methods for further details).

### Par complex polarity has two discrete effects on cytoskeleton symmetry breaking

A major finding of this work is that an asymmetric cortex of the Par complex has two discrete, independent outputs during mitosis, promoting “polarized” cell division of normally unpolarized cells (Figure 7G). On the one hand, inducing polar caps of any Par complex subunits (Par3, Par6A, or aPKC), as well as the downstream effectors mInsc and LGN, is sufficient to induce spindle orientation along the polarity axis (Figure 7G, left panel). On the other hand, inducing caps of Par3 and aPKC, but not mInsc or LGN, induces central spindle symmetry breaking (Figure 7G, right panel). Importantly, Par-mediated spindle orientation requires LGN (Figure 4J), while it is dispensable for central spindle asymmetry (Figure S9M). Conversely, central spindle symmetry breaking depends on aPKC’s kinase activity (Figure 6), which is dispensable for spindle orientation (Figure 4E). In other words, while spindle orientation and central spindle asymmetry occur downstream of the Par complex, they occur via two different molecular cascades that can be untangled: spindle orientation occurs via Par3/mInsc/LGN, while central spindle asymmetry occurs via the kinase activity of aPKC. Furthermore, we found that fly Par3 (Bazooka) is required for central spindle asymmetry in fly SOPs (Figures S9A–S9C). Since SOP cells lack mInsc and thus do not orient the spindle via the Par complex,^45^ spindle orientation and central spindle asymmetry can also be untangled *in vivo* in flies. Note that this also rationalizes why some loss-of-function mutants were found to affect central spindle asymmetry without affecting spindle orientation both in flies^5,10^ and in mammals (Figures 4 and 7). It is also worth noting that, in light of our assembly results, these two activities are bridged by Par6 within the Par complex, as Par6 is absolutely required for aPKC to bind to Par3. Par6 could thus regulate the coupling between the two outputs of the Par complex. This is in line with the ability of the Par6 C-terminal domain to modulate aPKC’s kinase activity^19^ and therefore tune central spindle asymmetry (Figure 6).

Our data also suggests a paradigm to explain central spindle symmetry breaking. Indeed, although it is hard to envision that a gradient of microtubule regulators could exist in cells due to the highly diffusive nature of the cytosol, our finding that central spindle asymmetry is controlled by cortical aPKC activity (Figure 6) could suggest the existence of an activity gradient. Indeed, if the activity of the microtubule regulator(s) responsible for central spindle asymmetry was regulated by aPKC-mediated phosphorylation, then an asymmetric aPKC cortex could lead to a steady-state gradient of the phosphorylated regulator in the presence of cytosolic phosphatases. Hence, an activity gradient could exist despite diffusion. A fascinating question for future research will be to identify the key target(s) of aPKC that controls the information flow between the cortex and the cytosol. Our data suggests that the microtubule regulators Camsap3, Kif2A, Kif2B, and Kif2C/MCAK, could be such downstream effectors of aPKC (Figure 7). Since Camsap3 protects microtubules from depolymerization,^36,46^ it may be negatively regulated by aPKC’s kinase activity, either directly or indirectly, and consequently, Camsap3 may be more active on the side opposite to the cap, resulting in a higher microtubule density (Figure 7G, right panel). Conversely, the microtubule destabilizing kinesins, Kif2A, Kif2B, and Kif2C/MCAK, could be positively regulated by aPKC, leading to increased activity on the side of the cap, and, hence, a lower microtubule density on this side of the central spindle (Figure 7G, right panel).

In conclusion, this work highlights the power of synthetic biology and protein design to address cell biology questions in an orthogonal manner. Specifically, we could demonstrate that central spindle asymmetry is a conserved hallmark of asymmetric division, and we envision that our method will enable further delineation of the molecular mechanisms governing intrinsic cell polarity and cytoskeleton symmetry breaking in various species. In particular, it will be interesting to know if the reconstitution of Par complex caps is sufficient to induce other aspects of asymmetric cell division in fibroblasts, such as asymmetric segregation of cell fate determinants,^1^ asymmetric gene expression, or polarized trafficking of endosomes,^47^ mitochondria,^8^ and/or lysosomes.^7^

### Limitations of the study

Although the method described here has several advantages to investigate the assembly and downstream effects of polarity pathways, it is not adapted to answer all polarity questions. In particular, our designed arrays only form caps in rounded-up cells, and thus our method cannot be used to reconstitute polarity in spread interphase cells, contrary to previous methods.^11–15,17^ Similarly, our method cannot reconstitute polarity in tissues, as it is specifically designed to reconstitute polarity in *single* cells, contrary to other methods based on cell-cell contacts for instance.^12,13^ Last, array formation with our method is irreversible. Thus, although our method can help investigate the assembly of polarity cues, it cannot investigate their disassembly, contrary to methods based on optogenetics.^15^ To help the reader identify the method most suited to address their biological question, we provide a table highlighting the pros and cons of each method (Table 1).

## Star ⋆ Methods

Detailed methods are provided in the online version of this paper and include the following:

### Key Resources Table

**Table:** 

Reagent or Resource	Source	Identifier
Antibodies		
Rabbit polyclonal anti-Par3	Merck	07-330; RRID:AB_2101325
Mouse monoclonal anti-aPKC	Santa Cruz	sc-17837; RRID:AB_2172068
Mouse polyclonal anti-*α* K40 acetylated tubulin	HPA cultures	C3B9
Mouse monoclonal anti-PC	Roche	HPC4
Rabbit polyclonal anti-GST	Abcam	ab19256; RRID:AB_444809
Rat monoclonal anti-HA	Roche	3F10; RRID:AB_2314622
Goat polyclonal anti-rabbit Alexa-555	Thermo	A32732; RRID:AB_2633281
Goat polyclonal anti-rabbit Alexa-647	Thermo	A21246; RRID:AB_2535814
Goat polyclonal anti-mouse Alexa-647	Thermo	A-21235; RRID: AB_2535804
Donkey polyclonal anti-rat Cy3	Jackson ImmunoResearch	AB_2340667
Rabbit polyclonal anti-Camsap2	Proteinbiotech	17880-1-AP; RRID:AB_2068826
rabbit polyclonal anti-Kif2A	Novus Biologicals	NB500-180; RRID:AB_10002348
Chemicals, peptides, and recombinant proteins		
DMEM + Glutamax	Gibco	10566016
Penicillin-Streptomycin	Gibco	15140122
DMEM	ThermoFisher Scientific	41966
MEM	ThermoFisher Scientific	11140035
DMEM F12	ThermoFisher Scientific	21041025
Glutamax	ThermoFisher Scientific	35050061
sodium pyruvate	ThermoFisher Scientific	11360070
Neurobasal A	ThermoFisher Scientific	12348017
B27	ThermoFisher Scientific	10889-038
β-mercaptoethanol	ThermoFisher Scientific	31350-010
Hygromycin B Gold	InvivoGen	Ant-hg-1
puromycin	ThermoFisher Scientific	A11138-03
Foetal Bovine Serum	Gibco	10270106
Donor Bovine Serum	Gibco	16030074
Lipofectamine 3000	Invitrogen	L3000001
MEK inhibitor	Cambridge Stem Cell Institute	PD0325901
GSK3 inhibitor	Cambridge Stem Cell Institute	CHIR99021
Leukaemia Inhibitory Factor	Marko Hyvonen’s lab	https://hyvonen.bioc.cam.ac.uk/proteins/mLIF_datasheet.pdf
MycoAlert Mycoplasma Detection kit	Lonza	LT07-118
Schneider Medium	ThermoFisher Scientific	21720024
Insect Express medium	Lonza	BELN12-730Q
Effectene	Qiagen	301425
Pluronic F-68	Gibco	24040032
A(d)	Ben-Sasson et al.^20^	N/A
A(d)-mCherry	Ben-Sasson et al.^20^	N/A
B(c)-GFP	Ben-Sasson et al.^20^	N/A
B(c)-SpyCatcher	This study	N/A
SpyTag-DLL4	Watson et al.^48^	N/A
Fibronectin	Sigma	F1141
GFP protein	This study	N/A
Fipper-TR	Spirochrome	SC020
Concanavalin A	Sigma	L7647-25MG
**Experimental model: Cell lines**		
NiH/3T3 Flp-In cells (3T3 cells)	Invitrogen	R76107
Stable 3T3 cells expressing GG-GFP*	This study	N/A
Stable 3T3 cells expressing GG-Par3(180kD)*	This study	N/A
Stable 3T3 cells expressing GG-Par3(100kD)*	This study	N/A
Stable 3T3 cells expressing GG-Par3(83-1319)*	This study	N/A
Stable 3T3 cells expressing GG-Par3(S824A/S826A)*	This study	N/A
Stable 3T3 cells expressing GG-Par3(S824D/S826D)*	This study	N/A
Stable 3T3 cells expressing GG-Par3(100kD, Δ1-83)*	This study	N/A
Stable 3T3 cells expressing GG-Par3(100kD Δ1-83 Δ728-813)*	This study	N/A
Stable 3T3 cells expressing GG-Par3(100kD G600A/G602A Δ1-83 Δ728-813)*	This study	N/A
Stable 3T3 cells expressing GG- Par6A*	This study	N/A
Stable 3T3 cells expressing GG- Par6B*	This study	N/A
Stable 3T3 cells expressing GG- Par6G*	This study	N/A
Stable 3T3 cells expressing GG-Par6A(K19A)*	This study	N/A
Stable 3T3 cells expressing GG-Par6A(1-121)*	This study	N/A
Stable 3T3 cells expressing GG-Par6A(121-346)*	This study	N/A
Stable 3T3 cells expressing GG-Par6A(247-346)*	This study	N/A
Stable 3T3 cells expressing GG-PAR6A(121-346, LGF169-171AAA)*	This study	N/A
Stable 3T3 cells expressing GG-Jo-Par6A-In*	This study	N/A
Stable 3T3 cells expressing GG-aPKCi*	This study	N/A
Stable 3T3 cells expressing GG-aPKC_ι_(E85A/R91A)*	This study	N/A
Stable 3T3 cells expressing GG-aPKC_ι_(K274W)*	This study	N/A
Stable 3T3 cells expressing GG-aPKCr_l_(A129E)*	This study	N/A
Stable 3T3 cells expressing GG-aPKC_ι_(E85A/R91A/K274W)*	This study	N/A
Stable 3T3 cells expressing GG-aPKC_ι_(E85A/R91A/A129E)*	This study	N/A
Stable 3T3 cells expressing GG-aPKC_ι_(Δ857-862)*	This study	N/A
Stable 3T3 cells expressing GG-mInsc*	This study	N/A
Stable 3T3 cells expressing GG-LGN*	This study	N/A
Stable 3T3 cells expressing His-PC-GBP-TM-VSVG-Par6A	This study	N/A
Stable 3T3 cells expressing TetOn** His-PC-GBP-TM-VSVG-mScarlet	This study	N/A
Stable 3T3 cells expressing TetOn** His-PC-GBP-TM-VSVG-aPKCi	This study	N/A
Stable 3T3 cells expressing TetOn** His-PC-GBP-TM-VSVG-Par3(180kD)	This study	N/A
Stable 3T3 cells expressing TetOn** His-PC-GBP-TM-VSVG-Par3(100kD)	This study	N/A
Stable 3T3 cells expressing TetOn** His-PC-GBP-TM-VSVG-mInsc	This study	N/A
Stable 3T3 cells expressing TetOn** His-PC-GBP-TM-VSVG-Par3(S824A/S826A)	This study	N/A
Stable 3T3 cells expressing TetOn** His-PC-GBP-TM-VSVG-Par3(S824D/S826D)	This study	N/A
E14WT Mouse ESCs	Jenny Nichols	N/A
Drosophila S2 Cells	ThermoFisher Scientific	R69007
U2OS	ATCC	HTB-96
Stable U2OS cells expressing FLAG-Notch1-EGFP	Malecki et al.^49^	N/A
**Experimental model: Drosophila Melanogaster stocks**		
*w^1118^*	Bloomington	#3605
*Neur>Gal4*	Bellaïche et al.^50^	N/A
*UAS>mRFP-Pon^lD^*	Emery et al.^51^	N/A
*Baz-mScarlet* knock-in	Houssin et al.^52^	N/A
*Jupiter-GFP* knock-in	Bloomington	#6836
**Software and algorithms**
Fiji	Schindelin et al.^53^	https://imagej.net/Fiji
MATLAB 2020b	Mathworks	https://uk.mathworks.com/products/matlab.html
Adobe Premiere 27.3.1	ADOBE	https://www.adobe.com/
GPU Drift correction	Planelles-Herrero et al.^10^	https://github.com/deriverylab/GPU_registration
Wavelet “à trous” GPU denoising	Planelles-Herrero et al.^10^	https://github.com/deriverylab / GPU_wavelet_a_trous
3D array colocalization	This study	https://github.com/deriverylab/3D_array_colocalization; https://doi.org/10.5281/zenodo.8357466
CudaDecon	Janelia Research Campus	https://github.com/scopetools/cudaDecon
Lsfmtools	James Manton and Jérôme Boulanger	https://github.com/jdmanton/lsfm_tools
LimeSeg	Machado et al.^54^	https://imagej.net/plugins/limeseg
SymPhoTime 64 2.0	PicoQuant	https://www.picoquant.com
Metamorph 7.10.1.161	Molecular devices	www.moleculardevices.com
Napari	Ahlers et al.^55^	https://napari.org/
**Protein structure predictions**		
Par6A	AlphaFold2	https://alphafold.ebi.ac.uk/entry/Q9NPB6

* GG refers to the tricistronic His-PC-GBP-TM-VSVG-GBP + Flag iRFP670-Jupiter + PC-GFP-[protein of interest]

** TetOn refers to a bicistronic rtTA3 + Tet promoter driving the given ORF

### Resource Availability

#### Lead contact

Further information and requests for resources and reagents should be directed to and will be fulfilled by the lead contact, Emmanuel Derivery (derivery@mrc-lmb.cam.ac.uk).

#### Materials availability

All unique/stable materials generated in this study are available by request from the lead contact.

### Experimental Model And Study Participant Details

#### Cell culture

Flp-In NiH/3T3 cells (Invitrogen) were cultured in DMEM-Glutamax (Gibco) supplemented with 10% Donor Bovine Serum (Gibco) and Pen/Strep 100 units/ml (Gibco) at 37°C with 5% CO_2_. Cells were transfected with Lipofectamine 3000 (Invitrogen). Stable transfectants at the same genomic locus were obtained according to the manufacturer’s instructions by homologous recombination at the FRT site were selected using 100 μg/mL Hygromycin B Gold (Invivogen).

U2OS cells (ATCC) were cultured in DMEM-Glutamax supplemented with 10% fetal bovine serum (Gibco) and 1% Pen/Strep at 37°C with 5% CO_2_ and also transfected with Lipofectamine 3000. U2OS cells expressing FLAG-Notch1-EGFP chimeric receptors were grown as described previously.^49^

Mouse ES cells (E14 wild-type, kind gift of Jenny Nichols, MRC Human Genetics Unit, UK) were cultured in gelatin-coated plates in Fc 2i/LIF medium. 2i/LIF consists of 1 μM MEK inhibitor PD0325901 (Cambridge Stem Cell Institute), 3 μM GSK3 inhibitor CHIR99021 (Cambridge Stem Cell Institute), and 10 ng/ml Leukaemia Inhibitory Factor (LIF, Cambridge Stem Cell Institute), and it is added to the medium to preserve naïve pluripotency. Fc basal medium contains DMEM (Thermo Fisher Scientific), 15% fetal bovine serum (Gibco) penicillin-streptomycin (Gibco), GlutaMAX (Thermo Fisher Scientific), MEM non-essential amino acids (Thermo Fisher Scientific), sodium pyruvate (Thermo Fisher Scientific) and 100 μM β-mercaptoethanol (Thermo Fisher Scientific). Mouse ES cells were routinely passaged with Trypsin-EDTA (produced in-house) at a ratio of 1 to 10. Fc medium was used to neutralize the trypsin and cells were centrifuged at 1000 r.p.m. for 5 min. Cells were cultured at 37 °C in 21% O_2_ and 5% CO_2_, and mycoplasma contamination was routinely screened using the MycoAlert Mycoplasma Detection kit (Lonza).

For expression into ES cells, cells were co-transfected with a plasmid of interest containing the piggyBac transposon sequence (MXS piggyBac GBP-TM-GBP Puro), as well as a plasmid expressing the piggyBac-recombinase using Lipofectamine 3000 Transfection Reagent (Thermo Fisher Scientific) following the manufacturer’s instructions. Two days after transfection cells were selected with 2 mg/mL puromycin (Thermo Fisher Scientific).

S2 cells (Thermo Fisher Scientific mycoplasm-free judged by Dapi staining) were grown at 25°C in Schneider Medium (Thermo Fisher Scientific) supplemented with 1% Pen/Strep (Gibco) and 10 % (vol/vol) Foetal Bovine Serum (Gibco, Heat-inactivated for 1h at 70°C). Stable cell lines were obtained by transfection with pMT puro vectors using Effectene (Qiagen) according to the manufacturer’s instructions, followed by selection in 5 μg/mL Puromycin (Thermo Fisher). Cells were then adapted to liquid culture by gradually adapting them to grow in protein-free Insect Express medium (Lonza) supplemented with 1% vol/vol Pen/Strep (Gibco) and 0.1% vol/vol Pluronic F-68 (Gibco) and in 5 μg/mL Puromycin in a shaking incubator 27°C. This efficiently select for round, non-adherent cells.

Identities of the cell lines were verified by antibiotic resistance markers. All cell lines used in this study are listed in the key resources table.

#### Drosophila stocks

Fly handling was done according to standard procedures. To generate the desired genotypes and remove balancers, all experiments were performed on F1 of crosses at 25°C. Transgenes used in this study included *UAS-mRFP-Pon^LD^* (Emery et al.^51^), *Neur>Gal4* (Bellaïcheet al.^50^), Jupiter-GFP (Morin etal.^56^, knock in at endogenous locus Bloomington #6836), *Baz-mScarlet-I* (knock in at endogenous locus Houssin et al.^52^, kind gift from Jens Januschke), UAS>RFP-RNAi (kind gift from Jens Januschke). For immunofluorescence of dividing SOPS, larvae were then shifted to 16°C until puparium and were shifted to 25°C 16h prior to dissection (or 29°C for RNAi experiments).

Note that although the RFP-RNAi targets both mRFP and mScarlet, since the signal in the red channel is asymmetric in “control #2”, but symmetric in the “Baz depletion” samples in Figure S9B, which only differ by a *wild type* copy of Baz in control#2 (the mRFP-Pon^LD^, mScarlet-Baz and RFP-RNAi are present in both), this suggests that signal in the red channel mostly comes from mRFP-Pon^LD^. This is also coherent with the fact that the signal of mRFP-Pon^LD^ is much stronger than the signal of mScarlet-Baz, and so less visible in our illumination conditions.

#### Detailed genotypes

Figures 5A and 5C: *w*^1118^;*Neur>Gal4, UAS>mRFP-Pon^LD^* / + (25°C)

Figure S9A: *Control #1: w^1118^; UAS>mRFP-Pon^LD^;Neur>Gal4, Jupiter-GFP* / + (25°C)

Figure S9B Control #2: *Baz-mScarlet-I/ Baz; UAS>mRFP-Pon^LD^ /UAS>RFP^RNAi^;Neur>Gal4, Jupiter-GFP / (MKRS) (29°C); Baz depletion: Baz-mScarlet-I/Y; UAS>mRFP-Pon^LD^ /UAS>RFP^RNAi^;Neur>Gal4, Jupiter-GFP / (MKRS)* (29°C)

Figure S9C Control #1+#2: *w^1118^; UAS>mRFP-Pon^LD^;Neur>Gal4, Jupiter-GFP / +* (25°C) or *Baz-mScarlet-I/ Baz; UAS>mRFP-Pon^LD^ /UAS>RFP^RNAi^;Neur>Gal4, Jupiter-GFP/(MKRS)* (29°C); *Baz depletion: Baz-mScarlet-I/Y; UAS>mRFP-Pon^LD^/UAS>RFP^RNAi^; Neur>Gal4, Jupiter-GFP / (MKRS)* (29°C)

### Method Details

#### Constructs

All plasmids used in this study were constructed by PCR-based and/or restriction site-based cloning methods and verifed by sequencing. All the Open Reading Frames (ORFs) cloned by PCR for this study were flanked by FseI and AscI sites for convenient shuttling between compatible plasmids. The version of GFP used throughout this study is “superfolder” GFP (sfGFP), referred to as GFP for convenience.

Murine versions of Par3 (180kDa isoform) Par6B, Par6G and aPKC_ι_ (referred to as aPKC for convenience) were cloned from murine cDNA. Par6A was cloned as a synthetic gene. Variants of these proteins were cloned using a quick-change protocol (Qiagen), to generate murine versions of established mutants/truncations, or combination thereof, and new variants based on established protein domain structures. A summary of all these variants is provided in Table 2 below for convenience. These constructs included Par3^ΔN^ (corresponding to AA 83-1319 from Par3 lacking the N-terminal oligomerization domain); Par3^ΔaPKC1^ (shorter 100kDa Par3 isoform lacking one of the two aPKC binding site which is known to be regulated by aPKC phosphorylation^31^; AA 1-740 + AA sequence ESGT). Note that while this truncation likely has further differences than just lacking aPKC binding, in this study, we investigated it solely in the context of its lack of aPKC binding, and hence refer to it as Par3^ΔaPKC1^ for clarity); Par3^ΔPDZ2^ (Par3 isoform lacking the second aPKC binding site which is known to be independent on aPKC phosphorylation as characterized in Holly et al.^31^); Par3^ΔPar6^ (Par3 Par6-binding mutant, G600,602A, Liu et al.^43^); Par3 phosopho mutant (S824,826A, noted SASA, murine equivalent of S827,829A, Lin et al.^30^); Par3 phosopho mimetic mutant (S824,826D, noted SDSD); aPKC Kinase active (A129E, noted aPKC^active^, murine equivalent of A120E mutant, Lim et al.^57^); aPKC Kinase dead (K274W, noted aPKC^dead^, characterized in Spitaler et al.^58^); aPKC Par6-binding mutant (E85A/E91A, noted aPKC^ΔPar6^, characterized in Hirnao et al.^26^); aPKC Par3-binding mutant (aPKC^Δ857-862^; noted aPKC^ΔPBM^; interaction characterized in Holly et al.^31^) Par6A aPKC-binding mutant (K19A, noted Par6A^ΔaPKC^ characterized in Hirano et al.^26^); Par6A N-terminus (Par6A^1-121^); Par6 C-terminus (Par6A^121-346^) and further sub-truncations (Par6A^121-257^, Par6A^247-346^ Par6A^121-346,169LGF:AAA^). We also generated a version of Par6A flanked by the “Jo” and “In” fragments in N-terminus and C-terminus, respectively. Jo/In which have the ability so covalently associate as described by Bonnet and colleagues (Bonnet et al.^32^), therefore generating a Par6 fragment whose conformation changes are likely perturbed (this construct is noted Jo-Par6A-In). Note that this reaction is not complete32 (i.e. some Jo-Par6A-In will be unaffected). Jupiter-iRFP670, a variant of the microtubule marker Jupiter-GFP^59^ where the GFP has been replaced with iRFP670^60^ was synthesized by IDT.

All ORFs were cloned into a pCDNA5/FRT/V5-His vector (Life technologies) for homologous recombination into the FRT site (Flip In system). This vector has been modified to be compatible with the MXS chaining system^61^ to allow polycistronic expression of different constructs from the same genomic locus. ORFs were expressed under the control of the EF1a promoter, or alternatively, for Doxycycline-inducible expression, the EF1a promoter was replaced by a Tet promoter, the MXS cassette CMV::rtTA3 bGHpA was ligated into the plasmid. ORFs were tagged at the N-terminus by GFP, mCherry, or the first 34 residues of the Mas70p protein (Mito Tag), shown to efficiently relocalize proteins to mitochondria in mammalian cells^62^ followed by a HA tag (referred to as Mito-HA).

Our transmembrane nanobody construct consists of an N-terminal signal peptide from the *Drosophila* Echinoid protein, followed by (His)_6_-PC tandem affinity tags, a nanobody against GFP^21^ (termed GBP for GFP Binding Peptide), a TEV cleavage site, the transmembrane domain from the *Drosophila* Echinoid protein, the VSV-G export sequence and a second copy of the GBP. This construct is referred to as GBP-TM-GBP. For direct fusion of proteins to the transmembrane domain, the internal GBP was replaced by the protein of interest (Figures S1C–S1F), or a stop codon (Figures S1A and S1B). We previously extensively characterized the clustering dynamics of a variant of this construct without the internal GBP.^20^

Most plasmids contained the EF1a Jupiter-iRFP670 cassette in order to image microtubules, but we verified that the (very dim) signals of this probe did not affect our immunofluorescence measurements in the far-red channel (Figures S3G and S3H).

For pull down assays in bacterial lysate, Par6A fragments were cloned into a modified pGEX vector to express a protein of interest downstream of the Gluthathione S transferase (GST) purification tag followed by TEV and 3C cleavage sequences. Similarly, Par6A fragments were cloned into a modified pET vector expressing the protein of interest downstream of (His)_6_ and Protein C (PC) purification tags.

For expression into ES cells, a piggyBac donor plasmid (kind gift from Prisca Liberali), was first rendered compatible with the MXS chaining system. Then, the cassette MXS EF1a::GBP-TM-GBP-SV40A (see above) and MXS_CMV::PuroR-bGHpA (Addgene # 62439) were ligated in. We refer to this plasmid as MXS piggyBac GBP-TM-GBP Puro.

For expression into liquid-cultured adapted S2 cells, we modified our custom inducible pMT vector bringing puromycin selection.^10^ The transmembrane segment we developed for mammalian cells (see above) was found not suited to reconstitute polarity in *Drosophila* cells because its steady state localization was not sufficiently restricted to the plasma membrane. We therefore developed a different system specifically for insect cells. The insect-specific transmembrane nanobody construct consists of an N-terminal signal peptide from the *Drosophila* Echinoid protein, followed by (His)_6_-PC tandem affinity tags, GBP, a TEV cleavage site, the transmembrane domain from the murine CD8a protein, and a second copy of the GBP. This construct is referred to as pMT puro GBP-CD8-GBP. Expression of the construct was induced for 2 days prior to the experiment by 0.6 mM CuSO_4_.

#### siRNA-mediated gene knockdown

Gene silencing using ON-TARGETplus siRNA (Dharmacon) was achieved by reverse transfection using Lipofectamine RNAi max according to the manufacturer’s instructions in 6-well dishes (day 1). The following day (day 2) the cells were transferred into a 10 cm-dish, and another 24h later, a reverse forward transfection was performed (day 3). On day 4 cells were washed with PBS and fresh medium has been added. Subsequently, on day 5, the cells have been used for respective experiments. The following siRNA oligonucleotides purchased from Dharmacon were used (5’-3’): ON-TARGETplus Mouse Camsap1 siRNA SMARTpool (L-045614-01-0005; #1: CAUCUUAGUGUUAGCGCUA; #2: GAGAGUAACCAUCGGACAU; #3: CCGCAAACCACCACGGCUU; #4: GUGCUGAAGCCGAA CGUUA), ON-TARGETplus Mouse Camsap2 siRNA SMARTpool (L-044067-00-0005; #1: GGAAAAGAGACGUGCGAUA; #2: GCAUUGAAGAAGCGUUACA; #3: CGGAGACCAUGGACGAAGA; #4: CGGGAUAGUUCAUCUAGUU), ON-TARGETplus Mouse Camsap3 siRNA SMARTpool (L-060823-01-0005; #1: GUACAACUCGGAUCGCAAA; #2: ACGCACAGGAGCACGUGAA; #3: GCGAUG UGGAUGUCGUCAU; #4: CUUUGGACCAGUACGAUUU), ON-TARGETplus Mouse Camsap3 individual siRNAs no.1 – no.4 (LQ-060823-01-0002; #1: GUACAACUCGGAUCGCAAA; #2: ACGCACAGGAGCACGUGAA; #3: GCGAUGUGGAUGUCGUCAU; #4: CUU UGGACCAGUACGAUUU), ON-TARGETplus Mouse Kif2A siRNA SMARTpool (L-041075-00-0005; #1: CAACAGAAUGGUAGCGUUU; #2: GAAAUGGUUUACAGGUUUA; #3: CUACACAACUUGAAGCUAU; #4: GGAAUUAGUCCUUCAGACA), ON-TARGETplus Mouse Kif2A individual siRNAs no.1 – no.4 (LQ-041075-00-0002; #1: CAACAGAAUGGUAGCGUUU; #2: GAAAUGGUUUACAGGUUUA; #3: CUACACAACUUGAAGCUAU; #4: GGAAUUAGUCCUUCAGACA), ON-TARGETplus Mouse Kif2B siRNA SMARTpool (L-064403-01-0005; #1: ACAAUACGAAUUCGGGAAA; #2: GGGCAGAACUCCUAUACGU; #3: ACGCAGGUGCUACGGGAUU; #4: GCGCAGUGACAAACGGAUU), ON-TARGETplus Mouse Kif2B individual siRNAs no.1 – no.4 (LQ-064403-01-0002; #1: ACAAUA CGAAUUCGGGAAA; #2: GGGCAGAACUCCUAUACGU; #3: ACGCAGGUGCUACGGGAUU; #4: GCGCAGUGACAAACGGAUU), ON-TARGETplus Mouse Kif2C siRNA SMARTpool (L-063980-00-0005; #1: UGACAGACCCUAUCGAAGA; #2: GAACUCGGAGCUAAGAAUA; #3: GAAUUUGCCCGGAUGAUCA; #4: GGAAAUCGUGUAUCGUGAA), ON-TARGETplus Mouse Kif2C individual siRNAs no.1 – no.4 (LQ-063980-00-0002; #1: UGACAGACCCUAUCGAAGA; #2: GAACUCGGAGCUAAGAAUA; #3: GAAUUUGCCCGGAUGAUCA; #4: GGAAAUCGUGUAUCGUGAA).

#### Protein expression and purification

A(d), A(d)-mCherry, B(c)-GFP, B(c)-Spy Catcher, Spytag-DLL4 and GFP proteins were expressed and purified as described.^20^ SpyTag-DLL4 was purified as described.^48^ B(c)-SpyCatcher: SpyTag-DLL4, referred to as B(c)-SC:ST-DLL4, was generated by mixing the two proteins in a 2:1 ratio overnight at 4°C.

#### In vitro binding between Par6 domains

Plasmids expressing (or not) fragments of Par6 were transformed into BL21 bacteria and grown in 2XTY at 37°C, before induction with 1 mM IPTG at an OD280 of 0.8, and expression overnight at 18°C. Bacteria were then rinsed with PBS, before lysis on ice in 20 mM HEPES, 100 mM Potassium Acetate, 5% glycerol, 1% Triton X-100, 1X cOmplete protease inhibitors (Roche), 10 μg/ml DNaseI, 5 mM MgCl_2_, 1 mg/ml lysozyme and 1 mM DTT for 10 minutes. Cells were then centrifuged at 20,000 x *g* for 20 minutes at 4°C. Lysates from the different expressions were then mixed, and incubated with GST-4B resin for 1 hour at 4C. The resin was subsequently washed thrice with 20 mM HEPES, 100 mM potassium acetate, 5% glycerol, before boiling in LDS loading buffer and processing for western blot.

#### SDS-PAGE and Western blot

SDS-PAGE was performed using NuPAGE 4-12% Bis-Tris gels (Life Technologies) according to the manufacturer’s instructions. Gels were transferred on nitrocellulose membranes using an iBLOT (Life Technologies) according to the manufacturer’s instructions. Following Ponceau staining (Sigma), membranes were washed in TBS and blocked in TBS enriched with 5% milk powder or 3% PBS/BSA for 20 min at RT. Primary antibodies were incubated overnight at 4°C using a solution made of 1 μg/ml antibody in TBS supplemented with 1 mM CaCl_2_, 0.2% BSA and 0.02% Thymerosal. The membranes were then incubated using fluorescent antibodies (1/500 dilution in TBS supplemented with 1mM CaCl_2_ and 5% milk powder or 1% BSA), then revealed using a fluorescent scanner (Typhoon).

#### Antibodies

All antibodies were used at 1 μg/mL for both Western Blot and immunofluorescence. Atto-647N-labelled anti-α K40 acetylated tubulin (C3B9, HPA Cultures) antibody was prepared as previously described.^5^ Mouse monoclonal antibody HPC4 (against the PC tag) was from Roche. Rabbit polyclonal antibody against Par3 was from Merck (#07-330). Mouse monoclonal antibody against aPKC was from Santa Cruz (sc-17837). Rabbit polyclonal anti-GST antibody was from Abcam (#ab19256). Rat monoclonal anti-HA antibody (clone 3F10) was from Roche. Rabbit polyclonal anti-Camsap2 was purchased from Proteinbiotech (17880-1-AP), and rabbit polyclonal anti-Kif2A was purchased from Novus Biologicals (NB500-180). Alexa-555 and Alexa-A647-conjugated anti-rabbit secondary antibodies used were purchased from Thermo (A32732, A21246). Alexa-647-conjugated anti-mouse secondary antibody was also purchased from Thermo (A-11018). Donkey Cy3-conjugated anti-rat antibodies were purchased from Jackson ImmunoResearch.

#### Array assembly on cells and live cell imaging

Clustering the transmembrane construct was achieved through assembly of our two-component hexagonal arrays, essentially as described previously.^20^ These two components are named A(d) and B(c)-GFP, where B(c)-GFP binds to the transmembrane segment (via the anti GFP nanobody), and A(d) clusters B into a hexagonal array of fixed dimensions. Briefly, cells stably expressing the transmembrane construct were trypsinized and spread on Fibronectin-coated (50 μg/ml in PBS, 30 minutes at RT) imaging dishes (World Precision Instruments, FD35 or FD3510) for 1 hour at 37°C/5% CO_2_. Cells were then incubated for 1 minute with 0.5 μM B(c)-GFP in growth medium then washed once with medium, before the addition of 0.5 μM A(d) in medium for 10 minutes (or less, if clustering was to be imaged at an earlier time point). Cells were then washed with medium to remove free array components.

When working with cells co-expressing GBP-TM-GBP and a GFP fusions, some extracellular GBP could be quenched with GFP-fusion protein in the medium due, for example, to cell death. To avoid this potential issue, cells are always treated with a quick acid wash prior to GBP-TM-GBP clustering. Specifically, cells were briefly washed thrice with 0.1 M glycine, 150 mM NaCl, pH 3.0 to remove any bound extracellular GFP, before washing with medium. Importantly, we verified that this acid washing step did not affect the clustering efficiency nor our colocalization results (Figures S3E and S3F).

To image cells through division (Figure 4), cells were synchronized in mitosis with 30 nM nocodazole for 6-12 hours at 37°C after clustering. Cells were then gently washed twice with imaging medium, before imaging at 37°C in L15 medium (Gibco), 10% Donor Bovine Serum (Gibco) enriched with 20 mM Hepes (Gibco).

For sub-cellular light sheet imaging, 3T3 cells expressing GBP-TM-mScarlet were spread onto 5mm glass coverslips coated with fibronectin as above. Cells were then incubated with 0.5μM B(c)-GFP as above, followed by A(d) and imaging in Hepes-enriched L15 medium. For oblique plane light sheet imaging, cells were spread onto Fibronectin-coated imaging dishes (World Precision Instruments, FD35) as above, and array formation was induced with 0.5 μM B(c)-GFP for 1 min at 37°C in growth medium on a hot plate, then washed once with medium, before the addition of 0.5 μM A(d) in growth medium for 5 min at 37°C in growth medium on a hot plate. Cells were then washed and imaged in growth medium before addition of trypsin (Figure 1D). Alternatively, cells were stalled in mitosis with 30 nM nocodazole for 12 hours at 37°C/5%CO_2_, then imaged in Hepes-enriched L15 medium (Figures S2I and S2J).

For the cap formation from Notch receptors (Figure S2G), U2OS expressing Notch receptors tagged externally with GFP were incubated with 250 nM B(c)-SC:ST-DLL4 for 5 minutes, and then with A(d)-GFP for a further 5 minutes, both in culture medium. Arrays were further grown by seven cycles of sequential incubations with B(c)-SC:ST-DLL4 then A(d), each of 1 min. Cells were then incubated with 1 μM nocodazole overnight, and imaged in medium containing nocodazole.

For cap formation in ES cells (Figure S2D), 40,000 mouse ES cells stably expressing GBP-TM-GBP were plated on a gelatin-coated well of an ibiTreat m-Slide 8 Well plate (80826, Ibidi) in N2B27 2i/LIF medium. N2B27 basal medium contains a 1:1 mix of DMEM F12 (21041025, ThermoFisher Scientific) and Neurobasal A (12348017, ThermoFisher Scientific) supplemented with 1% v/v B27 (10889038, ThermoFisher Scientific), 0.5% v/v N2 (homemade), 100 μM β -mercaptoethanol (31350-010, ThermoFisher Scientific), penicillin-streptomycin (15140122, ThermoFisher Scientific) and GlutaMAX (35050061, ThermoFisher Scientific). 1 hour after plating, cells were treated with 0.2 μM B(c)-GFP in ice-cold PBS for 1 min on a cold metal plate on ice, then washed once with cold PBS, before the addition of 0.2 μM A(d) in cold PBS for 4 min on ice, then warm N2B27 2i/LIF medium was added and cells were imaged by SDC. Note that if A(d) is omitted, there is no cap formation (Figure S2D).

For cap formation in suspension-adapted *Drosophila* S2 cells (Figure S2E), cells having stably incorporated the plasmid pMT Puro GBP-CD8-GBP were induced for 2days with 0.6 mM CuSO_4_. Array assembly in this liquid culture was then triggered by adding B(c)-GFP 5 mL of cells at 10^6^ cells/mLin Insect Express medium (0.5 μM final) for 30 min, then cells were pelleted, washed in 10 mL Insect Express medium, then resuspended in 1 mL Insect Express medium containing 0.5 μM A(d). After 5 min, cells were washed twice in 10 mL Insect Express medium and plated on imaging dishes (World Precision Instruments, FD35) precoated with Concanavalin A (0.05 mg/ml final in PBS, Sigma L7647-25MG) for cells not to move during volumetric imaging by SDC. While caps were readily observed in suspension cells, we noticed that that cells quickly start to spread in this experiment (because conA induces spreading), which led to loss of the spherical shape of the cells and thus of their caps (Figures 1 and S3).

Note that the conditions for cap formation are slightly different between the different cell types used in this study (U2OS, 3T3, S2 and ES). With our method, the efficiency of cap formation depends on the efficiency of array formation, and in particular that arrays get large enough, fast enough to avoid endocytosis, as we previously established.^20^ Array size can be tuned with our method, and depends mostly on the concentration of A, the density of the transmembrane segment at the plasma membrane, and the constitutive endocytic rate of the transmembrane segment.^20^ Thus, it is expected that different cell lines expressing different levels of the transmembrane segment and having different endocytosis efficiency will give different degrees of array assembly and thus cap formation. But this can be compensated by adapting the concentrations of A and B(c)GFP, or by limiting constitutive endocytosis can also be modulated by cooling down the cells (array assembly is not affected by temperature). Therefore, anybody wanting to establish the system in a cell line than those studied above will need to slightly adapt our protocols to reach the high reproducibility of cap formation we obtained in 3T3 cells (Figure S3G). We suggest making stable cell lines rather than transient transfections to ensure that the transmembrane segment is expressed at the same level in all cells.

Note that throughout this paper, sample size was not determined *a priori*, but was always sufficiently large to permit meaningful statistical analysis. All sample numbers are detailed in fgure legends or fgure panels.

#### Immunofluorescence

For fixed cell imaging of the central spindle, mitotic cells were either isolated using the mitotic shake-off method, or cells were prepared in the same way as for division assays, except that, after the nocodazole block, cells were washed with DMEM medium and incubated at 37°C/5% CO_2_ for 30 min, to allow cells to reach anaphase.

For the mitotic shake-off, cells were gently washed with PBS, 250-500 μl imaging medium was then added to the 10 cm dish, and each side of the dish was tapped 10-15 times. The medium was then collected and transferred onto Fibronectin-coated ibidi 4-well chamber slides. The slides were subsequently incubated for 20 min at 37°C to allow attachment of the mitotic cells.

Cells were then fixed with 4% paraformaldehyde (PFA) in PBS for 20 minutes at room temperature. Cells were then washed in PBS and subsequently permeabilized with 0.1% Triton X-100 for 5 minutes, before washing and incubation with 1% bovine serum albumin (BSA, Fisher, BP1605) for 10 minutes. Cells were then stained with Atto-647N-labelled anti-α K40 acetylated tubulin antibodies, rabbit anti-Camsap2, or rabbit anti-Kif2A before imaging in PBS + 0.1% BSA.

For fixed cell imaging of Par complex assembly after clustering, cells at the indicated time post-clustering, were fixed as above. Cells were stained with anti aPKC and/or anti Par3 antibodies for 1 hour at RT in PBS + 0.1% BSA. After washing thrice, cells were stained with secondary antibodies for 1 hour in PBS + 0.1% BSA. Cells were then imaged in PBS. Note that while most stable cell lines plasmids contained an EF1a Jupiter-iRFP670 cassette in order to image microtubules, we verified that the dim signals of this probe did not affect our immunofluorescence measurements in the far-red channel (Figures 3G and 3H). In fact, Jupiter-IRFP670 is a weak microtubule marker and its signal can only really be sporadically detected at the central spindle in anaphase (Figure 4).

For fixed cell imaging of mitochondrial relocalization, NiH/3T3 Flp-In cells were transiently transfected with the indicated plasmids for 24 hours. Cells were then trypsinized and spread on Fibronectin-coated dishes for 1 hour. Cells were then fixed and stained as above, with an anti-HA antibody, and imaged in PBS.

#### Flow cytometry

To measure the density of active GBP-TM-fusions at the surface of cells as a function of the expression level of each construct (Figures S1D–S1F), stable 3T3 cells expressing GBP-TM-fusions under Doxycycline-inducible promoter were treated with varying doses of Doxycycline for 24 h, then cells were incubated with 1 μM purified GFP in serum/HEPES-supplemented L-15 medium for 1 min at RT, then washed in PBS-1 mM EDTA and trypsinized and resuspended in serum/HEPES-supplemented L-15 medium. GFP-fluorescence per cell was then measured by Flow cytometry in an iCyt Eclipse instrument (Sony) using a 488 nm laser. Data analysis was performed using the supplier’s software package.

#### Fly notum immunofluorescence

Dissected fly nota were fixed according to a method designed to preserve the microtubule cytoskeleton.^48^ Briefly, nota were first incubated in Hank’s balanced salt solution (Gibco) enriched with 1mM DSP (Pierce) for 10 min at RT followed by a 10 min incubation in MTSB (microtubule stabilization buffer: 0.1M PIPES, 1mM EGTA, 4 % PEG 8000, pH 6.9) enriched with 1mM DSP, then finally in MTSB enriched with 4% PFA (Electron Microscopy Science). Nota were then permeabilized in MTSB enriched with 4% PFA and 0.2% Triton X100 then processed for immunofluorescence using Atto-647N-labelled anti-α K40 acetylated tubulin (C3B9, HPA Cultures) antibody as described^5^ and mounted in Prolong Gold antifade reagent (Molecular Probes). SOP live-cell imaging was performed in Clone 8 medium after embedding into a fibrinogen clot in order to diminish tissue movements during fast 3D image acquisition as described.^63^ Imaging was performed on the Spinning Disk confocal microscope described below.

#### Fluorescence Lifetime Imaging Microscopy (FLIM)

3T3 cells stably expressing GBP-TM-VSVG-mScarlet were spread on fibronectin-coated glass-bottom dishes, and the transmembrane construct was then clustered with B(c)-GFP and A(d) as described above, before mitotic stalling was induced with 30 nM nocodazole for 12 h. The Flipper-TR probe (Spirochrome, see Colom et al.^22^) was then added (2 μM final in L15-20 mM HEPES medium) and Flipper-TR fluorescence lifetime imaging was performed on a setup comprising a Zeiss LSM710 stand, a 63X NA 1.4 oil objective, a Zeiss 710 confocal scanner head and Time-Correlated Single Photon Counting (TCSPC) hardware from Picoquant. A 470 nm pulsed laser (Picoquant), operating at 40 MHz was used to excite the probe, and detection was performed on a gated PMA hybrid 40 detector (Picoquant) behind a 600/50 bandpass filter (Semrock). SymPhotime 2.0 software (Picoquant) was used for data analysis. Flipper-TR fluorescence lifetime was fitted to a dual exponential model. The intensity-weighted average lifetime in a Regions of Interest (ROI) encompassing either the array-containing membrane (assessed by GFP signal), or regions of the membrane devoid of arrays was then measured, followed by averaging over several cells.

#### Microscopy

All confocal and Total Internal Reflection Fluorescence (TIRF) imaging was performed using a custom TIRF/spinning disk confocal microscope composed of a Nikon Ti stand equipped with perfect focus, a fast piezo z-stage (ASI) and a Plan Apochromat lambda 100X NA 1.45 objective (immunofluorescence experiments and TIRF) or a Plan Apochromat lambda 60X NA 1.4. The confocal imaging arm is composed of a Yokogawa CSU-X1 spinning disk head and a Photometrics 95B back-illuminated sCMOS camera operated in global shutter mode and synchronized with the spinning disk rotation. The TIRF imaging arm is composed of an azimuthal TIRF illuminator (iLas2, Roper France) modified to have an extended field of view to match the size of the camera (Cairn). Images were recorded with a Photometrics Prime 95B back-illuminated sCMOS camera run in pseudo-global shutter mode and synchronized with the rotation of the azimuthal illumination. Excitation was performed using 488 nm (150 mW OBIS LX), 561 nm (100 mW OBIS LS) and 637 nm (140 mW OBIS LX) lasers fibered within a Cairn laser launch. To minimize bleedthrough, single band emission filters were used (Chroma 525/50 for Alexa 488/GFP; Chroma 595/50 for mScarlet/Alexa 555/mRFP/mCherry and Chroma 655LP for Alexa647/ATTO647N) and acquisition of each channel was performed sequentially using a fast filter wheel in each arm (Cairn Optospin). To enable fast acquisition, the entire setup is synchronized at the hardware level by a Zynq-7020 Field Programmable Gate Array (FPGA) stand-alone card (National Instrument sbRIO 9637) running custom code. In particular, fast z-stacks are obtained by synchronizing the motion of the piezo z-stage during the readout time of the cameras. Sample temperature was maintained at 37°C for microtubule dynamics using a heating enclosure (MicroscopeHeaters.com, Brighton, UK). Acquisition was controlled by Metamorph 7.10.1.161 software.

Sub-cellular light sheet imaging (Figure S1B; Video S1) was performed on a home-built field synthesis^63^ light sheet microscope featuring a 0.7 NA excitation lens (54-10-7, Special Optics) and a 1.1 NA detection lens (CFI75 Apo 25XC W MRD77220, Nikon). Illumination light was provided from 405 nm, 488 nm, 561 nm and 638 nm lasers (LBX-405-100-CSB-PPA, LBX-488-100-CSB-PPA and LBX-638-100-CSB-PPA, Oxxius or OBIS 561 nm LS 100 mW, Coherent, respectively). Fluorescence was collected by the detection objective and directed through an f = 500 mm doublet onto the camera (Pco Edge 4.2, PCO, giving a sample pixel pitch of 104 nm), through appropriate bandpass filters (Chroma). The sample was coarsely positioned in XY using a stack of piezo-driven translation stages (8525, Newport) and was moved through a fixed sheet during imaging using a fast-stepping piezo drive (P-621.1CD, PI). The sample was mounted on a 5 mm diameter circular cover slip (AGL46R5-1, Agar Scientific) held in a stainless steel ’spoon’. The sample was submerged in L-15 medium supplemented with HEPES held in a gold-plated copper bath, which was resistively heated at 37°C. We noted that the imaging performance of the detection objective was highly sensitive to the exact setting of the correction collar, which was manually iteratively optimized before every imaging session.

Oblique plane light sheet imaging^40,41^ (Figure 1E; Videos S4 and S5) was performed on a home-built, high-resolution system, featuring a 1.35 NA silicone immersion primary lens (CFI SR HP Plan Apo Lambda S 100XC Sil MRD73950, Nikon), 0.95 NA air immersion secondary lens (CFI Plan Apochromat Lambda D 40X, MRD70470) and a 1.0 NA solid immersion tertiary lens (AMS-AGY v1 54-10-5, Applied Scientific Instrumentation). Illumination light was provided from 405 nm, 488 nm, 561 nm and 638 nm lasers (LBX-405-100-CSB-PPA, LBX-488-100-CSB-PPA and LBX-638-100-CSB-PPA, Oxxius or OBIS 561 nm LS 100 mW, Coherent, respectively). Fluorescence was collected by the primary objective and directed onto a camera (BSI Express, Photometrics), through appropriate band-pass filters (Chroma). The sample was positioned in XY using a motorized stage (MS-2000, Applied Scientific Instrumentation) and in Z using a piezoelectric stage (Z-Insert, Piezoconcept). During imaging, the light sheet and image plane were moved through the stationary sample using a galvanometric mirror (GVS001, Thorlabs). The samples were mounted in an imaging dish (Fluorodish, World Precision Instrument), held in a stage-top incubator (H301-MINI, Okolab) which provided warmth, CO_2_ and humidity.

#### Image processing

Unless stated otherwise, images were processed using Fiji^53^ and Matlab 2020b (Mathworks) using custom codes. Figures were assembled in Adobe Illustrator 27.3.1. Movies were edited in Adobe Premiere 2021.

Spatial drift during acquisition was corrected using a custom GPU-accelerated registration code based on cross correlation between successive frames. Drift was measured on one channel and applied to all the channels in multichannel acquisitions. Code is available on our github page (https://github.com/deriverylab / GPU_registration).

For representation purposes, all confocal images of Par clusters in colocalization figures (Figures 1, 2, 3, S1, and S4–S8) were processed with a Wavelet “à trous” filter (custom GPU-accelerated MATLAB port of a code originally developed by Fabrice Cordeliere for the “Improve Kymo” ImageJ plugin^64^ and the raw image was averaged with the filtered one to generate the figure panel. Code is available on our Github page (https://github.com/deriverylab / GPU_wavelet_a_trous). Note that the colocalization analysis (see below) was performed on the raw 3D stacks.

##### Subcellular light-sheet data processing

Image volumes obtained by sub-cellular light sheet microscopy and oblique plane light sheet microscopy were computationally deskewed using our home-made software suite lsfm_tools implementing a linear interpolation (https://github.com/jdmanton/lsfm_tools). CudaDecon (https://github.com/scopetools/cudaDecon) was used to deconvolve data using the Richardson-Lucy algorithm with a theoretical PSF (Born and Wolf model) generated with the ImageJ plugin PSF generator.^65^ Deskewed volumes were visualized via maximum intensity projection.

##### 3D reconstruction

For 3D reconstruction (Figures 1 and S2; Videos S1 and S3), confocal z-stack of cells (Δz=200 nm) were acquired, and deconvolved using the Richardson-Lucy algorithm and a theoretical PSF with the program suite lsfm_tools described above. 3D rendering was then performed with Napari software.^66^ For Figures 1C, 1D, and S2B, cell surface was then automatically segmented in 3D using the Fiji plugin LimeSeg developed by Machado and colleagues,^54^ then 3D rendering was performed using Amira software.

##### Colocalization

To automatically measure the colocalization between clusters and endogenous proteins in fixed samples, we used an object-based method where two objects are considered colocalized if the distance between their fluorescence centroid is below a certain threshold *r_ref_*.^67^ Particles were automatically detected in multichannel confocal z-stacks (Δz=200 nm) using 3D Gaussian PSF fitting developed by Aguet and colleagues^68^ (https://github.com/francois-a/llsmtools). This also provided the integrated intensity of each spot considering the local background. We excluded from the analysis all arrays localized at the ventral side of the cell to avoid potential artefacts due to interactions of the arrays with the coverslip, or to differential membrane tension between the ventral and dorsal sides of the cell. To achieve this, the coverslip was automatically detected, and all arrays found within 3 planes of the coverslip (600 nm) were automatically excluded. This also provided the integrated intensity of each spot considering the local background.

Once this automated detection has been performed in all channels, the distance *d_AB_* between all particles in 3D in the two channels (*A* and *B*) are computed and compared to a reference distance *r_ref_*. If *d_AB_* < *r_ref_*, the particles detected in the two channels are deemed to colocalize.

To set *r_ref_* we followed the method implemented by Cordelières and Bolte in the ImageJ plugin JACop 2.0,^67^ and calculated *r_ref_* for the 3D situation using the following equations:

$$\Phi =\arccos\frac{\left(x_{B}-x_{A}\right)}{\sqrt{{\left(x_{B}-x_{A}\right)}^{2}+{\left(y_{B}-y_{A}\right)}^{2}}}\text{and}\;\Theta =\arccos\frac{\left(z_{B}-z_{A}\right)}{\sqrt{{\left(x_{B}-x_{A}\right)}^{2}+{\left(y_{B}-y_{A}\right)}^{2}+{\left(z_{B}-z_{A}\right)}^{2}}}$$

$$r_{\text{ref}\;}=\sqrt{{\left(resol_{xy}\sin\Theta \cos\Phi \right)}^{2}+{\left(resol_{xy}\sin\Theta \sin\Phi \right)}^{2}+{\left(resol_{z}\cos\Theta \right)}^{2}}$$

Here, *x_A_*,*y_A_*,*z_A_* and *x_B_*,*y_B_*,*z_B_* are the 3D coordinates of particles in channel *A* and *B*, respectively, and *resol_xy_* and *resol_z_* correspond to the lateral and axial resolutions of the microscope, respectively. For our analysis, we measured *resol_z_* = 0.498 *μm* and *resol_xy_* = 0.293 *μm* using 0.2 μm TetraSpeck beads from Invitrogen.

Once all the particles have been detected and their colocalization state addressed (i.e.*d_AB_* < *r_ref_*), we measured the percentage of colocalization as the fraction of the total signal contained in particles that do colocalize, namely:

$$\%\;of\;colocalization\;=\frac{\sum \;colocalizing\;particles\;}{\sum \;total\;particles\;}*100$$

This measurement was then averaged between cells and compared between stable cell lines and time points. For each graph, a sample where only GFP is clustered (rather than GFP-Par6A for instance) is plotted to serve as an internal control to show the accuracy of the method (any colocalization with GFP alone is assumed to reflect non-specific colocalization due to the random localization and density of spots in both channels).

To ensure that variations of laser intensity or alignment within our instrument, as well, as variations of the efficiency of the immunostaining (due to deterioration of the primary/secondary antibody stocks for instance) would not lead to variation of the absolute levels of colocalization between datasets because different samples would not be as efficiently segmented, we always verified that we had the same average number of particles detected per cell in the non-array channel in the controls, and slightly tweaked the segmentation parameters if needed.

##### Central spindle asymmetry measurements

Central spindle asymmetry was measured by computing the difference of microtubule density (assessed by acetyl-tubulin immunostaining) between the two sides of the central spindle, rather than the absolute amounts of tubulin. This is because we previously established that what matters for polarized trafficking of signaling endosomes is the ratio of tubulin densities between the two sides of the spindle, rather than the absolute amount of tubulin.^5^ Briefly, we first projected z-stacks containing the entire central spindle (6 μm depth, Δz = 0.2 μm) using max-intensity projection. We focused on cells fixed shortly before abscission as at this stage, the Ac-tubulin signal at the central spindle has the characteristic hourglass shape of late mitotic spindles. We can thus use this characteristic shape as a registration cue to register spindles from different images and average them, as we previously established.^5^

The intensity of the tubulin staining signal was then measured along central spindle length (x-axis) upon signal integration over the spindle width (y-axis, parallel to the division plane) within a region of interest (ROI) centered on the central spindle. This measurement thus conceptually resembles a linescan along the length of the spindle (x-axis), but where not a line, but a rectangular ROI is considered (ROI dimensions: 10 μm on the x-axis and spindle width on the y-axis). For this reason, we previously named it “pseudo-line-scan” method. The signal intensity over the x-axis determined this way displays two peaks: one in pIIa, one in pIIb, see Figure 5C for example. This reflects the facts that most markers are excluded (at least in part) from the core of the central spindle. These “pseudolinescans” were then averaged between different cells to yield the panels presented in Figures 5C and 5F.

We then measured the value of each peak and subtracted the local background (average background was determined from 5 pixels adjacent to the spindle). Central spindle asymmetry was computed as the normalized enrichment of the density of the marker in the anterior side according to:

$$\Delta =\frac{\text{Peak}\;\text{intensity}\;\text{anterior}-\text{Peak}\;\text{intensity}\;\text{posterior}}{\text{Peak}\;\text{intensity}\;\text{anterior}+\text{Peak}\;\text{intensity}\;\text{posterior}}$$

Note that Δ is symmetrical when anterior and posterior cells are inverted and that − 1 ≤ Δ ≤ 1. Antero-posterior polarity was provided by the Pon^LD^ signal for fly SOPs, or by the localization of the artificial cap for 3T3 cells (by convention, the cap-containing cell is the posterior cell as Par3/Par6/aPKC are posterior Par proteins). This value was then averaged per cell to yield Figure 5G.

##### Automated tracking

To analyze array motion in Oblique Plane light sheet microscopy experiments (Figures S2I and S2J), Array position was first determined by 3D PSF fitting deskewed stacks using the as above (see 3D colocalization). Array trajectories were then tracked in 3D using the MATLAB adaptation by Daniel Blair and Eric Dufresne of the IDL particle tracking code originally developed by David Grier, John Crocker, and Eric Weeks (http://site.physics.georgetown.edu/matlab/index.html) in MATLAB 2020b. For each track, the weighted Mean Square Displacement (MSD) of segments of increasing duration (delay time *t*) was then computed (*MSD* (*t*) = < (Δ*x*)^2^ > + < (Δ*y*)^2^ >) using the MATLAB class MSD Analyzer.^69^ Note that the weighted mean gives more weight to MSD curves that have greater certainty. Weighted MSD curve was then fitted to a subdiffusion model captured by *MSD*(*t*) = 6*Dt^α^* with *D* the effective diffusion rate and *α* the anomalous coefficient. This provided an estimate of *α* = 0.65 ± 0.04 and *D* = 0.007 ± 0.001 for a dataset of 137 tracks (error corresponds to 95% confidence interval, *R*^2^ = 0.99). A value of *α* significantly below 1 indicates a confined diffusion.

#### Rationale of Par complex assembly pathway

Exactly how the three core Par complex components (Par3, Par6 and aPKC) interact with one another is a long-standing conundrum in the field. All three components are known to harbor conserved binding sites for the other components, yet the tripartite complex is not always assembled. It has often been challenging to reconcile *in vitro* data with data in cells, and to explain the different assembly states of the Par complex in different cell types and subcellular locations. In this work, through our synthetic manipulation of the system *in vivo*, we harmonize and extend previous work to provide a fuller description of how the Par complex assembles. Here, we go into more detail about how the assembly scheme depicted in Figure 3G was untangled and resolved.

It must be emphasized that all of the data on Par complex assembly detailed in this paper is highly consistent with previous work. We have confirmed the following findings:

- Par6 and aPKC can interact, with aPKC^E85/R91^ and Par6^K19^ vital for this interaction.^26^
- Par6 and Par3 can interact, with Par3^G600,602^ and Par6^121–257^ vital for this interaction.^43^
- Full-length Par3 and aPKC can interact, with phosphorylation of Par3 by aPKC reducing this interaction.^30^
- Clustering/oligomerization of Par3 is required for Par complex assembly^30^ (Figure S6).
- The Par complex can assemble in clusters at cell-cell junctions.^44^

Through synthetic manipulation of individual Par complex components and mutants in 3T3 cells, we extend and reconcile these sometimes seemingly contradictory findings, which collectively suggest the molecular model depicted in Figure 3G:

- The tripartite Par complex is mostly unassembled in single cells, with Par complex components largely cytoplasmic.
- Clustering of any core Par complex component can induce assembly of the tripartite complex.
- While Par3 phosphorylation quite mildly inhibits Par complex assembly, the Par complex can still assemble in the absence of either Par3-aPKC interaction sites, instead assembling through Par6.
- The aPKC kinase activity is dispensable for Par complex assembly, although its inhibition may stabilize the aPKC-Par3 interaction post-Par complex assembly.
- Par6 exists in an autoinhibited state, with the N- and C-termini interacting to prevent binding to aPKC and Par3 respectively.
- Clustering of any of the three components can relieve the autoinhibition of Par6 to allow binding to aPKC and Par3, but Par6 is most readily ‘opened’ by aPKC.
- The role of the oligomerization domain in Par3 in Par complex assembly is solely to cluster Par3 and permit ‘opening’ of Par6.

These statements will be justified in the following sections.

##### Par is cytoplasmic and incompletely assembled

While Par3 and aPKC (and presumably Par6) colocalize in puncta at cell junctions (Figures S3A–S3C), in naive 3T3 cells, the components are largely cytoplasmic (Figure S3C). Colocalization cannot be reliably assessed with this diffuse protein distribution, so we sought to assess colocalization 2 minutes post induction of clustering, where assembly of lattices has long reached steady state (Figure 1B; Ben-Sasson et al.^20^), and the clustered Par complex components hence appear as diffraction limited spots. At this time point, after clustering Par6, there was very little triple colocalization with both Par3 and aPKC (Figures 2B and 2C), suggesting the three components are unassembled. Furthermore, the intensity of aPKC in Par6 clusters was found to increase over time, confirming a gradual recruitment of aPKC onto these clusters, which goes against the idea of a preassembled state of the Par6/aPKC heterodimer before clustering (Figure S4C). While endogenous Par6 could not be directly visualized, Par3 was largely absent from induced aPKC clusters at 2 minutes (Figure S5C), and *vice versa* (Figure S6C). Therefore, we conclude that the tripartite Par complex is natively predominantly unassembled in 3T3 cells.

##### Clustering Par induces complex assembly

Synthetic clustering of either Par3, Par6 or aPKC all led to the progressive assembly of the full Par complex. Clustering Par6 led to the recruitment of aPKC followed by Par3 (Figures 2C and S4). Clustering aPKC led to the recruitment of Par3 (Figure S5) and Par6 (Figures S4G–S4I), and similarly, clustering of Par3 led to the recruitment of aPKC and presumably Par6 (Figure S6).

##### aPKC-Par3 is dispensable for assembly

As aforementioned, aPKC clustering induces recruitment of Par6 and Par3. Par3 recruitment onto aPKC can be either direct, or indirect via Par6 binding, since Par6 also directly bind to Par3. It is established that aPKC can directly bind to Par3 via two, independent biding sites: i) a phospho-regulated interaction between the kinase domain of aPKC and the aPKC phosphorylation motif on Par3^19^ (also known as CR3 domain), and ii) a phospho-independent interaction between the PBM motif of aPKC and the PDZ2 domain of Par3.^31^ But it is currently not clear if both direct and indirect interactions are needed for Par complex assembly.

Our data suggest that the direct interaction between aPKC and Par3 is dispensable for Par complex assembly in the presence of Par6. This is evidenced by five lines of observations:

- Abolishing the aPKC-Par6 interaction (aPKC^ΔPar6^) completely abolishes Par3 recruitment, irrespective of the kinase activity of aPKC (compared to aPKC^active ΔPar6^ and aPKC^dead ΔPar6^) (Figure S5).
- An aPKC-ΔPBM mutant recruited endogenous Par3 with the same kinetics as *wild type* aPKC in the presence of Par6 (Figures S5C, S5E, and S5K).
- Clustering a double phosphomimetic Par3 on the phoso-regulated aPKC-binding site (S824 and S826, mouse equivalents of sites previously shown to inhibit binding to aPKC when phosphorylated^30^ led to Par complex assembly not significantly different to clustering the WT Par3 isoform (Figure S6G).
- Clustering of a mutant of Par3 that lacks both the phospho-regulated and phospho-independent aPKC binding sites (noted Par3^ΔaPKC1 ΔPDZ2 ΔN^) induced assembly of the Par complex (Figures S7D–S7G). In this case, as we ensured binding to alternative, native Par3 isoforms was abolished through removal of the N-terminal oligomerization domain, we conclude that Par complex assembly induced by Par3 clustering must also occur through Par6 (Figures S6D–S6G). This was confirmed when we mutated the Par6 binding site on this Par3 mutant (Par3^ΔaPKC1 ΔPDZ2 ΔN ΔPar6^) and found that it was indeed unable to recruit aPKC (Figures S6D–S6G). Note that Par3^ΔaPKC1 ΔPDZ2 ΔN^ reproducibly induced much stronger aPKC recruitment than Par3 (Figures S6D–S6G). The reason for this is unknown and will require more investigations. It is possible that the ΔPDZ2 mutant affects the release of autoinhibition of Par3, which has been proposed (e.g. Chen and colleagues,^70^ who showed that the C-ter of Par3, containing the PDZ domains affects autoinhibition).
- While clustering Par6 leads to the recruitment of aPKC and Par3 (Figure 2C), the interaction between aPKC and Par3 does not seem required for the recruitment of Par3 onto aPKC-Par6. This is evidenced by Figures 3A–3E, where clustering of the N-terminus of Par6 (containing the aPKC binding site) can recruit only aPKC, and clustering of the C-terminus of Par6 (which contains the Par3 binding site) can recruit only Par3. The fact that the N-terminus of Par6 bound to aPKC cannot recruit Par3, and the C-terminus of Par6 bound to Par3 cannot recruit aPKC confirms again that the aPKC-Par3 interaction is insufficient to assemble the tripartite complex.

Altogether, these results strongly suggest that a direct binding between aPKC and Par3 is not required for assembly of the tripartite Par complex in our system. In other words, Par6 is the intermediary between aPKC and Par3, rather than the aPKC-Par3 interaction driving Par complex assembly.

Nevertheless, our data are fully consistent with the fact that the phosphorylation of Par3 can reduce its direct binding to aPKC. Indeed, while removing the phosphoregulated aPKC-binding site on Par3 did not affect aPKC recruitment (Par3^ΔaPKC1^ vs Par3 or Par3^ΔaPKC1 ΔN^ and Par3^ΔN^), we could detect a small effect when we clustered S824/S826 Par3 phosphomutants, with the phosphomimetic (SD) Par3 leading to slightly less aPKC recruitment and conversely the phosphomutant (SA) Par3 slightly more, as expected from previous work (Figure S6G). This suggests that the phospho-state of Par3 can mildly affect Par complex assembly. But the fact that this effect is small is fully consistent with all the evidence discussed above, demonstrating that the actual route used in our system for Par complex assembly does not utilize the direct recruitment of aPKC to Par3 (or *vice versa*).

In conclusion, in our system, the interactions between aPKC and Par3 do not seem to be required for the assembly of the tripartite Par complex, and are natively insufficient to trigger assembly of the complex in the absence of binding to Par6. Interestingly, the phospho-independent Par3-PDZ2 to aPKC-PBM interaction has been established to be important in fly neuroblasts,^31^ while, in our mammalian fibroblasts, we found that it is quantitatively not required in the presence of assembly through Par6 (Figures S6D–S6G). This might reflect some differences in the Par complex assembly pathway between flies and mammals, and/or that Par complex assembly can be tuned depending on cell types. It must however be emphasized that our data does not preclude in any way the requirement of a direct Par3/aPKC interaction *after* Par complex assembly (via Par6). Our data only highlight the need for Par6 to bridge Par3 and aPKC to trigger initial *assembly* of the Par complex. Given the proposed ability of Par6 to inhibit aPKC activity,^26^ which our *in vivo* results on central spindle asymmetry are consistent with (Figures 6D and 6E), and the fact that the Par3-PDZ2/aPKC-PBM interaction is not phosphoregulated,^31^ it is totally possible that, once the initial assembly phase of the Par complex has occurred (via Par6), direct binding of aPKC and Par3 occurs and become important. Our data is broadly consistent with this model (as the full-length Par6 better recruits Par3 than the Par6 C-terminus alone, see Figures 3C and 3D), but further work will be needed to fully delineate how and when the aPKC-Par3 interaction arises.

##### Assembly does not depend on aPKC activity

As clustering aPKC can trigger assembly of the tripartite Par complex, we could test the effect of kinase activating (A129E) and inhibiting (K274W) mutations, which we found to not influence Par complex assembly (Figure S5). This is consistent with the fact that the Par3-aPKC interaction is dispensible for Par complex assembly, as clustering aPKC induces Par complex assembly through Par6, which is unaffected by aPKC phosphorylation, rather than through Par3, which *is* known to be affected by aPKC phosphorylation on one of its two binding sites. This is also consistent with the fact that abolishing aPKC-Par6 binding abolished tripartite Par complex assembly, irrespective of the kinase activity of aPKC (Figure S5J).

One caveat here is that, when we express the kinase-dead aPKC mutant, this does not abolish all aPKC activity in the cells, as the wild-type aPKC is still expressed. Hence, it is likely that Par3 remains (at least partially) phosphorylated in these cells, and hence, the assembly route involving direct interaction between Par3 and aPKC remains largely unavailable. However, we can conclude that the kinase activity of aPKC *within the Par clusters* does not affect Par complex assembly.

##### Par6 exists in an autoinhibited state

Given our demonstration that Par6 is the crucial component in Par complex assembly, as it is the intermediary between Par3 and aPKC, why then doesn’t Par6 bind to either Par3 or aPKC endogenously, when not clustered (Figures 2B, 2C, and S3)?

Clustering of WT Par6 leads to the rapid recruitment of aPKC, and delayed recruitment of Par3, and when Par6 cannot bind to aPKC (Par6A^ΔaPKC^ mutant), Par3 is not recruited to Par6 (Figures 2B–2D). As the aPKC-Par3 interaction cannot initiate Par complex assembly, aPKC binding to WT Par6 must change Par6 in some way, so as to allow it to bind to Par3. As discussed in above, this is unlikely to be due to a change in aPKC kinase activity. But how then is Par6 altered? The key insight came from clustering the C-terminus of Par6. The C-terminus contains the Par3 binding site, and lacks the aPKC binding site. It is therefore similar to the Par6A^ΔaPKC^ mutant, which also contains a Par3 binding site and lacks binding to aPKC. However, the two constructs behaved markedly differently: only the C-terminus alone of Par6 was able to recruit Par3 upon clustering (Figures 3C and 3D). This demonstrates that the N-terminus of Par6 can inhibit the binding of the C-terminus to Par3. It also suggests that aPKC binding to the N-terminus of Par6 can relieve this inhibition.

Consistent with Par6 existing in a directly autoinhibited state are the experiments shown in Figures 3F and S7, which demonstrate both in cells (with mitochondria relocalization) and *in vitro* that the N- and C-termini of Par6 can bind to one another. Further evidence comes from Figures 3C and 3E, where it is clear that the N-terminus of Par6 can recruit aPKC to a greater extent than the WT Par6, consistent with the C-terminus of WT Par6 partially inhibiting the aPKC binding of Par6. This is further evidenced by our Jo-Par6-In experiments, where the Jo and In domains partially force the N- and C-terminus of Par6 together, which strongly inhibits assembly of the tripartite Par complex (Figure 3D). Our model is also in agreement with AlphaFold2-predicted structures of Par6, which predicts an inter-domain interaction (Figure S7A).

We therefore conclude that Par6 natively exists in an autoinhibited state, where the N- and C-termini interact to restrict access to the aPKC and Par3 binding sites. Upon clustering of Par complex components, likely through an increase in local concentration and an enhanced avidity between components, this autoinhibition can be overcome by binding of aPKC or Par3 to Par6, which permits the binding of the remaining Par complex component to the free terminus of Par6.

##### Par6 is primarily “opened” by aPKC

In the previous section we discussed the evidence for Par6 existing in an autoinhibited state, which likely explains why the tripartite Par complex is natively largely unassembled in 3T3 cells. As aforementioned, clustering of any Par complex component is sufficient to induce Par complex assembly, likely by increasing the local concentration of Par complex components to a point where the affinity/avidity boost allows Par6 to be ‘opened’.

Two main lines of evidence suggest that aPKC is a more proficient opener of Par6 than Par3. Firstly, when Par6 itself is clustered, autoinhibition can only be relieved by aPKC (as Par6A^ΔaPKC^ cannot be ‘opened’ by Par3; Figures 2C and 2D), at least at the local density of Par6 we achieve with our synthetic clustering. Secondly, when clustering the N- or C-termini fragments of Par6, the N-terminus better recruits aPKC compared to the C-terminus recruiting Par3 (Figures 3C–3E), again consistent with the Par6-aPKC interaction being higher affinity than the Par6-Par3 interaction.

Importantly, while our data clearly point out that Par6 autoinhibition is a kinetic barrier to Par complex assembly, it is still slow (~1h) compared to the timescale of conformational changes. It is thus likely that the Par complex assembly pathway contains some yet unknown steps, perhaps via post-translational modifications for instance.

##### Assembly requires Par3 oligomerization

Previous work has demonstrated that Par3 oligomerizes *in vivo* through self-assembly of its N-terminal domain.^71,72^ Importantly, this oligomerization has been shown to be required for assembly of the Par complex *in vivo* in flies. Our data suggest that this oligomerization likely contributes to Par complex assembly by increasing the local concentration of Par3, allowing the ‘opening’ of Par6. When we clustered a Par3 mutant lacking this N-terminal domain at exactly the same density as the WT, its ability to ‘open’ Par6 and induce Par complex assembly was comparable to that of the WT Par3 (Figure S6G).

More recently, it has been proposed that Par3 forms condensates by phase separation.^43^ Combined with previous results, our data suggests that it is the high local concentration/density, rather than the specific biology and properties of biomolecular condensates (fluidity, high-density homotypic interactions, heterogenous molecular orientation, size heterogeneity, 3D geometry) that triggers Par complex assembly, as our crystalline, single layered, ordered 2D assembly could recapitulate this phenomenon (Figure S6). Furthermore, the artificial clustering of an N-terminal truncation of Par3, which lacks the oligomerization domain reported to trigger phase separation, leads to quantitative Par complex assembly to the same extent as the full-length (Figure S6G). This suggests that the clustering density achieved by our arrays is above the threshold for maximal Par complex assembly (our arrays fix the distance between Par complex subunit at ~8 nm. For comparison, this would correspond to a concentration of ~0.55 mM to reach the same intra-subunit distance in 3D). In other words, Par complex assembly, in our system, does not depend on the combination of synthetic Par complex clustering *and* phase separation of endogenous Par3 into these clusters, but solely on the local density of Par3 achieved by our clustering method. We conclude that, whether or not Par3 phase separates in a given system, Par complex assembly is driven by an increased local density of Par3, rather than by biophysical properties of a biomolecular condensate.

##### A unified model of Par complex assembly

From the conclusions detailed above, we propose a unified model for core Par complex assembly (Figure 3G). This presents the kinetically favored assembly route as a function of the subunit clustered.

Top row: Par6 exists natively in an unbound, autoinhibited state. Upon clustering, aPKC can bind and ‘open’ Par6, allowing the recruitment of Par3 to the C-terminus of Par6, which may be subsequently stabilized in the complex through the interactions with aPKC.

Middle row: aPKC exists natively in an unbound state, but upon clustering, independent of its kinase activity, aPKC can bind to Par6 and relieve its autoinhibition. This allows the recruitment of Par3 to the ‘opened’ C-terminus of Par6, and the subsequent stabilizing of Par3 into the complex if Par3 undergoes dephosphorylation.

Bottom row: Par3 natively exists in an unbound state, but, upon clustering, it can partially ‘open’ Par6. This allows the subsequent recruitment of aPKC into the complex, which, if Par3 is dephosphorylated, can be further stabilized through its interaction with Par3.

##### Relevance of these findings to in vivo Par complex biology

The work presented here highlights how synthetic biology can increase our understanding of cell biology by interrogating biological systems in an orthogonal way. The key finding that the rate-limiting step in the assembly of the Par complex is the ‘opening’ of Par6 potentially resolves multiple discrepancies between different systems. In some systems,^30,71,73^ the Par complex is natively assembled, and this assembly depends on the N-terminal oligomerization of Par3. This contrasts with other systems,^24,25^ and our findings in 3T3 cells, where, despite the expression of Par3 containing an N-terminal oligomerization domain, the Par complex is not assembled. We propose that, in 3T3 cells, the local concentration of Par3, even despite oligomerisation/condensation, is insufficient to induce ‘opening’ of Par6, unlike in Drosophila, where oligomerized (but not monomeric) Par3 can ‘open’ Par6, likely due to a higher relative local concentration. The potential stabilizing effect of the Par3-aPKC interaction could amplify the difference between these systems, as, once the threshold for Par6 ‘opening’ has been met, the assembled Par complex could be further stabilized. In line with Par6 ‘opening’ depending on the local concentration of Par complex components, several studies where Par complex components have been overexpressed have demonstrated that this is sufficient to induce Par complex assembly.^14,43^

It is interesting to note that aPKC is seemingly more efficient at ‘opening’ Par6 than Par3, when it is Par3 that is known, *in vivo*, to oligomerize. It will be interesting to assess what relevance this finding has *in vivo*, and whether cases exist where Par complex assembly is initiated *in vivo* through clustering of aPKC.

Finally, it has been suggested that, in some systems, Par6 and aPKC form a stable subcomplex.^70^ Why then, if aPKC is bound to and has ‘opened’ Par6, does the triple complex not constitutively form in all these cases? Relevant to this question is the observation that the clustered C-terminus of Par6 alone only modestly colocalizes with Par3, compared to clustering of the full-length Par6 (and associated aPKC). Likely, the off-rate of Par3 from the C-terminus of Par6 is high, unless stabilied by the interaction with aPKC. This stabilizing interaction, which is known to be regulated by the kinase activity of aPKC and the phosphorylation state of Par3, likely determines whether a Par6-aPKC subcomplex stably binds Par3, or binds/unbinds Par3 reversibly. This will further be regulated by the concentration of components known to assemble Par complex components into different complexes (such as Cdc42), which will alter the local concentration of ‘free’ Par complex components competent to assemble the Par complex. While we did not detect colocalization between Par complex components and Cdc42 in our system (data not shown), this could affect Par complex assembly in other systems.

### Quantification And Statistical Analysis

Unless stated otherwise, measurements are given as mean ± SEM. Statistical analyses were performed using GraphPad Prism 8 with an alpha of 0.05. Normality of variables was verified with Kolmogorov-Smirnov tests. Homoscedasticity of variables was always verified when conducting parametric tests. Post-hoc tests and their respective p-values are indicated in their respective figure legends.

## Supplementary Material

Fig S1

Fig S2

Fig S3

Fig S4

Fig S5

Fig S6

Fig S7

Fig S8

Fig S9

Fig S10

Graphical Abstract

Vid S1

Vid S2

Vid S3

Vid S4

Vid S5

Vid S6

Vid S7

Vid S8

Vid S9

Vid S10

Vid S11

Vid S12

Supplementary Figures Legends

## Figures and Tables

**Table 1:** Comparison of the different available methods to reconstitute polarity to study asymmetric cell division

Method (original paper)	Basis of the ectopic polarity	Works on endogenous or exogenous targets	Inducible	Reversible	Clustering speed	Works in mitosis
Johnston et al.^12^	cell-cell junction	exogenous	no	no	slow (minutes)	yes
Okumura et al.^15^	patterned optogenetics inducing oligomerization	exogenous	yes	yes	fast (seconds)	yes
Habib et al.^11^	single Wnt-bound bead binding to cell	endogenous proteins; identity of the relocalized protein (Wnt receptors) cannot be changed	yes	no	slow (minutes)	yes
Kono et al.^14^	self-oligomerization of expressed Par complex	exogenous	no	no	slow (minutes)	yes
Théry et al.^17^	micropatterning of adhesion molecules imposing a polarized shape onto cells	endogenous proteins; no control over the identity of the relocalized protein (adhesion molecules)	yes	possible	slow (minutes)	yes
Watson, Krüger et al. (this study)	synthetic polymer with expressed synthetic anchor	exogenous or endogenous (polymer can be functionalized with ligands)	yes	no	fast (seconds)	yes
Method (original paper)	Works in interphase	Works with single cells	Cell type used	High- throughput	Demonstrated use relevant to this paper		
Johnston et al.^12^	yes	no	*Drosophila* S2, human HeLa	yes	mechanisms of mitotic spindle orientation in *Drosophila*^12^ and mammalian cells^13^		
Okumura et al.^15^	yes	yes	human HeLa	no	mechanisms of mitotic spindle orientation in mammals		
Habib et al.^11^	yes	yes	murine ES	yes (presumably)	mechanisms of mitotic spindle orientation in mammals		
Kono et al.^14^	no	yes	*Drosophila* ES	yes (presumably)	mechanisms of Par complex assembly in interphase in insects		
Théry et al.^17^	yes	yes	most cell types	yes	investigation of the physical basis of cell polarity (Thery et al.^17^) and mechanisms of mitotic spindle orientation controlled by cell shape^17^		
Watson, Krüger et al. (this study)	no	yes	murine 3T3 and ES, human U2OS, *Drosophila* S2	yes	mechanisms of mitotic spindle orientation, central spindle symmetry breaking and Par complex assembly in mammals		

**Table 2:** List of constructs of Par subunit mutants used in this study

Name in this study	Construct details	Comment
Par6A	Par6A	Par6A wild type
Par6B	Par6B	Par6B wild type
Par6G	Par6G	Par6G wild type
Par6A^ΔaPKC^	Par6A^K19A^	Par6A mutant lacking direct aPKC-binding
Par6A^Nter^	Par6^1–121^	Par6A N terminus (containing the PB1 domain)
Par6^Cter^	Par6A^121–346^	Par6A C terminus (containing the Cdc42- and Rac-interactive binding motif [CRIB], PDZ, and disordered domains)
Par6A^247-346^	Par6A^247–346^	Par6A C-terminal disordered domain
Par6A^121-346AAA^	PAR6A^121–346^ with LGF^169–171^ mutated to Alanine	Par6A C terminus (CRIB/PDZ and disordered domains, with PDZ motif mutated)
Jo-Par6A-In	Jo-Par6A-In	Par6A fused to self-covalently binding Jo/In-fragments
aPKC	aPKC_ι_	aPKC_ι_ wild type (for simplicity, aPKC_ι_ will be refered to as aPKC in this study)
aPKC^ΔPar6^	aPK_ι_^E85A/R91A^	aPKC mutant lacking Par6-binding
aPKC^dead^	aPKC_ι_^K274W^	aPKC mutant kinase dead
aPKC^active^	aPKC_ι_^A129E^	aPKC mutant kinase active
aPKC^dead-ΔPar6^	aPKC_ι_^K274W/E85A/R91A^	aPKC mutant kinase dead + lacking direct Par6-binding
aPKC^active-ΔPar6^	aPKC_ι_^A129E/E85A/R91A^	aPKC mutant kinase active + lacking direct Par6-binding
aPKC^ΔPBM^	aPKCι^Δ857–862^	aPKC mutant lacking the direct Par3-binding PBM domain
Par3	Par3^180 kD^	Par3 wild type full-length
Par3^ΔaPKC1^	Par3^100 kD^	Par3 isoform lacking phospho-regulated direct aPKC-binding site but containing the second aPKC-binding site
Par3^ΔN^	Par383^–1319^	Par3 deleted for the N-terminal oligomerization domain
Par3^SA^	Par3^S824A/S826A^	Par3 full-length, phospho-inhibit mutation on phospho-regulated direct aPKC-binding sites (more aPKC binding)
Par3^SD^	Par3^S824D/S826D^	Par3 full-length, phosphomimetic mutation on phospho-regulated aPKC-binding site (less aPKC binding). Note that the second aPKC-binding site (PDZ2) is still present
Par3^ΔaPKC1-ΔN^	Par3^100 kD Δ1–83^	Par3 isoform lacking direct aPKC binding, the phosopho-regulated direct aPKC-binding site, and the N-terminal oligomerization domain. Note that the second aPKC-binding site (PDZ2) is still present
Par3^ΔaPKC1-ΔPDZ2-ΔN^	Par3^100 kD Δ1–83 Δ728–813^	Par3 isoform lacking both direct aPKC-binding sites (phospho-regulated and PDZ2), as well as the N-terminal oligomerization domain
Par3^ΔaPKC1-ΔPDZ2-ΔPar6-ΔN^	Par3^100 kD G600/602A Δ1–83 Δ728–813^	Par3 isoform lacking both aPKC-binding sites (phospho-regulated and PDZ2), as well as N-terminal oligomerization domain and direct Par6-binding site

## Data Availability

All data reported in this paper will be shared by the lead contact upon request. AlphaFold2 structure prediction of Par6A originated from the Alphafold protein structure database (https://alphafold.ebi.ac.uk/entry/Q9NPB6). All original code, including code for quantifying colocalization in 3D, has been deposited on our GitHub page and is publicly available as of the date of publication. DOIs are listed in the key resources table. Any additional information required to reanalyze the data reported in this paper is available from the lead contact upon request.

## Highlights

- Designed proteins allow induction of cortical polarity of any protein during mitosis
- Par complex assembly driven by subunit clustering requires Par6 autoinhibition relief
- Synthetic cortical Par caps induce spindle orientation and central spindle asymmetry
- Central spindle asymmetry is evolutionary conserved and induced by aPKC kinase activity

## In brief

Synthetic engineering of cortical polarity using designed proteins reveals that reconstituting Par complex polarity is sufficient to drive key processes of asymmetric cell division in unpolarized mammalian cells.
