# Supplementary figures and images for: Synthetic Par polarity induces cytoskeleton asymmetry in unpolarized mammalian cells
Source: Cell. Author manuscript; Available in PMC 2024 Feb 7. (PMC10765089; doi:10.1016/j.cell.2023.08.034)

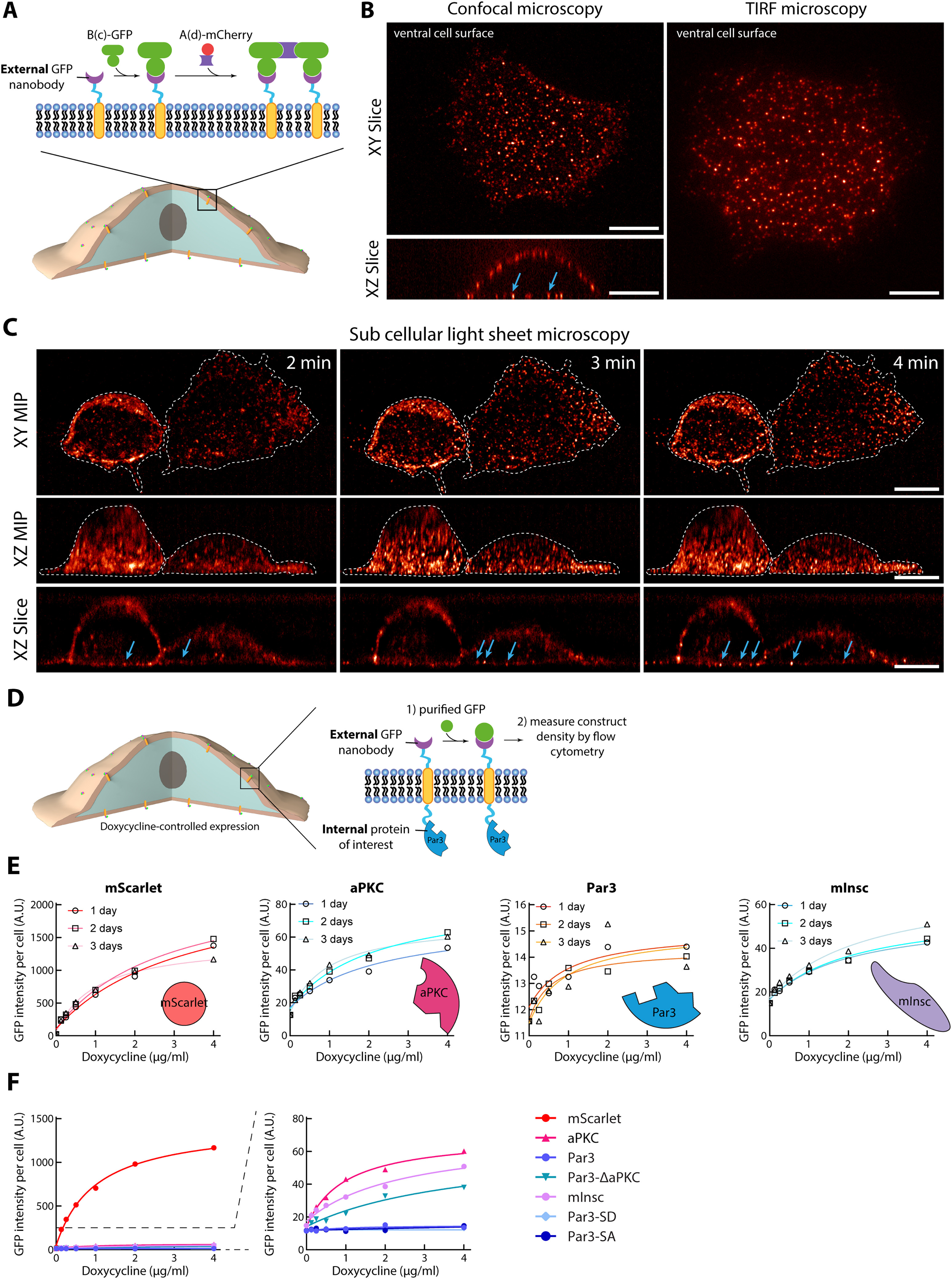

Supplement: Fig S1 [file EMS192825-supplement-Fig_S1.jpg]

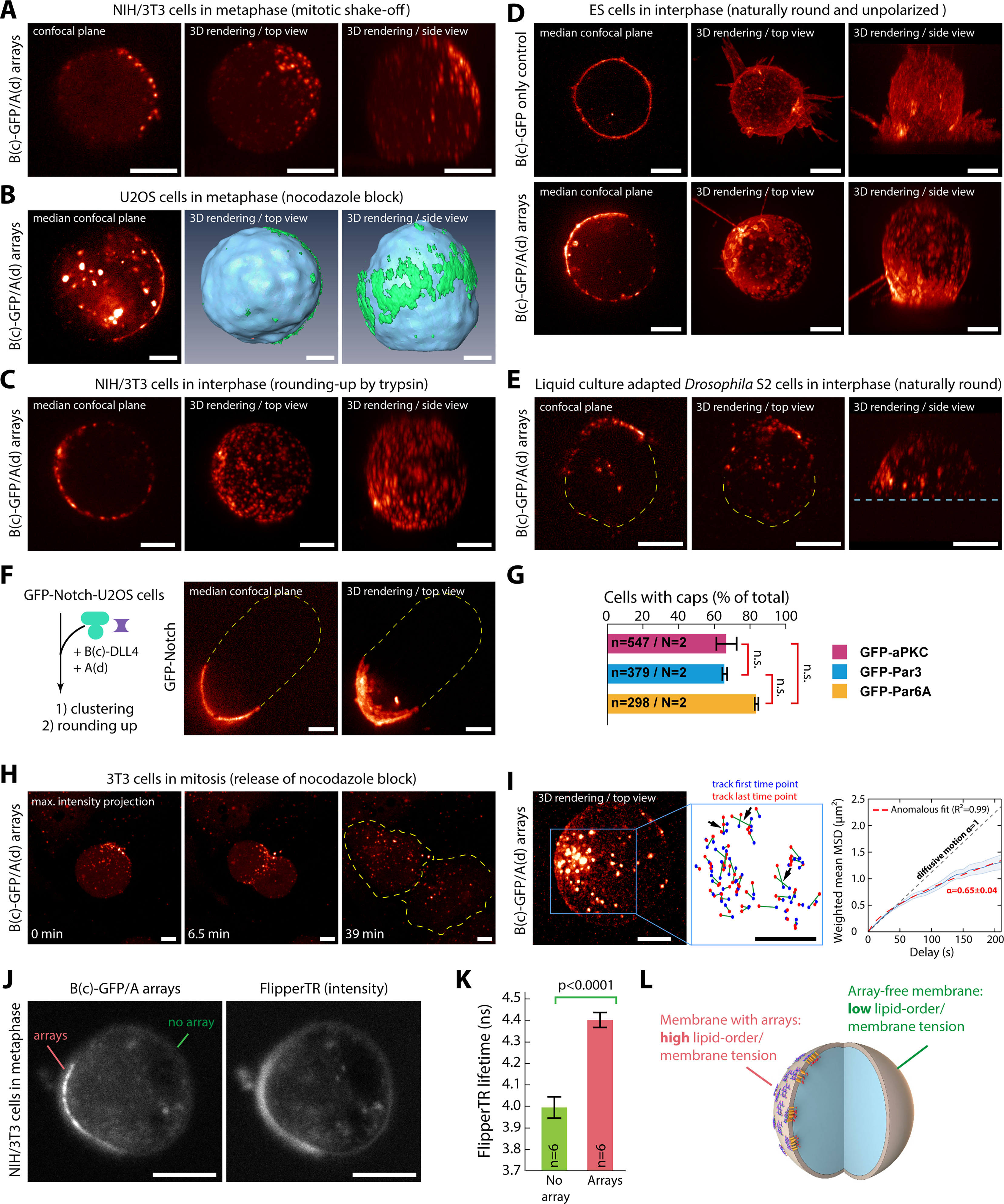

Supplement: Fig S2 [file EMS192825-supplement-Fig_S2.jpg]

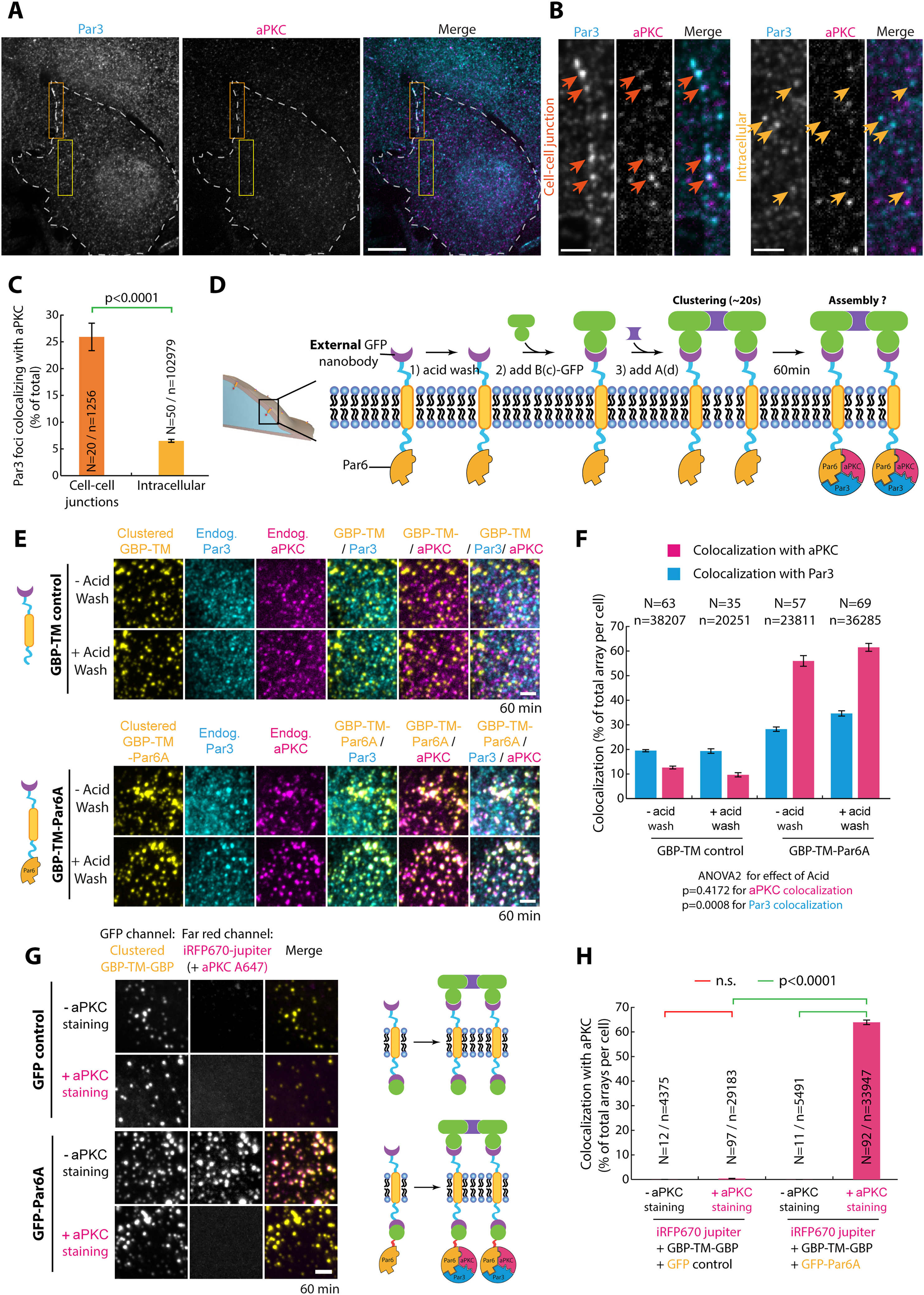

Supplement: Fig S3 [file EMS192825-supplement-Fig_S3.jpg]

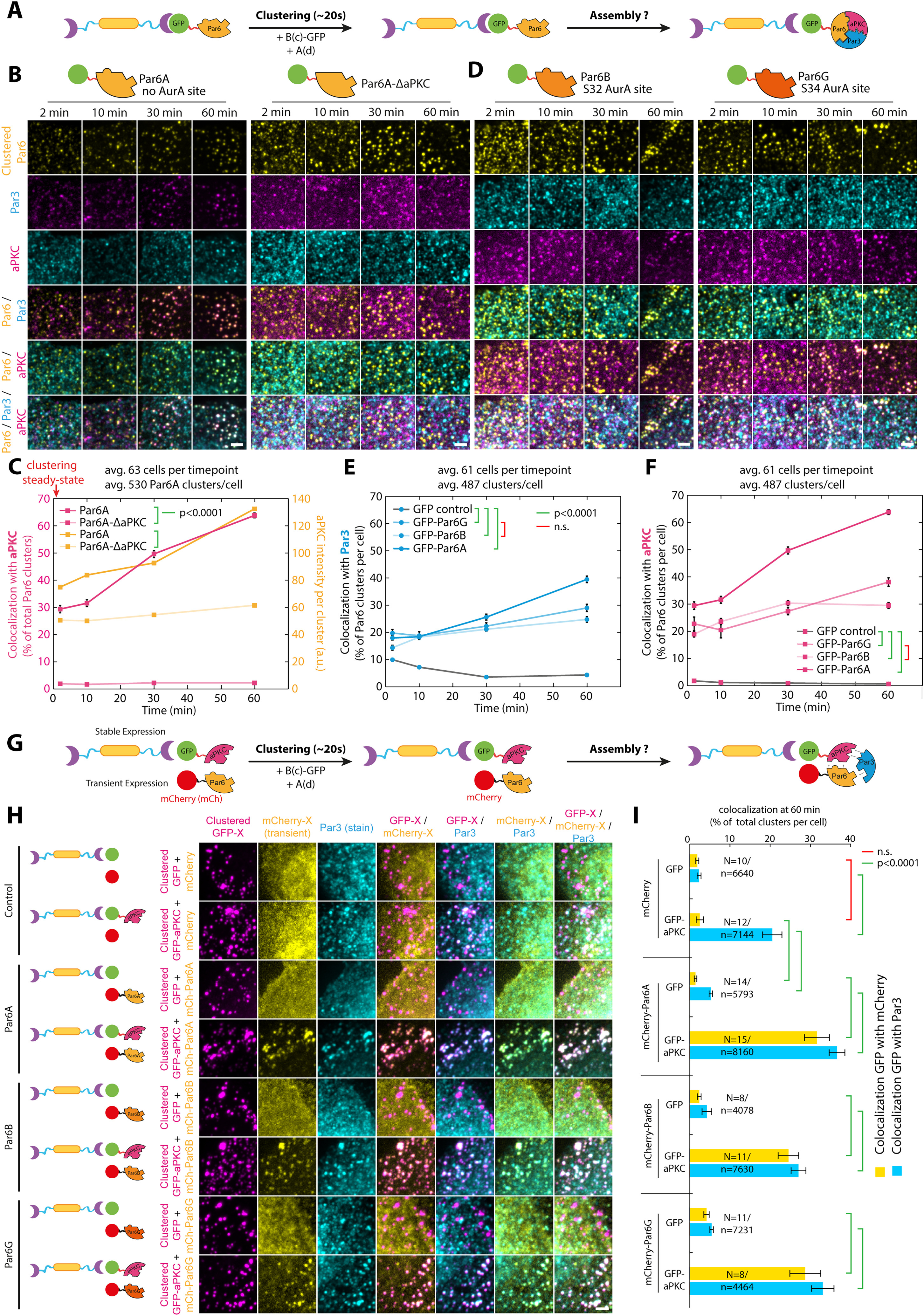

Supplement: Fig S4 [file EMS192825-supplement-Fig_S4.jpg]

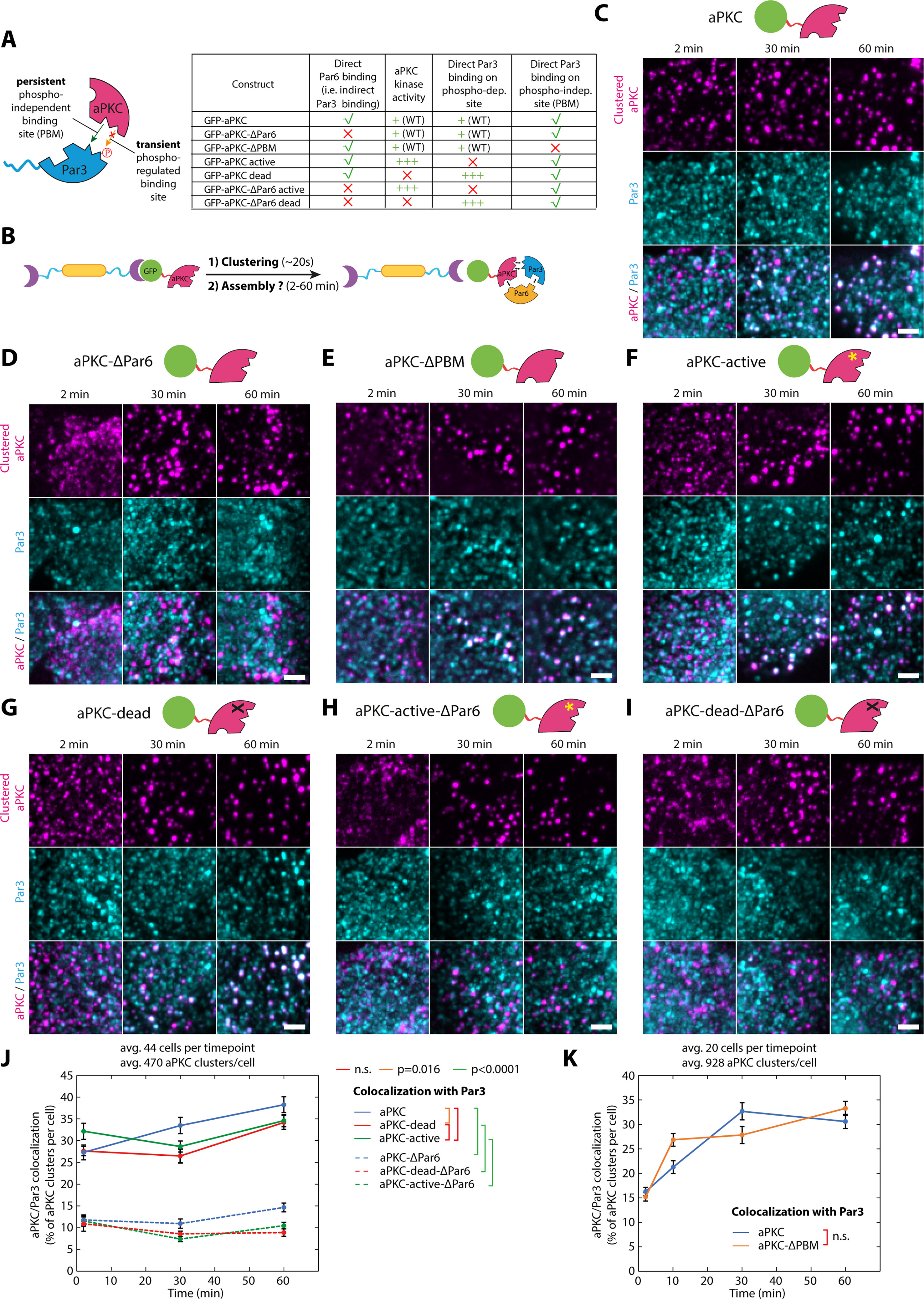

Supplement: Fig S5 [file EMS192825-supplement-Fig_S5.jpg]

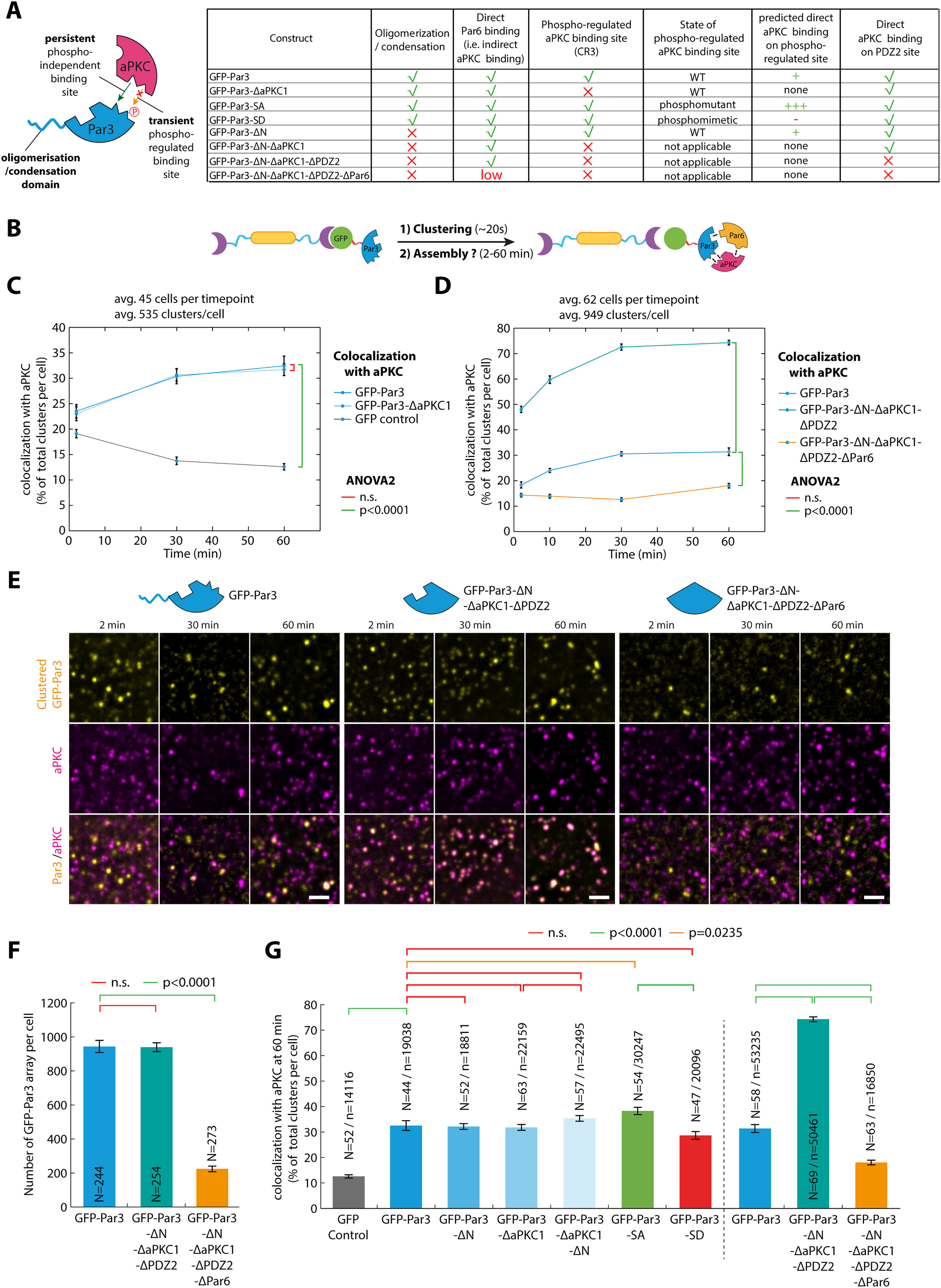

Supplement: Fig S6 [file EMS192825-supplement-Fig_S6.jpg]

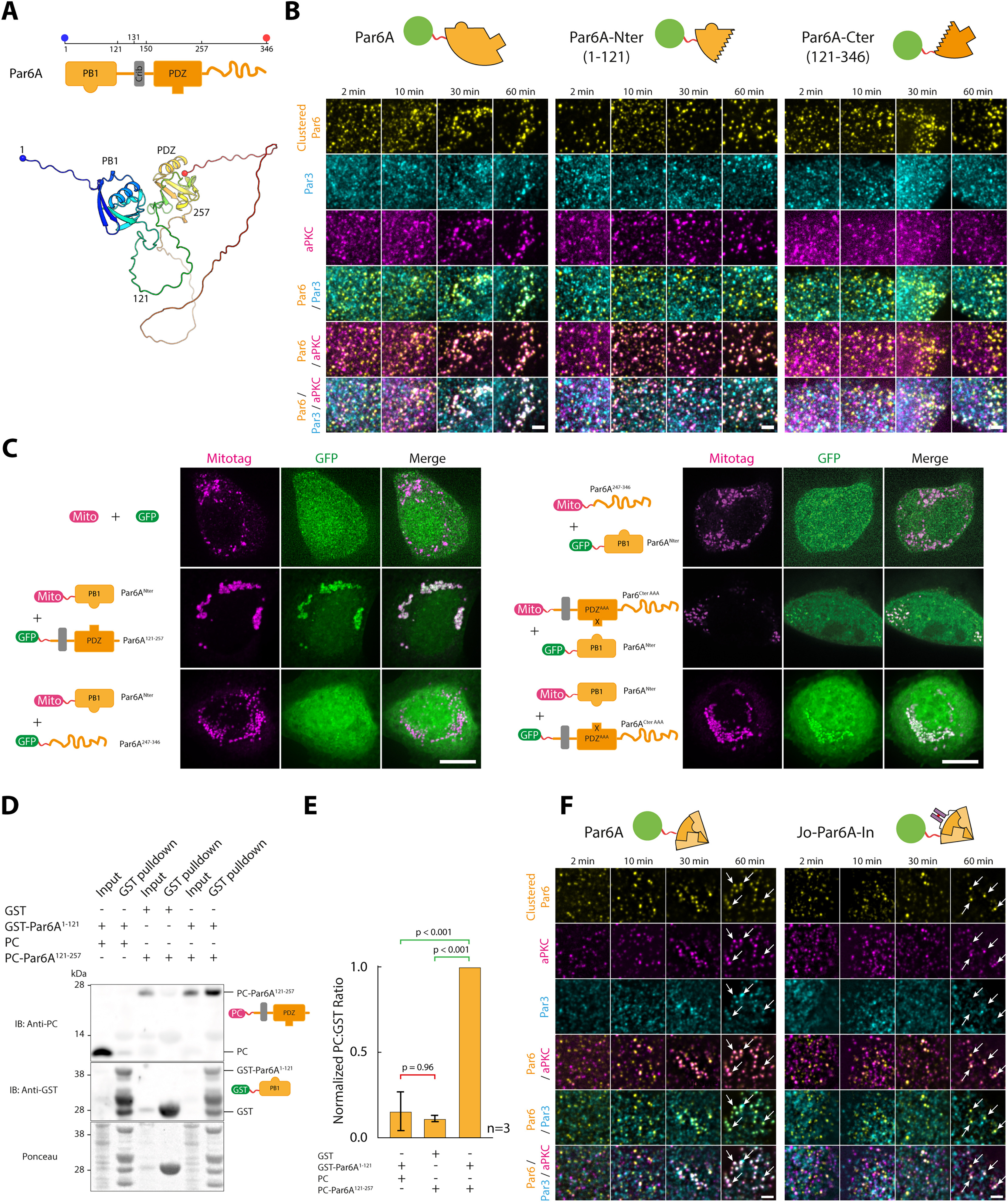

Supplement: Fig S7 [file EMS192825-supplement-Fig_S7.jpg]

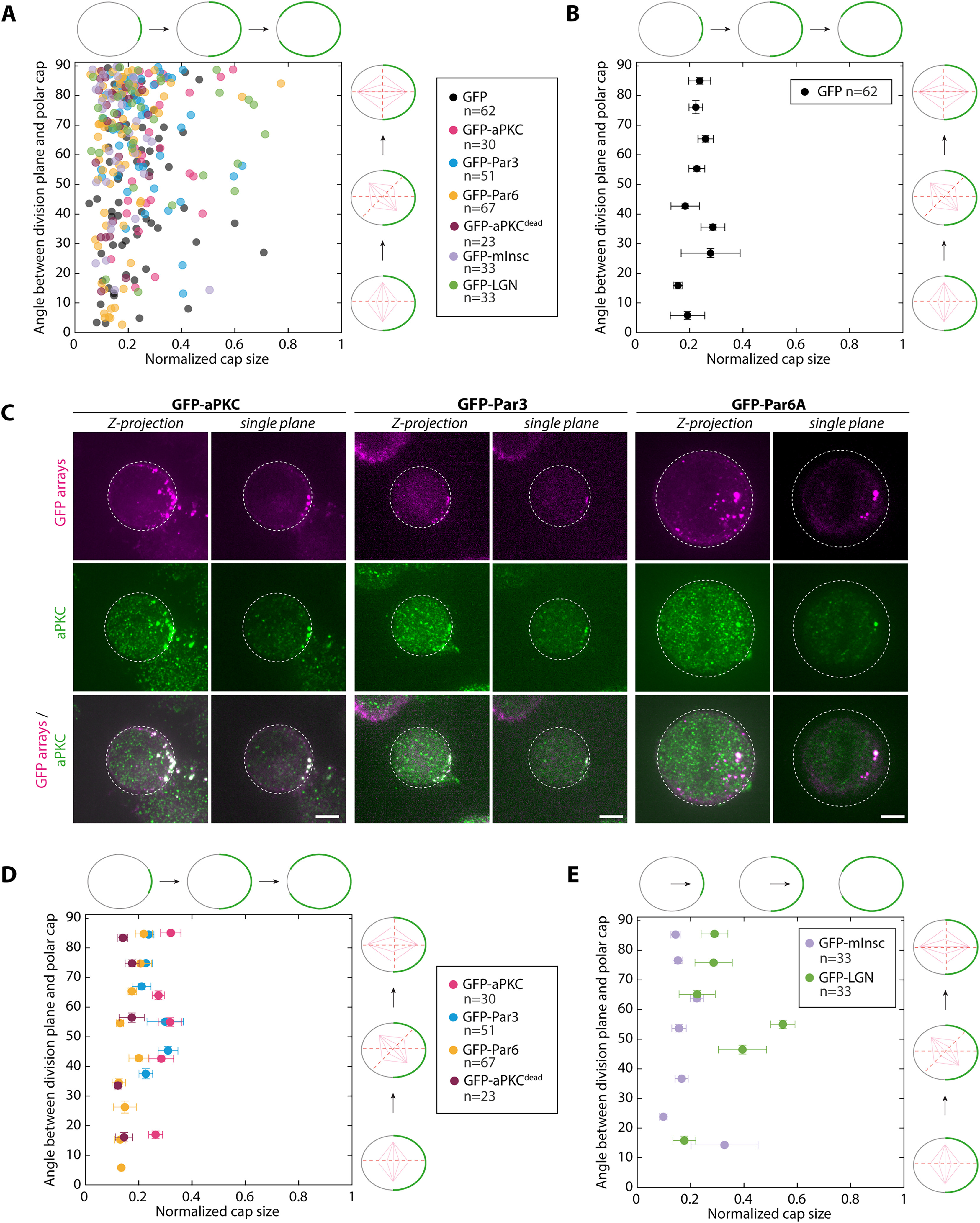

Supplement: Fig S8 [file EMS192825-supplement-Fig_S8.jpg]

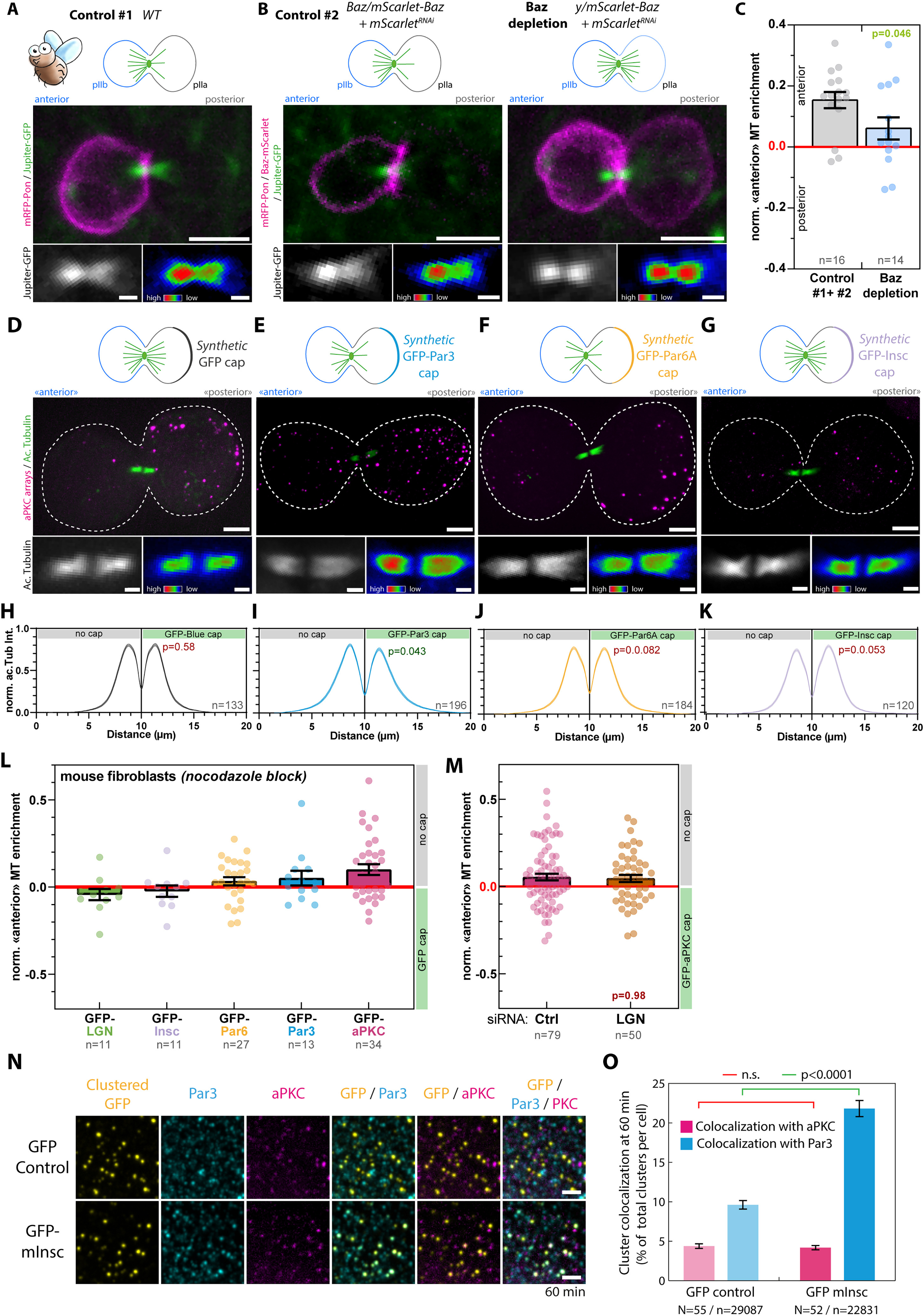

Supplement: Fig S9 [file EMS192825-supplement-Fig_S9.jpg]

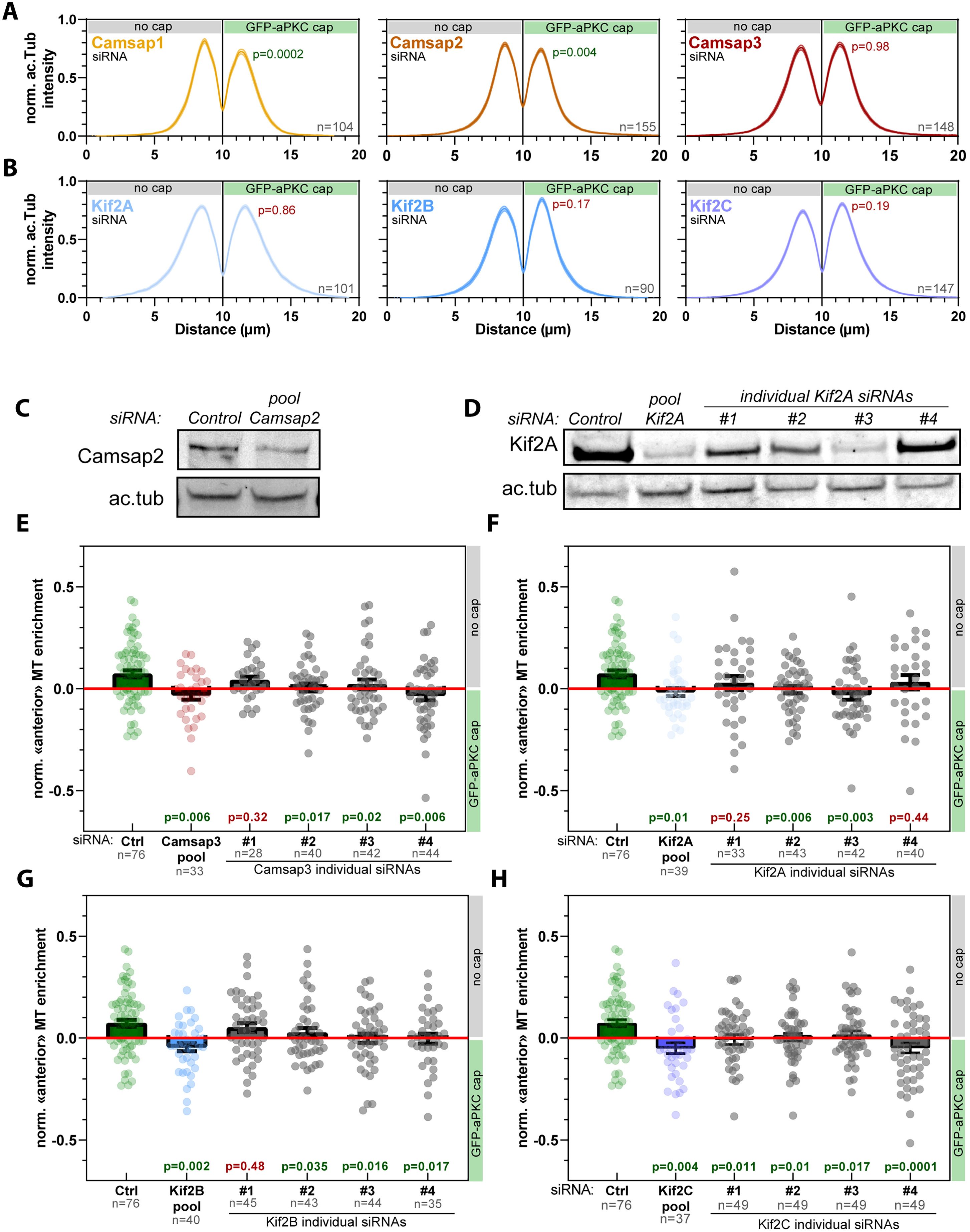

Supplement: Fig S10 [file EMS192825-supplement-Fig_S10.jpg]

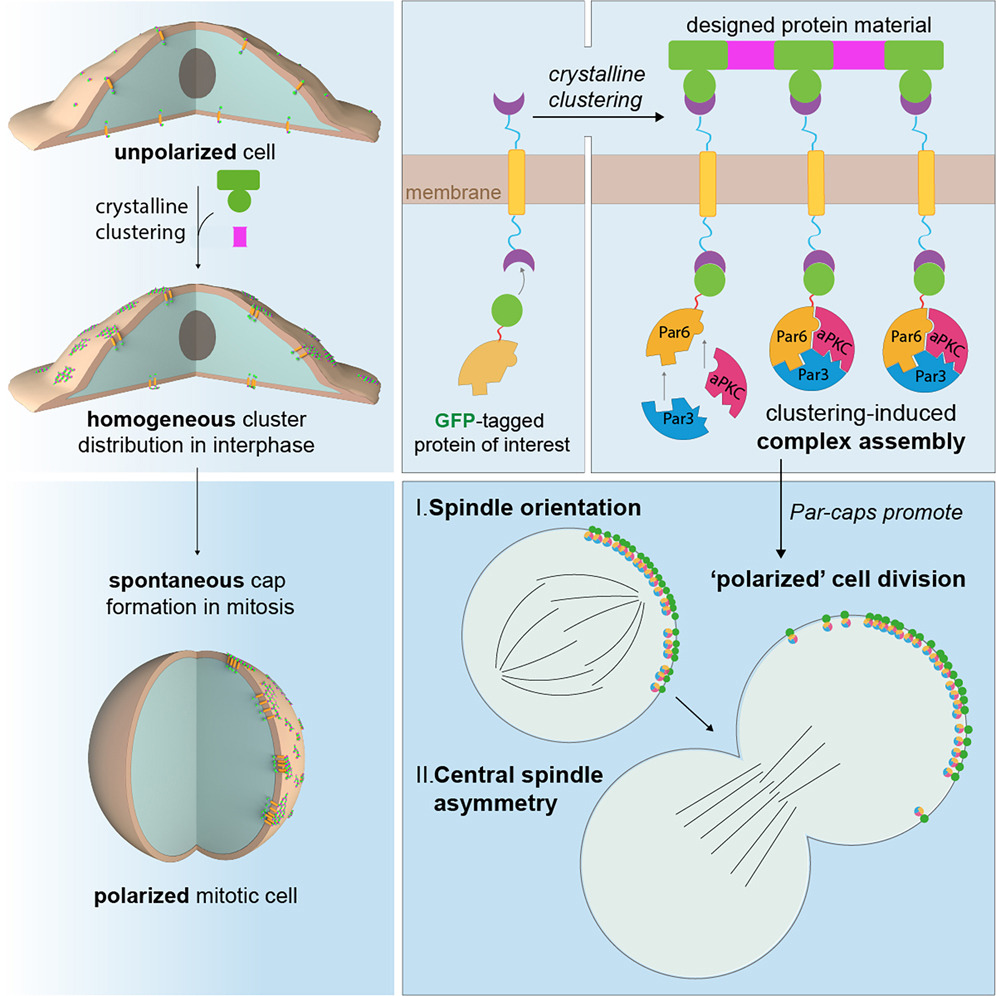

Supplement: Graphical Abstract [file EMS192825-supplement-Graphical_Abstract.jpg]
