# Supplementary material for: Synthetic Par polarity induces cytoskeleton asymmetry in unpolarized mammalian cells
Source: Cell. Author manuscript; Available in PMC 2024 Feb 7. (PMC10765089; doi:10.1016/j.cell.2023.08.034)
Supplement: Supplementary Figures Legends [file EMS192825-supplement-Supplementary_Figures_Legends.pdf]

CellPress
OPEN ACCESS

# Supplemental figures

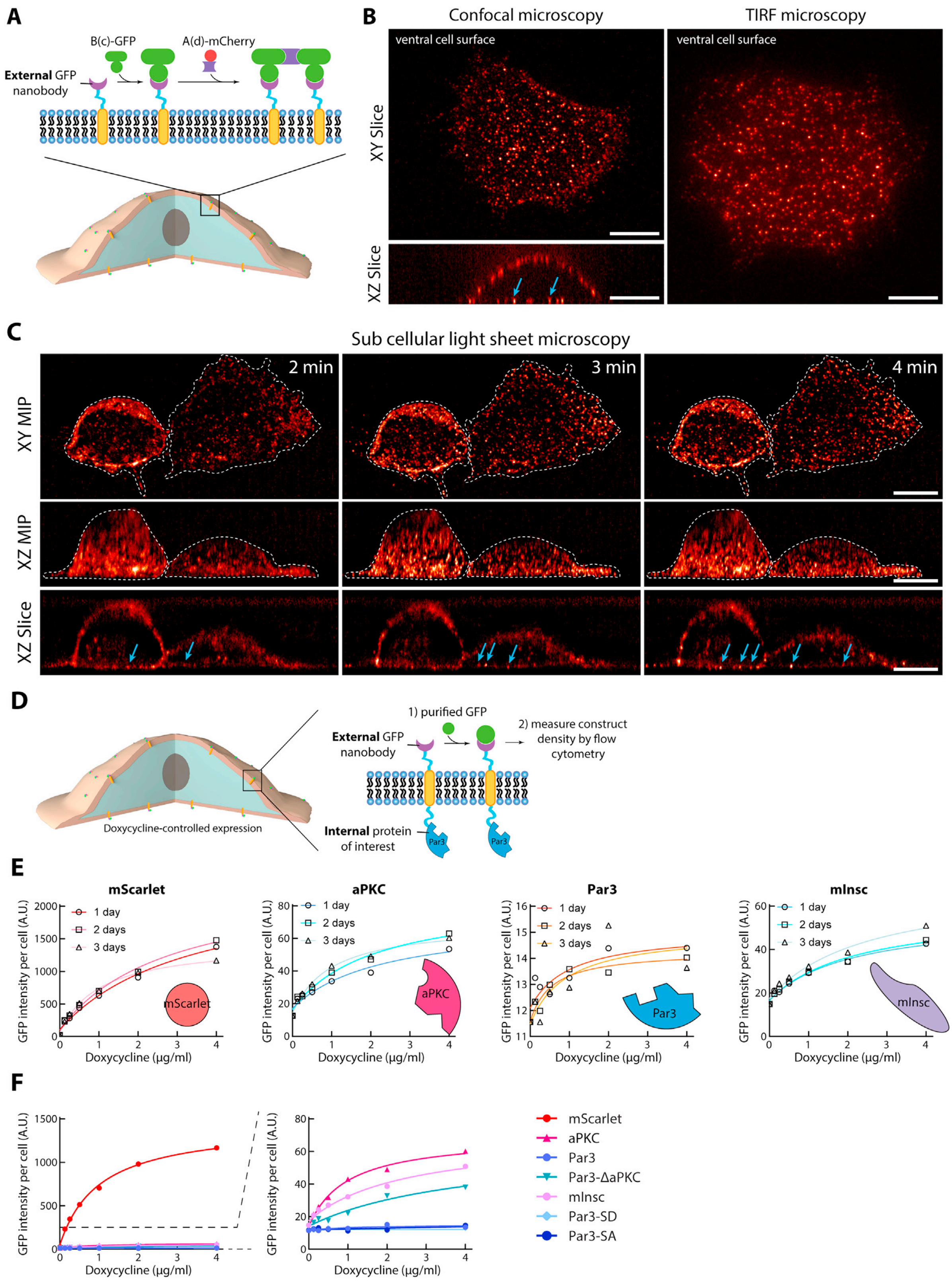


(legend on next page)

**Figure S1. Characterization of volumetric clustering dynamics and effect of fused protein on construct density, related to Figure 1**

(A) Principle of the experiment: 3T3 cells stably expressing GBP-TM were incubated with B(c)-GFP, then A(d)-mCherry to induce rapid clustering.

(B) Cells treated as in (A) were imaged by spinning disk confocal microscopy (left, SDCM) or total internal reflection fluorescence microscopy (right, TIRF). For confocal, images correspond to one optical slice through the ventral surface (top) or XZ slice through the volume of the cell (bottom).

(C) 3T3 cells stably expressing GBP-TM-mScarlet were treated as in (A) with B(c)-GFP, then unlabeled A(d), and GFP-fluorescence was imaged by subcellular light sheet microscopy (see also Video S1). Images correspond to maximum intensity z-projections (MIPs, top and middle), or XZ optical slice (bottom).

(D) Principle of the experiment: 3T3 cells expressing GBP-TM fused to a protein of interest under the control of a doxycycline inducible promoter were treated with doxycycline for the indicated time, before incubation with purified GFP. The GFP signal per cell was measured by flow cytometry as a proxy of the density of the transmembrane construct at the surface of cells expressing the indicated fusion.

(E) GFP intensity per cell expressing the indicated fusion was treated with doxycycline for 3 days and processed as above.

(F) Samples presented in (E) were plotted onto the same graph (3-day samples). Scale bars, 10 μm.

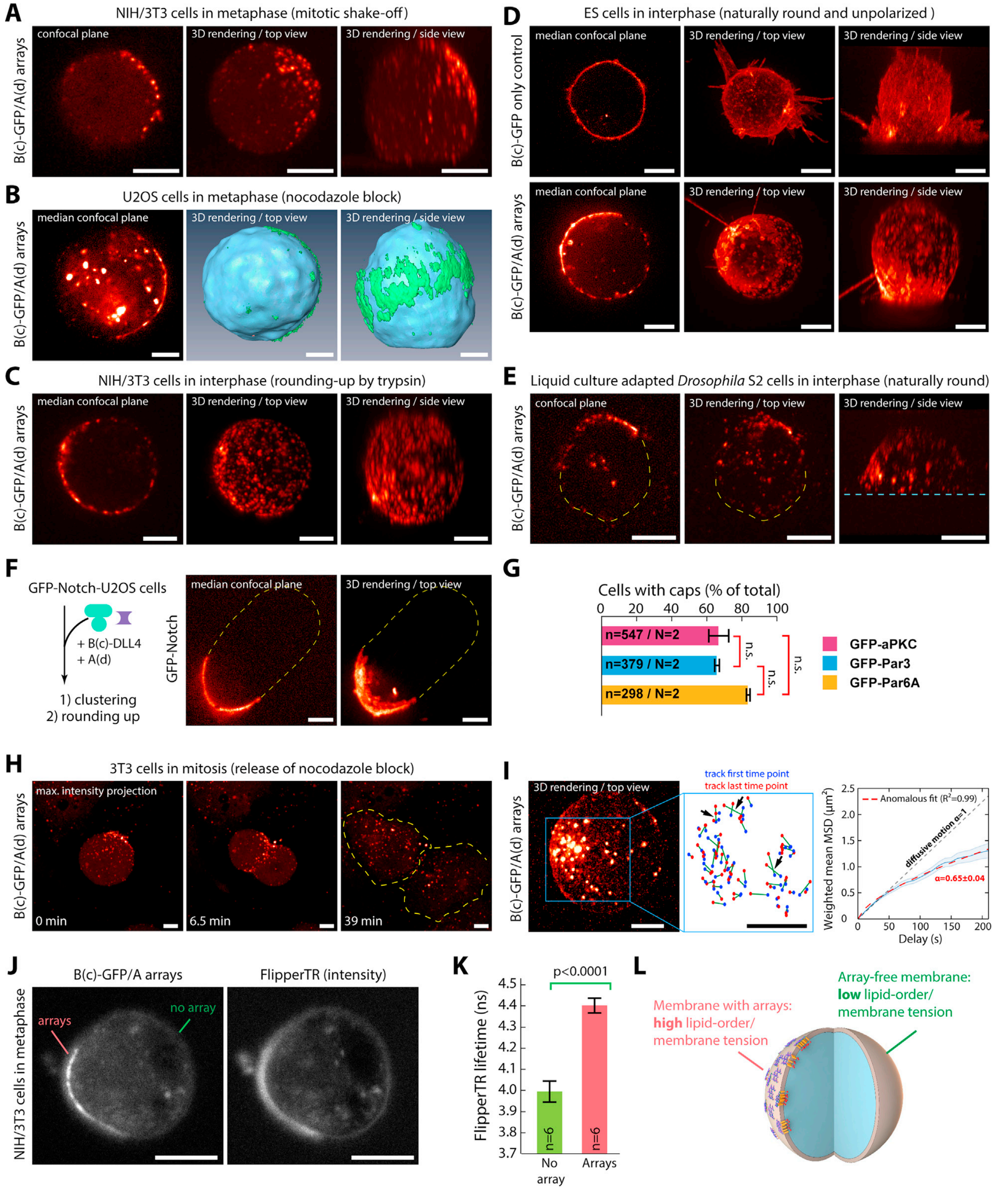


**Figure S2. Induction of cap formation in different rounded cell types, related to Figure 1**

(A–C) SDCM images after sequential incubation with B(c)GFP and A(d) for array formation of rounded-up mitotic 3T3 cells co-expressing GBP-TM-GBP and GFP (A), U2OS cells transiently expressing GBP-TM-mScarlet arrested in mitosis using nocodazole block (B), and 3T3 cells expressing GBP-TM-mScarlet in interphase with cell-rounding induced using trypsin (C).

(legend continued on next page)

(D) Mouse embryonic stem cells stably expressing GBP-TM-GBP imaged by SDCM after incubation with B(c)GFP followed by A(d) to induce array formation (bottom) or not (top).
(E) Suspension *Drosophila* S2 cells expressing GBP-TM-GBP after incubation with B(c)GFP followed by A(d) for array formation. SDCM imaging.
(F) Cap formation is not restricted to artificial GBP-TM constructs. SDCM images of arrays assembled at the surface of U2OS cells expressing GFP-Notch by sequential incubation with B(c)-SC:ST-DLL4 (DLL4 is a Notch ligand) and A(d)-GFP.
(G) Efficiency of cap formation, assessed by the percentage of cells with an asymmetric cap after mitotic shake-off, does not significantly depend on the protein of interest targeted to the cap in 3T3 cells co-expressing GBP-TM-GBP and indicated GFP fusion. (Mean ± SD; n = total number of cells scored indicated in each condition; N = number of independent experiments.) Statistics: Kruskal-Wallis test.
(H) Caps are reversible and dissolve upon spreading for adherent cells. Caps were induced at the surface of 3T3 cells co-expressing GBP-TM-GBP and GFP as in (A) followed by stalling in mitosis with 30 nM nocodazole for 12 h. The arrays were then imaged by SDCM upon release of the nocodazole block.
(I) Caps are not formed by a reticulated network of arrays. Caps were induced at the surface of 3T3 cells co-expressing GBP-TM-GBP and GFP-aPKC as in (A), and cells were then stalled in mitosis with 30 nM nocodazole for 12 h. Cells were then imaged by oblique plane light sheet microscopy (left), and arrays were tracked in 3D. Middle: distance traveled by each track plotted as the first (blue dot) and last (red dot) time point. Black arrows indicate events of crossing between tracks marking events when arrays change neighbors. Right: mean square displacement (MSD) analysis of array motion as a function of delay time. The thick blue line corresponds to the weighted mean curve, which weights the MSD curves according to their certainty (lighter area, SEM). Red line: anomalous fit of the weighted mean curve. N = 137 tracks.
(J–L) Array localization correlates with membranes of altered biophysical properties.
(J) 3T3 cells stably expressing GBP-TM-mScarlet with assembled arrays and stalled in mitosis using nocodazole incubated with Flipper-TR probe processed for fluorescence lifetime imaging.
(K) Intensity-weighted average lifetime of the Flipper-TR probe in regions where arrays are present (segmented using GFP fluorescence) or not (mean ± SEM). A higher Flipper-TR lifetime of the probe indicates a local high-order and/or high-tension in the membrane. Statistics: paired Student's t test (p value indicated; n: number of cells analyzed).
(L) Summary: arrays correlate with regions of higher membrane tension and/or higher lipid packing compared with the surrounding naked membrane. Images in this figure correspond to single confocal planes, 3D reconstruction, or 3D reconstruction with surface rendering as indicated (see STAR Methods).
When necessary, cell contours are indicated in yellow dashed lines and the coverslip in blue dashed lines. Scale bars, 5 μm.

CellPress
OPEN ACCESS

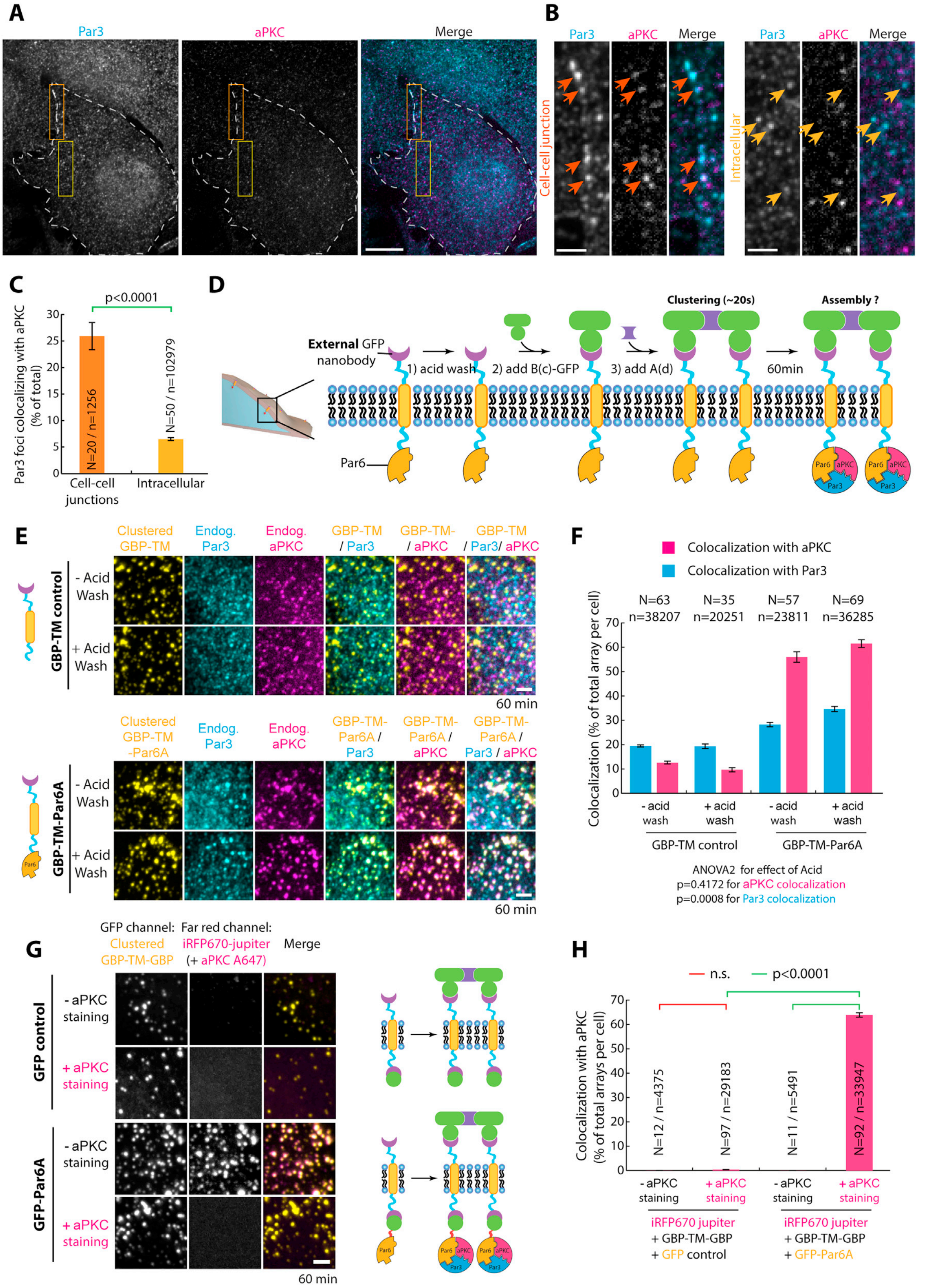


(legend on next page)

**Figure S3. Determination of physiological Par complex assembly at cell-cell junctions in 3T3 cells and control experiments of the bicistronic clustering method, related to Figure 2**

(A) 3T3 cells in interphase immunostained for endogenous Par3 and aPKC and imaged by SDCM. Images correspond to MIPs of 7 planes ($\Delta z$ = 1.4 μm total). Dashed white line: cell contours.

(B) High magnification of the image presented in (A) in a cell-cell junction region (left, corresponds to orange rectangle in (A) or in an intracellular region (right, corresponds to yellow rectangle in A). Arrows indicate Par3 clusters colocalizing with aPKC at cell-cell junctions in contrast to the rest of the cell.

(C) Mean ± SEM percentage of colocalization between Par3 clusters and aPKC per region (see also STAR Methods for details about automated 3D object-based colocalization). Statistics: unpaired Student's t test (N: regions of interest analyzed; n: total number of Par3 spots detected).

(D–F) The acid wash treatment ensuring that the external GBP is not bound to GFP does not affect the colocalization between Par6A and aPKC/Par3 used in this study.

(D) Principle of the experiment: Cells are treated with a quick acid wash prior to GBP-TM-GBP clustering to avoid extracellular GBP from being saturated with GFP fusion proteins in the culture medium (released upon cell death, for instance).

(E) Cells processed as in (D) were immunostained for endogenous aPKC and Par3 after 60 min of clustering. Imaging was performed by SDCM (MIPs: $\Delta z$ = 3.8–4.6 μm total).

(F) Mean ± SEM percentage of the 3D colocalization between GFP-Par6A (or GFP control) clusters and aPKC or Par3 per cell. Effect of the acid wash was tested using an ANOVA2 test (respective p value indicated).

(G and H) The weak iRFP670-Jupiter signal in the far-red channel does not affect the accuracy of the detection the strong signal of the Alexa-647 aPKC immunostaining.

(G) Cluster formed at the surface of 3T3 cells expressing iRFP670-Jupiter, GBP-TM-GBP, and GFP-Par6A (or GFP control) were immunostained (or not) for endogenous aPKC using Alexa647-coupled secondary antibodies and imaged by SDCM (MIPs; $\Delta z$ = 3.4–4.6 μm total). Dynamic range was set to be identical between images.

(H) Mean ± SEM percentage of the 3D colocalization between Par3 clusters and aPKC per cell. Statistics: Kruskal-Wallis test followed by a Dunn post hoc test (p value of respective tests indicated).

In (F) and (H), N corresponds to the number of cells analyzed per condition, and n corresponds to the total number of GFP-positive arrays detected per condition. All images in this figure were processed with a wavelet "a trous" filter (see STAR Methods). Scale bars: 10 μm in (A) and 2 μm in (B), (E), and (G).

CellPress
OPEN ACCESS

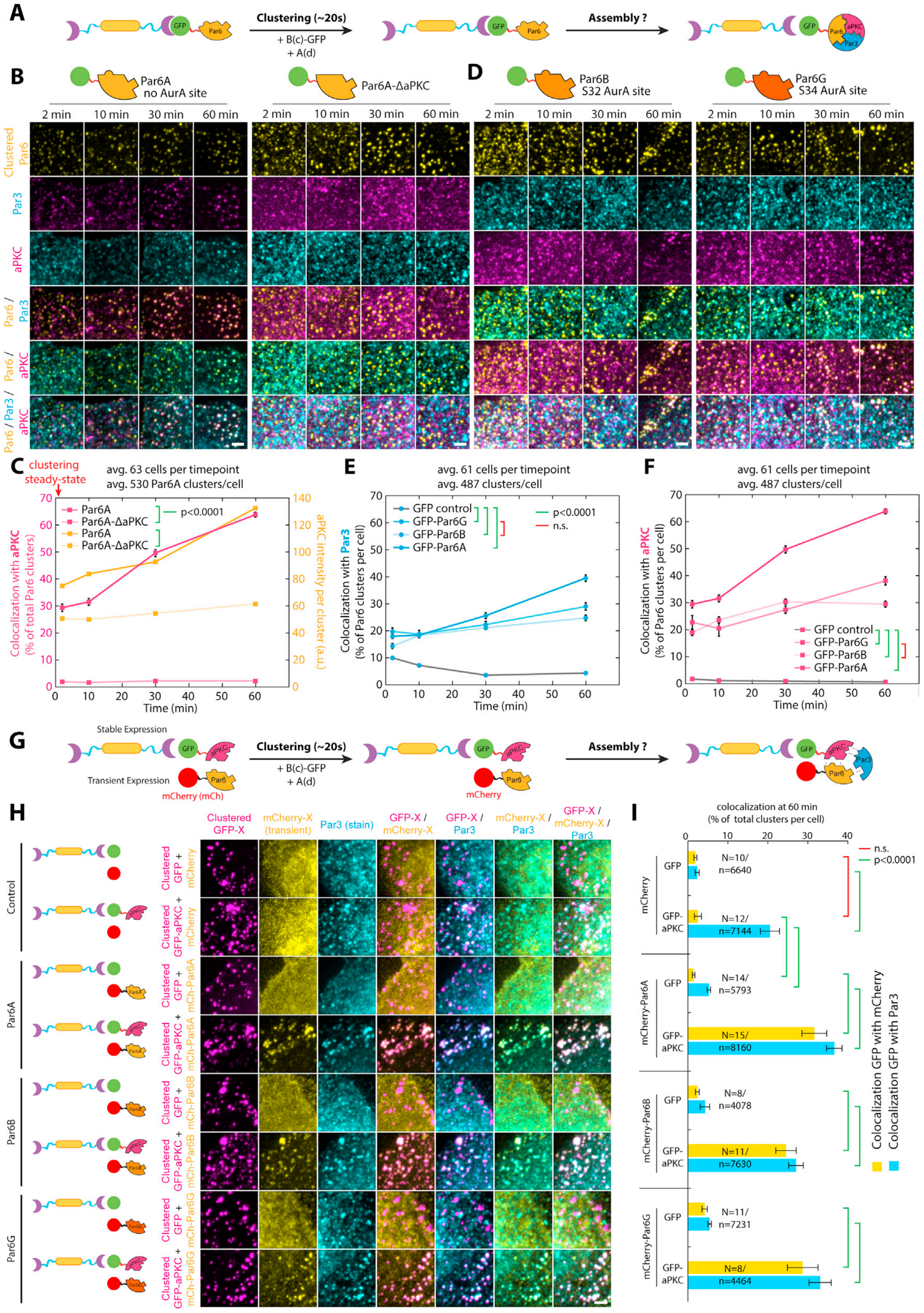


(legend on next page)

**Figure S4. Characterization of the ability of Par6 isoforms to support Par complex assembly, related to Figure 2**

(A) Principle of the experiment: 3T3 cells stably co-expressing GBP-TM-GBP and GFP-fused Par6 variants were incubated with B(c)GFP then A(d) to induce rapid clustering, and the recruitment of endogenous aPKC and Par3 was monitored by immunofluorescence.
(B) SDCM images of cells expressing indicated Par6 mutant were treated as presented in (A) and immunostained for Par3 and aPKC.
(C) Mean ± SEM percentage of colocalization between GFP-Par6A (or GFP-Par6A$^{\Delta aPKC}$) clusters and aPKC over time, plotted at the same time as the mean ± SEM intensity of aPKC per cluster over time.
(D) SDCM images of cells expressing Par6 isoforms, treated as in (A) then stained for Par3 and aPKC.
(E and F) Mean ± SEM percentage of colocalization between clusters of indicated Par6 isoform and Par3 (E) or aPKC (F). Statistics: ANOVA2 using construct and time point as variables followed by Tukey post hoc test (p value of each test indicated).
(G) Principle of the experiment: 3T3 cells stably expressing GBP-TM-GBP and GFP-fused aPKC and transiently transfected with mCherry-Par6 isoforms were incubated with B(c)GFP then A(d) to induce rapid clustering.
(H) Cells expressing indicated Par6 isoform (or controls) were treated as presented in (G), and the recruitment of exogenous Par6 and endogenous Par3 to aPKC clusters was monitored by immunofluorescence.
(I) Mean ± SEM percentage of colocalization between clusters of aPKC-GFP and indicated mCherry-fused Par6 isoform and Par3. Statistics: ANOVA1 using construct and time point as variables followed by Tukey post hoc test (p value of each test indicated).
All images in this figure were processed with a wavelet a trous filter (see STAR Methods). Scale bars, 2 μm.
See also Table 2 for definition of the constructs used in this figure.

CellPress
OPEN ACCESS

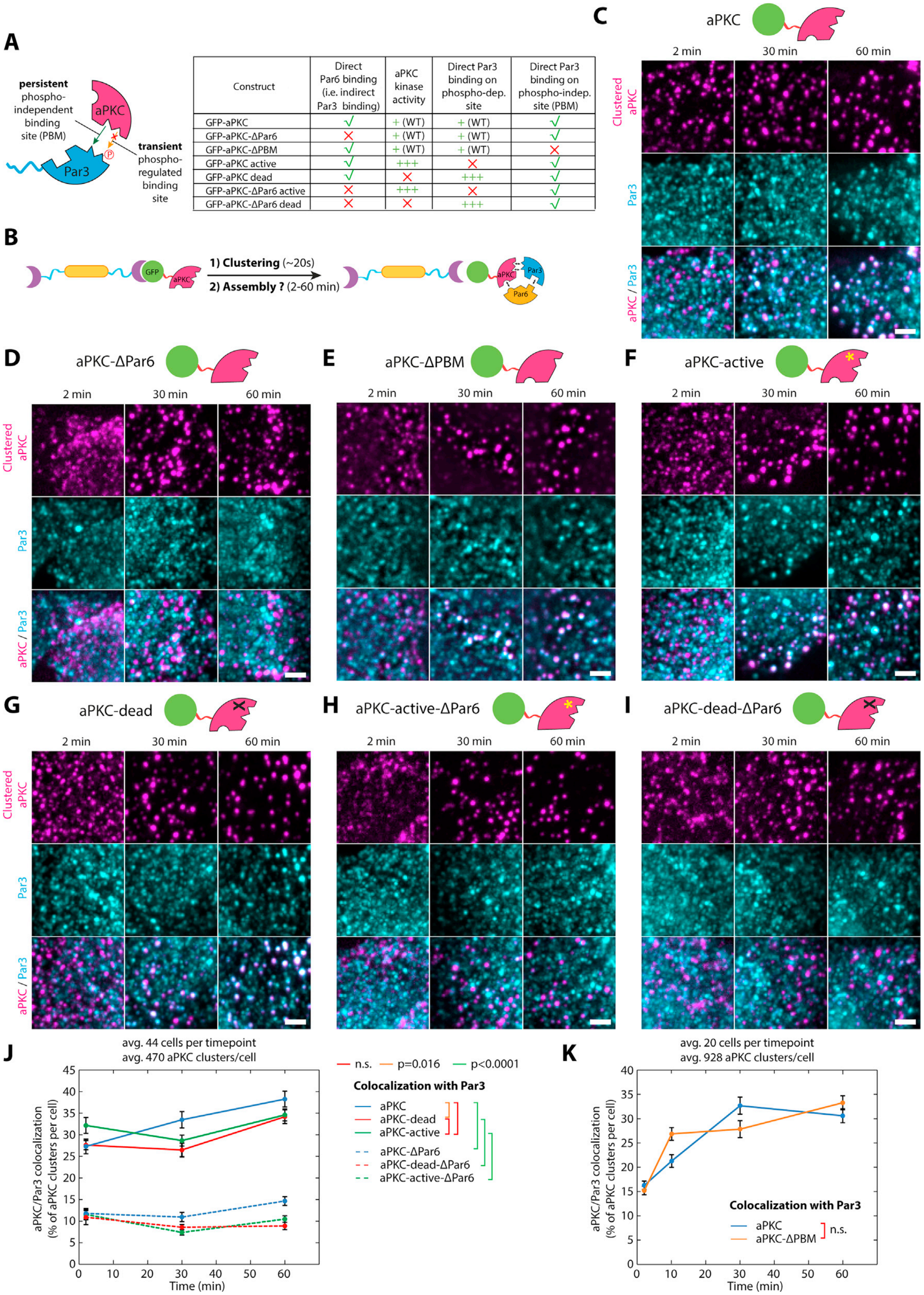


| Construct | Direct Par6 binding (i.e. indirect Par3 binding) | aPKC kinase activity | Direct Par3 binding on phospho-dep. site | Direct Par3 binding on phospho-indep. site (PBM) |
|---|---|---|---|---|
| GFP-aPKC | √ | + (WT) | + (WT) | √ |
| GFP-aPKC-ΔPar6 | ✗ | + (WT) | + (WT) | √ |
| GFP-aPKC-ΔPBM | √ | + (WT) | + (WT) | ✗ |
| GFP-aPKC active | √ | +++ | ✗ | √ |
| GFP-aPKC dead | √ | ✗ | +++ | √ |
| GFP-aPKC-ΔPar6 active | ✗ | +++ | ✗ | √ |
| GFP-aPKC-ΔPar6 dead | ✗ | ✗ | +++ | √ |

*(legend on next page)*

**Figure S5. Characterization of the effect of aPKC mutants on Par complex assembly, related to Figure 2**

(A) Constructs used in this figure. aPKC has two binding sites for Par3. One interaction takes place between the kinase domain of aPKC and the aPKC phosphorylation motif on Par3 (also known as CR3 domain). This interaction is phospho-regulated and abolished upon phosphorylation. A second site on aPKC, called PBM, has recently been shown to bind to the PDZ2 domain of Par3 in a phosphorylation-independent way.[31]

(B) Principle of the experiment: 3T3 cells stably expressing GBP-TM-GBP and GFP-fused aPKC mutants were incubated with B(c)GFP then A(d) to induce rapid clustering.

(C–I) Cells expressing indicated aPKC mutant were treated as presented in (B), and the recruitment of endogenous Par3 was monitored by immunofluorescence.

(J and K) Mean ± SEM percentage of colocalization between clusters of indicated aPKC mutant and Par3. Statistics: ANOVA2 using construct and time point as variables followed by Tukey post hoc test (p value of each test indicated). (J) and (K) come from different datasets and thus should be compared with their respective controls.

All images in this figure were processed with a wavelet a trous filter. Scale bars, 2 μm.

See also Table 2 for definition of the constructs used in this figure.

CellPress
OPEN ACCESS

A

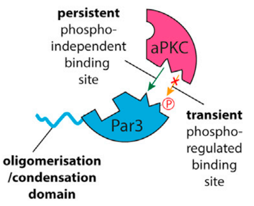


| Construct | Oligomerization / condensation | Direct Par6 binding (i.e. indirect aPKC binding) | Phospho-regulated aPKC binding site (CR3) | State of phospho-regulated aPKC binding site | predicted direct aPKC binding on phospho-regulated site | Direct aPKC binding on PDZ2 site |
|---|---|---|---|---|---|---|
| GFP-Par3 | √ | √ | √ | WT | + | √ |
| GFP-Par3-ΔaPKC1 | √ | √ | ✕ | WT | none | √ |
| GFP-Par3-SA | √ | √ | √ | phosphomutant | +++ | √ |
| GFP-Par3-SD | √ | √ | √ | phosphomimetic | - | √ |
| GFP-Par3-ΔN | ✕ | √ | √ | WT | + | √ |
| GFP-Par3-ΔN-ΔaPKC1 | ✕ | √ | ✕ | not applicable | none | √ |
| GFP-Par3-ΔN-ΔaPKC1-ΔPDZ2 | ✕ | √ | ✕ | not applicable | none | ✕ |
| GFP-Par3-ΔN-ΔaPKC1-ΔPDZ2-ΔPar6 | ✕ | low | ✕ | not applicable | none | ✕ |

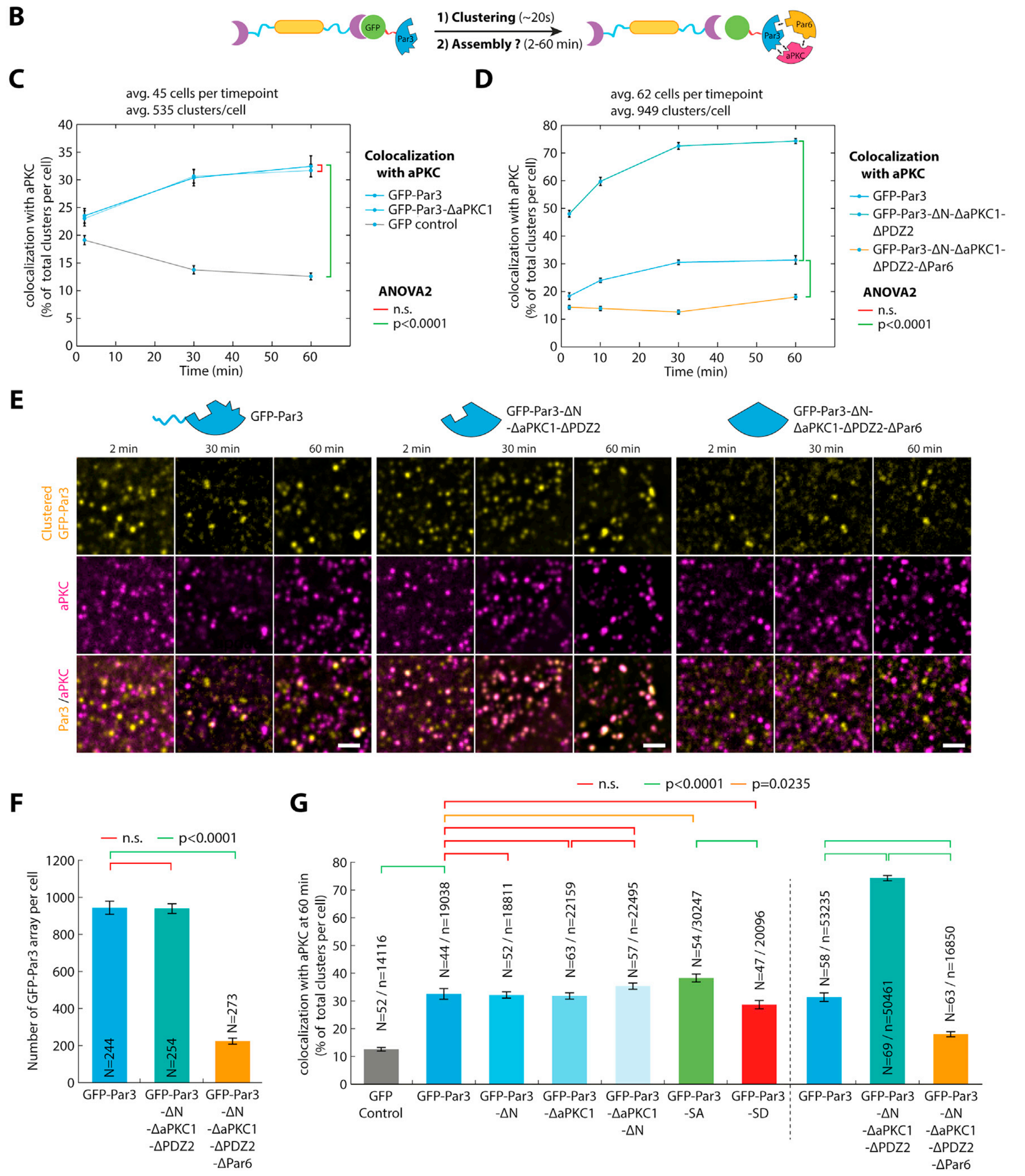


*(legend on next page)*

**Figure S6. Characterization of the effect of Par3 mutants on Par complex assembly, related to Figure 2**

(A) Description of the constructs used in this figure and their predicted effects on oligomerization, aPKC binding, and Par6 binding. Par3 has two binding sites for aPKC. One interaction takes place between the kinase domain of aPKC and the aPKC phosphorylation motif on Par3 (also known as CR3 domain). This interaction is phospho-regulated and abolished upon phosphorylation. A second site on Par3, the PDZ2 domain, binds to the PBM domain of aPKC in a phosphorylation-independent way.[31] In addition, Par3 oligomerization/condensation is known to occur via the N-terminal domain.[43]

(B) Principle of the experiment: 3T3 cells stably expressing GBP-TM-GBP and GFP-fused Par3 (or mutant thereof), were incubated with B(c)GFP then A(d) to induce rapid clustering. Then, the assembly of the endogenous Par complex was followed over time by aPKC immunofluorescence and automated quantification.

(C and D) Mean ± SEM percentage of colocalization between clusters of indicated GFP-Par3 construct and aPKC as a function of time after cluster formation (time point 0). Statistics: ANOVA2 using time and construct as variables; p value for effect of the construct indicated.

(E) Cells expressing indicated Par3 mutant were treated as in (B), and the recruitment of endogenous aPKC was monitored by immunofluorescence. Images were processed with a wavelet a trous filter. These images correspond to the samples quantified in (D). Scale bars, 2 μm.

(F) Mean ± SEM of the number of GFP-Par3 array per cell in the samples presented in (D) and (E). Statistics: Kruskal-Wallis test followed by Dunn's post hoc test (p values figured for each test, n indicates the number of cells averaged). Note that Par3-ΔN-ΔaPKC1-ΔPDZ2 has a propensity to make fewer clusters than Par3 and Par3-ΔN-ΔaPKC1.

(G) Mean ± SEM of the aPKC/Par3 colocalization 60 min post clustering for the indicated construct. Statistics: one-way ANOVA followed by Dunn's post hoc test (p values figured for each test, n indicates the number of cells averaged).

See also Table 2 for definition of the constructs used in this figure.

CellPress
OPEN ACCESS

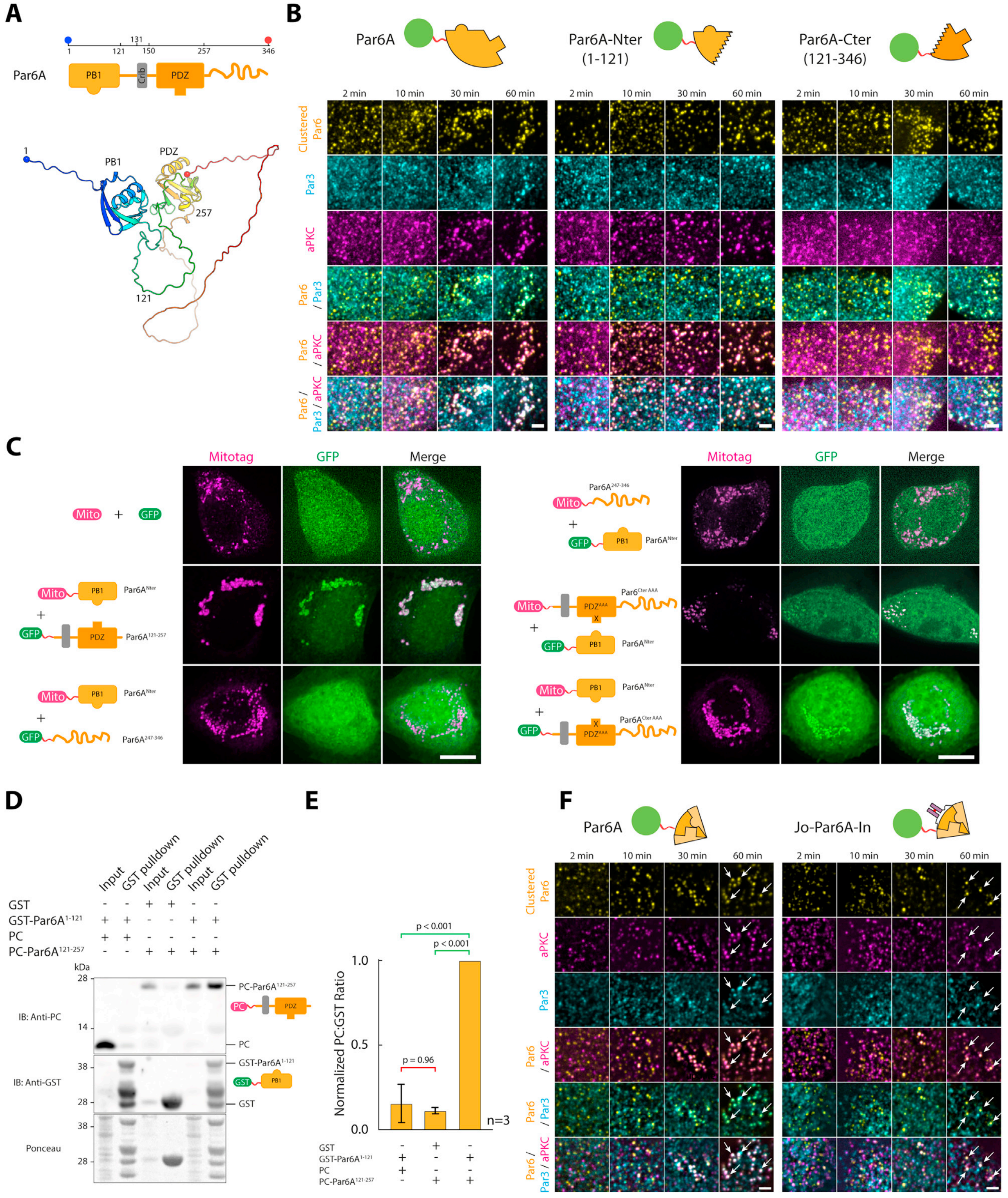


## Figure S7. Characterization of the effect of Par6A truncation on Par complex assembly and determination of the interaction between Par6A Cter and Nter, related to Figure 3

(A) Top: domain organization of Par6A, with the N-terminal PB1 domain binding to aPKC and the C-terminal PDZ domain binding to Par3. Bottom: AlphaFold2[33] structure prediction of human Par6A (https://alphafold.ebi.ac.uk/entry/Q9NPB6). Note that the AlphaFold2 prediction suggests that the N terminus (Nter) and C terminus (Cter) of Par6A interact.

(legend continued on next page)

(B) GFP-fused Par6A, or fragments thereof, were clustered in 3T3 cells as in Figure 1 and the recruitment of endogenous aPKC/Par3 was monitored by immunofluorescence. Images correspond to MIPs, and images were denoised with a Wavelet a trous filter. This corresponds to the samples analyzed in Figure 3B.

(C–E) The N-terminal domain of Par6A can bind to the C terminus.

(C) 3T3 cells co-expressing the indicated fragments of Par6A tagged with GFP or tethered to mitochondria were assessed for GFP-recruitment to the mitochondria by SDCM. Images correspond to MIPs over the entire cell. Par6A PDZ$^{AAA}$ corresponds to the Alanine mutation of AAs 169_LGF_171 in full-length Par6A, known to abolish binding of PDZ domains.

(D and E) One half of Par6A can pull-down the other when expressed in bacteria (D) GST-Par6A$^{Nter}$ and PC-tagged Par6A$^{Cter}$ were expressed independently in bacteria, and the bacterial lysates were then mixed and incubated with glutathione beads to pull-down the GST tag. The presence of PC-Par6A$^{Cter}$ was then assessed by western blot (picture representative of n = 3 experiments).

(E) Quantification of the effects seen in (D), suggesting specific pull-down of PC-Par6A$^{Cter}$ in presence of GST-Par6A$^{Nter}$.

(F) GFP-fused Par6A, or Jo-Par6A-In, were clustered in 3T3 cells, and the recruitment of endogenous aPKC/Par3 over time assessed by SDCM. Images correspond to MIPs of 2–4 z-planes (200 nm z-pitch), and images were denoised with a Wavelet a trous filter. This corresponds to the samples analyzed in Figure 3D. Note that Jo-Par6A-In recruits aPKC but not Par3 (arrows).

Scale bars: 2 μm in (B) and (F) and 10 μm in (C).

See also Table 2 for definition of the constructs used in this figure.

CellPress
OPEN ACCESS

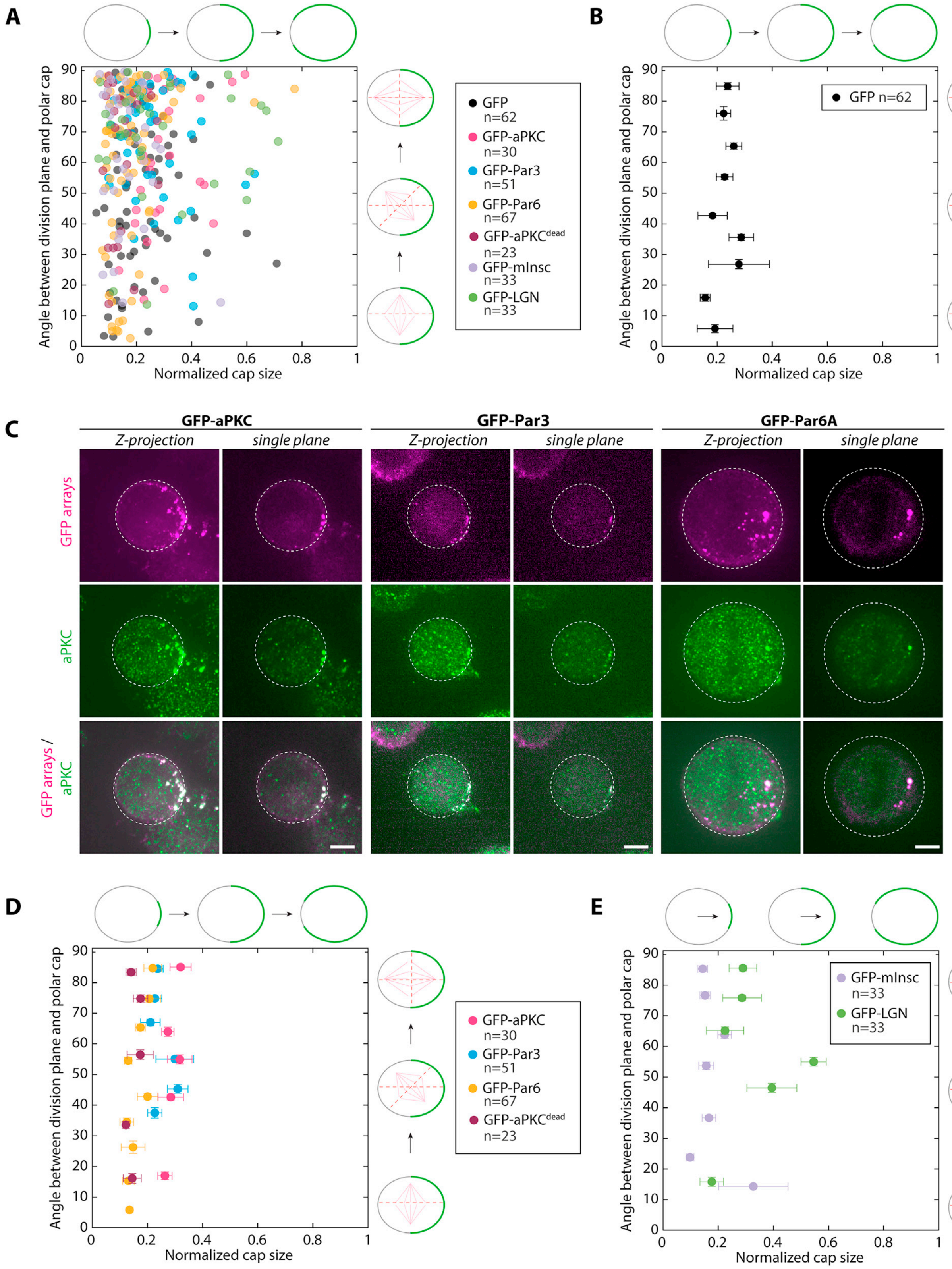


(legend on next page)

**Figure S8. Characterization of the effect of cap size on spindle orientation, related to Figure 4**

(A) Angle between the division plane and the array cap size as a function of the protein targeted to the cap in 3T3 cells stably co-expressing Jupiter-iRFP670, GBP-TM-GBP as well as indicated GFP-fusions. Cells were stalled in mitosis for >12 h after array assembly and imaged by SDCM upon release from nocodazole block. Cap size was determined in metaphase and expressed as a fraction of the cell perimeter. n: number of cells measured. This plot was used to generate Figure 4H after binning.

(B) Control GFP data presented in (A) binned according to angle values and plotted (mean ± SEM).

(C) 3T3 cells stably co-expressing GBP-TM-GBP and GFP-aPKC, GFP-Par3, or GFP-Par6A with assembled arrays immunostained for aPKC. Images correspond to MIPs or single confocal planes, as indicated. Scale bars, 10 μm.

(D and E) Data presented in (A) was binned according to angle values and plotted (mean ± SEM).

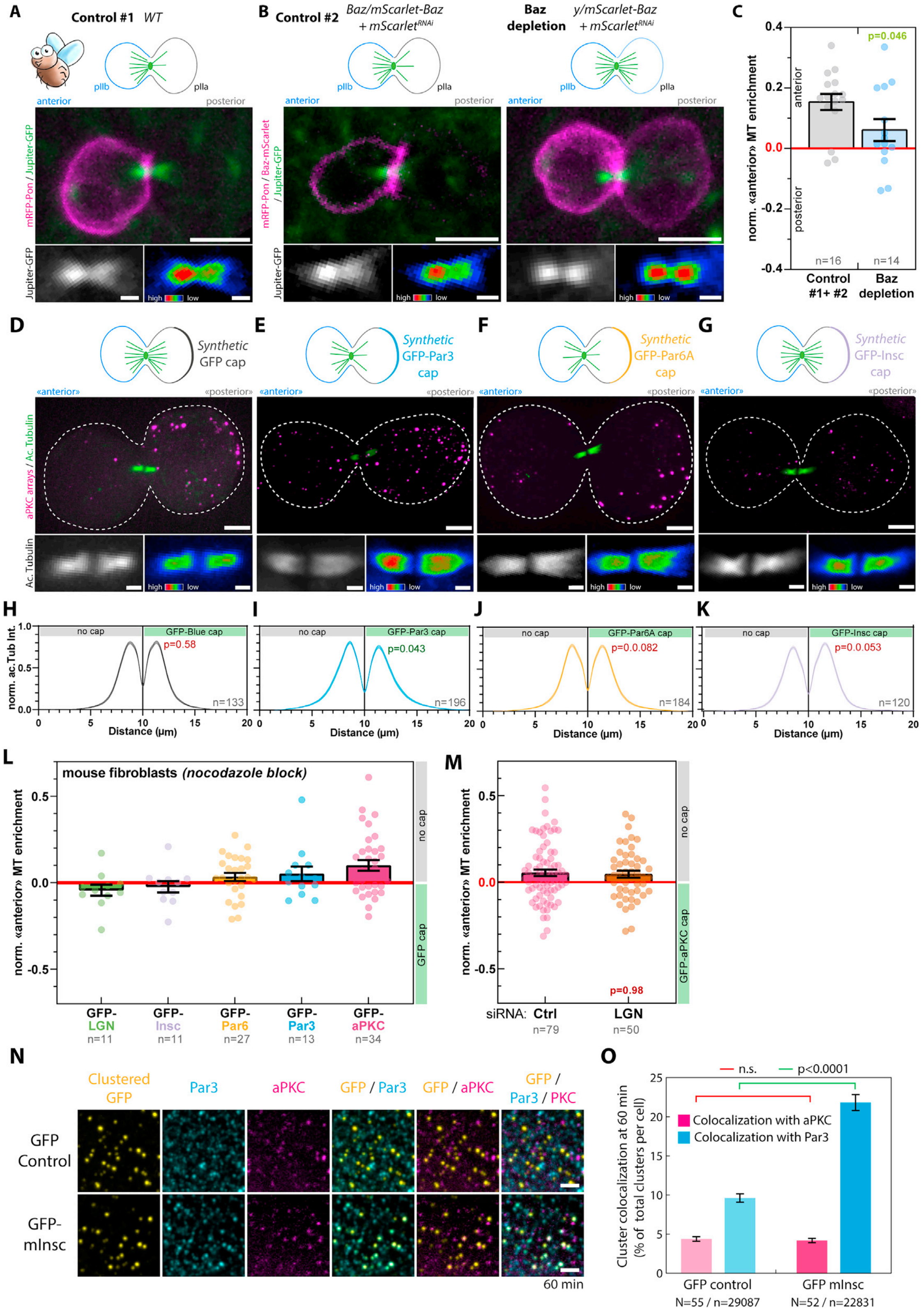


*(legend on next page)*

**Figure S9. Characterization of Par complex dependency on central spindle asymmetry in fly SOP cells and mammalian 3T3 cells, related to Figure 5**

(A–C) The Par complex is required for central spindle asymmetry in fly SOPs.

(A and B) Top rows: live SOPs of indicated genotype showing Jupiter-GFP/mRFP-Pon$^{LD}$ in anaphase (SDCM imaging, MIPs of entire cell). Bottom rows corresponds to Jupiter-GFP signal with or without the rainbow RGB LUT applied (image corresponds to MIPs of the central spindle region). Anterior/pIIb orientation is determined by the mRFP-Pon$^{LD}$ signal.

(C) Normalized Jupiter-GFP signal enrichment in pIIb (see STAR Methods) for the conditions shown in (A) and (B). statistics: unpaired t test, p value indicated, n: number of central spindles quantified.

(D–G) SDCM images (MIPs) of 3T3 cells stably expressing GBP-TM-GBP, as well as GFP-fusions with indicated proteins, processed for array assembly then selected by mitotic shake-off followed by immunostaining against acetyl-tubulin. Bottom: split-acetyl-tubulin channel with a grayscale (left) or rainbow (right) lookup table.

(H–K) Acetyl-tubulin intensity pseudo-linescan through the central spindle (mean ± SEM; n: number of cells measured) in 3T3 cells stably expressing GBP-TM-GBP and indicated GFP fusions. Statistics: paired t test between the respective peak values in each cell (p value indicated).

(L) Normalized enrichment of microtubule density on the “anterior” side of the central spindle (i.e., without cap) in cells with stably expressing GBP-TM-GBP, as well as GFP-fusions with indicated protein, stalled in mitosis for >12 h with a nocodazole block and immunostained for acetyl-tubulin.

(M) Normalized enrichment of microtubule density on the anterior side of the central spindle in cells with a GFP-aPKC cap and treated with control or LGN siRNA. Note that, given that LGN depletion affects spindle orientation (Figures 4J and 4K), we only analyzed mitotic cells in which the mitotic spindle was oriented perpendicular to the cap by chance, for analysis of central spindle asymmetry. p values were calculated using a Wilcoxon-Mann-Whitney test.

(N) Interphase 3T3 cells stably co-expressing Jupiter-iRFP670, GBP-TM-GBP as well as GFP-mInsc or GFP as a control were processed for array assembly then immunostained for endogenous Par3 and aPKC. For representation purposes, images were processed with a wavelet a trous filter.

(O) Mean ± SEM percentage of the 3D colocalization between GFP-mInsc (or GFP control) clusters and aPKC or Par3 per cell. Statistics: Student’s t test (N = number of cells quantified; n = total number of arrays quantified per condition).

Scale bars, 5 μm (A, B, and D–G, top) and 1 μm (A, B, D–G, bottom, and N).

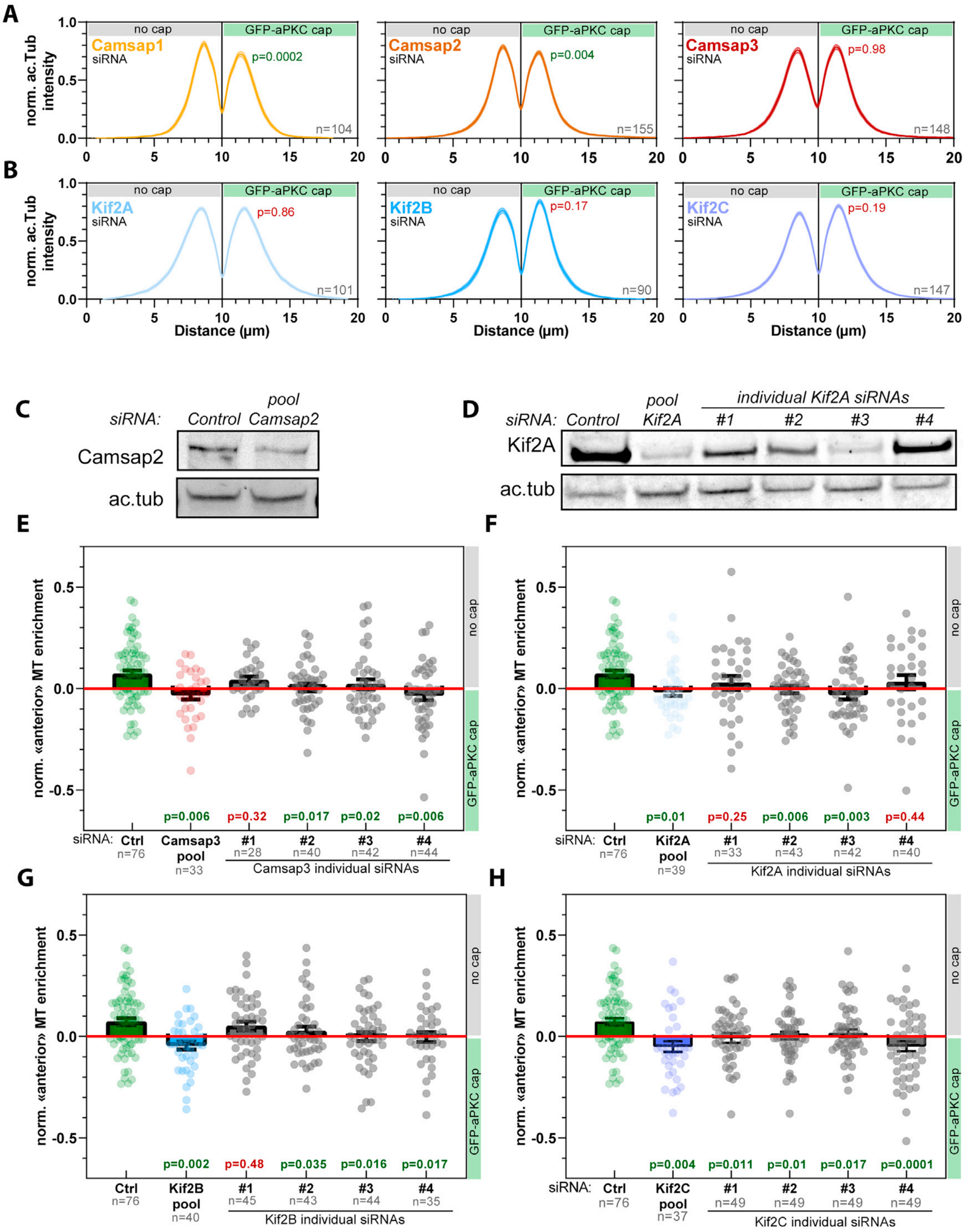


**Figure S10. Characterization of the effect of Camsap and kinesin-13 pooled and individual siRNAs on central spindle asymmetry, related to Figure 7**

(A and B) Acetyl-tubulin intensity pseudo-linescan through the central spindle (mean ± SEM; n: number of cells measured; see STAR Methods) of dividing 3T3 cells expressing GBP-TM-GBP and GFP-aPKC treated with indicated siRNA before array assembly and mitotic shake-off. Statistics: paired t test between the respective peak values in each cell (p value indicated). These data correspond to Figures 7E and 7F.

(C and D) Western blot of whole-cell lysates of 3T3 cells stably expressing GBP-TM-GBP and GFP-aPKC treated with control or Camsap2 pool siRNA (C) or with Kif2A pool siRNA and Kif2A individual siRNAs (#1–4) (D). Acetylated tubulin western blot is shown as a loading control.

(legend continued on next page)

(E–H) Normalized enrichment of microtubule density on the side opposite to the aPKC-cap of the central spindle in 3T3 cells treated with control, Camsap3 pool siRNA, and Camsap3 individual siRNAs #1–4 (E), control, Kif2A pool siRNA, and Kif2A individual siRNAs #1–4 (F), control, Kif2B pool siRNA, and Kif2B individual siRNAs #1–4 (G), or control, Kif2C pool siRNA, and Kif2C individual siRNAs #1–4 (H). p values were calculated using a Wilcoxon-Mann-Whitney test by comparing to cells treated with respective control siRNA.
